# Mediation effect and metabolic pathways of gut microbiota in the associations between lifestyles and dyslipidemia

**DOI:** 10.1038/s41522-025-00721-x

**Published:** 2025-05-28

**Authors:** Lijun Zeng, Bin Yu, Peibin Zeng, Zhuoma Duoji, Haojiang Zuo, Jian Lian, Tingting Yang, Yingxue Dai, Yuemei Feng, Peng Yu, Jiqi Yang, Shujuan Yang, Qingyu Dou

**Affiliations:** 1 Laboratory of Cardiac Structure and Function, Institute of Cardiovascular Diseases, West China Hospital, Sichuan University, Chengdu, China; 2 West China School of Public Health and West China Fourth Hospital, Sichuan University, Chengdu, China; 3 Institute for Disaster Management and Reconstruction, Sichuan University-The Hongkong Polytechnic University, Chengdu, Sichuan China; 4 High Altitude Medical Research Center, School of Medicine, Tibet University, Lhasa, China; 5 Institute of Chronic Non-Communicable Disease Control and Prevention, Chongqing Center for Disease Control and Prevention, Chongqing, China; 6 School of Public Health, Key Laboratory of Environmental Pollution Monitoring and Disease Control, Ministry of Education, Guizhou Medical University, Guiyang, China; 7 Chengdu Center for Disease Control and Prevention, Chengdu, China; 8 School of Public Health, Kunming Medical University, Kunming, China; 9 International Institute of Spatial Lifecourse Epidemiology (ISLE), Wuhan University, Wuhan, China; 10 National Clinical Research Center for Geriatrics, Center of Gerontology and Geriatrics, West China Hospital, Sichuan University, Chengdu, China

**Keywords:** Clinical microbiology, Policy and public health in microbiology

## Abstract

Whether the role of gut microbial features lies in the pathways from lifestyles to dyslipidemia remains unclear. In this cross-sectional study, we conducted a metagenome-wide association analysis and fecal metabolomic profiling in 994 adults from the China Multi-Ethnic cohort. A total of 26 microbial species were identified as mediators between lifestyle factors and risk for dyslipidemia. Specifically, the abundance of *[Ruminococcus] gnavus* mediated the associations between lifestyles and risks for dyslipidemia, elevated low-density lipoprotein cholesterol, elevated total cholesterol, and elevated triglycerides. *[Ruminococcus] gnavus, Alistipes shahii, and Lachnospira eligens* were replicated to be associated with dyslipidemia in an external validation cohort. The potential metabolic pathways included arachidonic acid, bile acid, and aromatic amino-acid metabolism.

## Introduction

With rapid economic growth and lifestyle changes, the prevalence of dyslipidemia has been estimated to be 39% globally^1^ and 35.6% in China^2^. Dyslipidemia is one of the major risk factors for atherosclerosis and is closely associated with risks for ischemic heart disease, ischemic stroke, and other atherosclerotic cardiovascular diseases^1,3^. Numerous studies have indicated that unhealthy lifestyles, including smoking, drinking, and inadequate physical activity, have contributed to elevated blood lipid levels^4–8^. However, the associations between unhealthy lifestyle factors and elevated blood lipid levels are not straightforward, and a variety of factors may contribute to their associations. For instance, dietary sphingolipids may affect the gut-liver axis by preventing the translocation of gut bacteria-derived lipopolysaccharide and inhibiting its proinflammatory effects, thereby leading to a reduction in serum lipids^9^.

Recent studies have highlighted that lifestyle factors may induce gut microbiota dysbiosis either through antimicrobial activity^10^ or by influencing the abundance of butyrate-producing bacteria^11,12^. Additionally, the gut microbiota has been suggested as a key regulator of lipid metabolism, primarily via enzymatic activities (e.g., bile salt hydrolases, cholesterol oxidases) and microbial metabolites such as short-chain fatty acids^13,14^. Building upon these mechanistic insights, the tripartite interplay among lifestyle patterns (such as obesity and diet), microbial ecosystem alterations, and host lipid metabolism has been increasingly recognized^15–17^. Nevertheless, critical knowledge gaps persist regarding: (1) the causal hierarchy in the lifestyle-microbiota-dyslipidemia axis, (2) the combinatorial effects of clustered lifestyle risks (unhealthy lifestyles often cluster and have synergistic effects in real-world scenarios)^18,19^, and (3) the operationalization of lifestyle assessment in epidemiological contexts. Therefore, it is necessary to employ a comprehensive index of lifestyle and a causal inference model to obtain more objective results.

This study proposes a causal mediation framework in which specific gut microbial features mediate the relationship between lifestyle exposures (smoking, drinking, etc.) and dyslipidemia. To test this hypothesis, we conducted a population-based survey using multi-omics data from the China Multi-Ethnic Cohort (CMEC)^20,21^, and aimed to investigate the possible mediation effect of gut microbiota on the association between lifestyle factors and dyslipidemia. The findings were validated in an independent external cohort, the Healthy Lifestyle Promotion Cohort (HLPC)^22^, conducted in four counties in Sichuan province. Utilizing shotgun sequencing and metagenomics, this study specifically aimed to: (1) Identify lifestyle-microbiota-dyslipidemia interaction using multi-omics integration; (2) Quantify the mediation effects of microbial species and functional modules using causal inference models; (3) Validate candidate mediators through cross-cohort replication. Our findings may pioneer microbiota-directed strategies to mitigate cardiovascular risks, particularly for individuals with entrenched unhealthy lifestyles.

## Results

### Baseline characteristics in discovery cohort

**Fig. 1:**
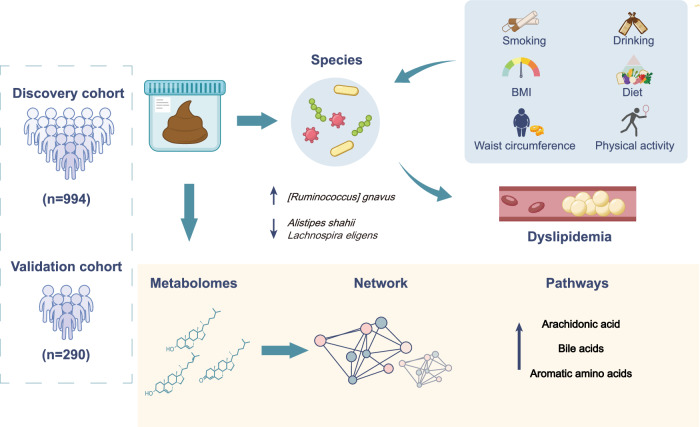
Study design. Schematic of study design and data collection. BMI body mass index.

**Fig. 2:**
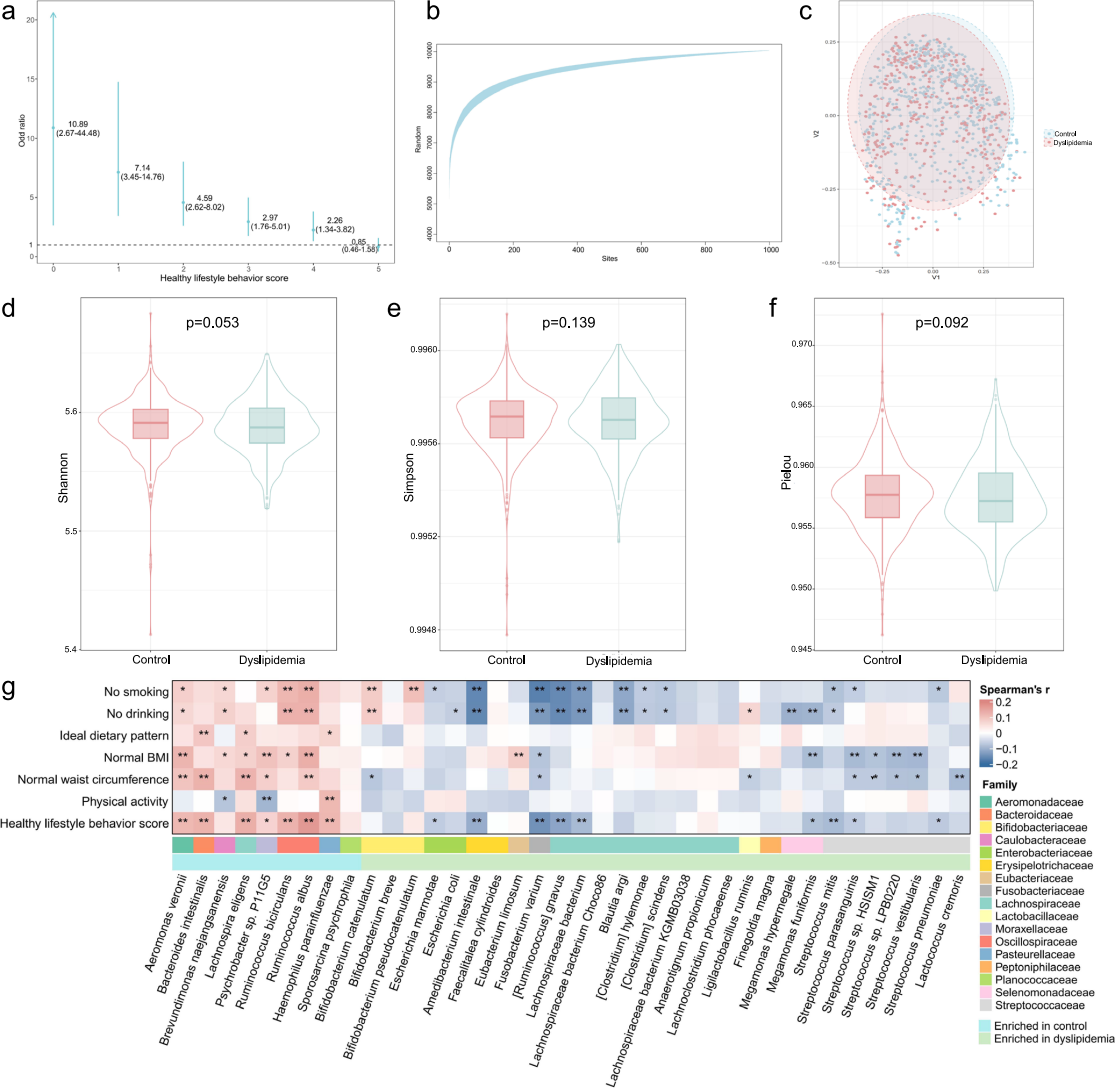
Microbial features contributing to dyslipidemia in discovery cohort. **a** Odd ratios of healthy lifestyle behavior score related to dyslipidemia (adjusted by age, sex, ethnicity, education, and marriage). **b** Species accumulation curve based on total numbers of species. **c** Principal coordinates analysis (PCoA) plot dependent on Bray–Curtis distances shows that dyslipidemia and healthy people have essentially different gut microbiome profiles as PERMANOVA (*R*^2^ = 0.002, *P* < 0.001). **d**–**f** Alpha diversity assessed by shannon, simpson, and pielou index. **g** Differential species significantly associated with healthy lifestyle factors. *P < 0.05; **P < 0.01.

The study was conducted in CMEC (discovery cohort) and validated in HLPC (Fig. 1). In the discovery cohort, 994 individuals had a mean age of 53.0 ± 11.5 years, with 49.9% being female (Table 1). Among them, 373 (37.5%) were diagnosed with dyslipidemia, including 187 (18.8%) with elevated TG, 102 (10.3%) with elevated TC, 107 (10.8%) with elevated LDL-C, and 153 (15.4%) with decreased HDL-C. Participants with dyslipidemia exhibited a lower lifestyle behavior score (3.1 ± 1.4 vs 4.0 ± 1.4, *p* < 0.001), a higher prevalence of male (60.9% vs 43.6%, *p* < 0.001), ethnic minorities (15.0% vs 6.1%, *p* < 0.001), smoking (34.1% vs 20.6%, *p* < 0.001) and drinking (27.9% vs 18.5%, p < 0.001), and a lower prevalence of normal BMI (22.3% vs 51.2%, *p* < 0.001) and normal waist circumference (23.3% vs 51.4%, *p* < 0.001). No significant differences were observed in dietary pattern and moderate-to-high intensity physical activity. After adjusting for covariates, the lifestyle behavior score showed a negative association with dyslipidemia risk (Fig. 2a).

**Table 1:** Baseline characteristics in discovery cohort

	Total (*n* = 994)	Control (*n* = 621)	Dyslipidemia (*n* = 373)	*P* value
Sex (%)				<0.001
Female	496 (49.9%)	350 (56.4%)	146 (39.1%)	
Male	498 (50.1%)	271 (43.6%)	227 (60.9%)	
Age, years (mean ± SD)	53.0 ± 11.5	52.9 ± 12.0	53.2 ± 10.4	0.679
Ethnicity (%)				<0.001
Han	900 (90.5%)	583 (93.9%)	317 (85.0%)	
Others	94 (9.5%)	38 (6.1%)	56 (15.0%)	
Education (%)				0.806
Not attending school	101 (10.2%)	61 (9.8%)	40 (10.7%)	
Primary or high school	714 (71.8%)	445 (71.7%)	269 (72.1%)	
College or above	179 (18.0%)	115 (18.5%)	64 (17.2%)	
Marial status (%)				0.527
Married	904 (91.0%)	562 (90.5%)	342 (91.7%)	
Unmarried or divorced	90 (9.1%)	59 (9.5%)	31 (8.3%)	
Smoking (%)				<0.001
Never	739 (74.3%)	493 (79.4%)	246 (66.0%)	
Currently / Previous	255 (25.7%)	128 (20.6%)	127 (34.1%)	
Drinking (%)				<0.001
Never / Rarely	775 (78.0%)	506 (81.5%)	269 (72.1%)	
Frequent	219 (22.0%)	115 (18.5%)	104 (27.9%)	
Ideal dietary patterns (%)				0.997
No	397 (39.9%)	248 (39.9%)	149 (40.0%)	
Yes	597 (60.1%)	373 (60.1%)	224 (60.1%)	
DASH score	21.8 ± 4.5	22.0 ± 4.4	21.5 ± 4.6	0.064
Normal body mass index (%)				<0.001
No	593 (59.7%)	303 (48.8%)	290 (77.8%)	
Yes (18.5 ~ 23.9 kg/m^2^)	401 (40.3%)	318 (51.2%)	83 (22.3%)	
Normal waist circumference (%)				<0.001
No	588 (59.2%)	302 (48.6%)	286 (76.7%)	
Yes (male < 85 cm, female < 80 cm)	406 (40.9%)	319 (51.4%)	87 (23.3%)	
Moderate-high intensity physical activity (%)				0.081
No	285 (28.7%)	166 (26.7%)	119 (31.9%)	
Yes ( > 75 minutes per week)	709 (71.3%)	455 (73.3%)	254 (68.1%)	
Healthy lifestyle behavior scores	3.7 ± 1.4	4.0 ± 1.4	3.1 ± 1.4	<0.001

*DASH* dietary approach to stop hypertension.

### Microbial and function signatures in subjects with dyslipidemia

In the discovery cohort, the species accumulation curve showed the adequate of the microbial species (Fig. 2b). The most abundant species in participants with or without dyslipidemia were *Phocaeicola vulgatus*, *Faecalibacterium prausnitzii, Bacteroids ovatus*, etc (Supplementary Fig. 1). Rarefaction curves of the samples analyzed attained the saturation plateau, indicating that the sequencing depth was sufficient to evaluate the bacterial diversity (Supplementary Fig. 2). All the α diversity parameters of gut microbiota were not significantly different between participants with and without dyslipidemia, while a significant difference was found in *β* diversity (*p* < 0.001, *R*^2^ = 0.002, Fig. 2c–f, Supplementary Fig. 3).

A total of 38 microbial species were identified to be associated with dyslipidemia after adjustment, 30 of which were associated with healthy lifestyles (Supplementary Tables 1–5 and Fig. 2g). Most of the microbial species were from *Lachnospiraceae* and *Streptococcaceae* family. We identified 5264 co-abundances with an FDR cutoff of 0.05. We also defined central nodes ranked in the top 10% in the number of node degrees as keystone species, with Alistipes shahii identified as core species (Supplementary Fig. 4, Supplementary Table 6).

To further understand the dyslipidemia-associated microbial functions, we found 10 pathways (e.g., 4-hydroxybenzoate biosynthesis, L-lysine degradation, allantoin degradation to glyoxylate II, Supplementary Table 7 and Supplementary Fig. 5). 4-hydroxybenzoate contributes to the production of coenzyme Q (CoA), and disruptions in this pathway can affect β-oxidation of fatty acids, which is crucial for mitochondrial energy metabolism^23^. L-lysine degradation and glyoxylate produce acetyl-CoA, which is a central intermediate in both fatty acid synthesis and degradation ^24^.

### Mediating role of the gut microbiota

**Fig. 3:**
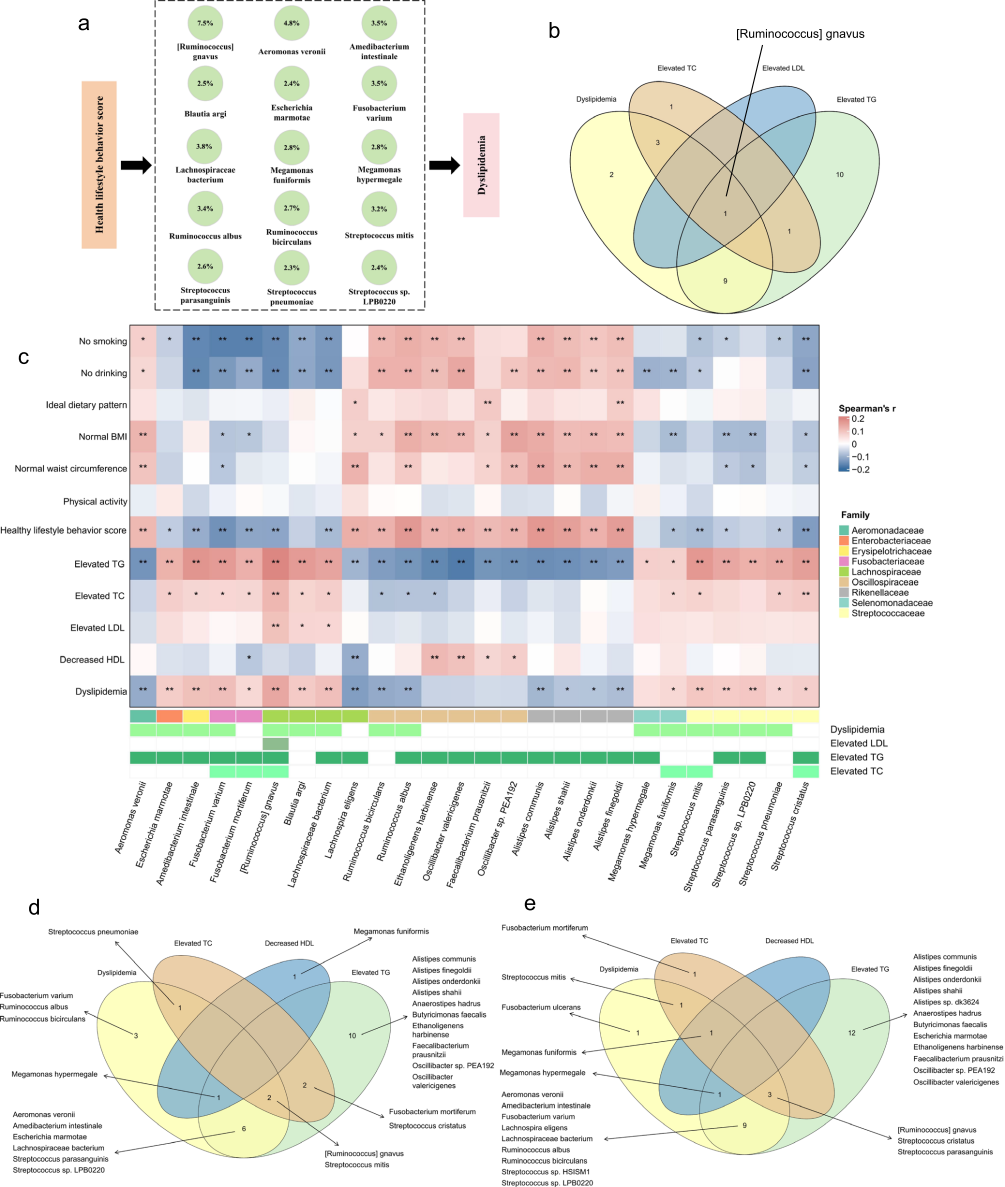
Species mediating the associations between lifestyles and risk for dyslipidemia. **a** Proportion of mediating effect of species. **b** The Venn plot shows overlapped mediating species in subtypes of dyslipidemia. **c** Heatmap of the Spearman’s correlation between mediating species and healthy lifestyle factors. **d** The Venn plot shows overlapped mediating species after exclusion of those who received anti-hyperlipidemia therapy. **e** The Venn plot shows overlapped mediating species after exclusion of those who were ethnic minorities. *P < 0.05; **P < 0.01.

A total of 26 microbial species mediating dyslipidemia through lifestyles were identified. We found that 15 species mediated the association between lifestyle factors and dyslipidemia, with *[Ruminococcus] gnavus* (proportion mediated: 7.5%), *Aeromonas veronii* (4.8%), *Lachnospiraceae bacterium* (3.8%), *Ruminococcus albus* (3.5%), *Fusobacterium varium* (3.5%), *Amedibacterium intestinale* (3.5%), *Streptococcus mitis* (3.2%), *Megamonas hypermegale* (2.8%), *Megamonas funiformis* (2.8%), *Ruminococcus bicirculans* (2.7%), *Streptococcus parasanguinis* (2.6%), *Blautia argi* (2.5%), *Streptococcus sp. LPB0220* (2.4%), *Escherichia marmotae* (2.4%) and *Streptococcus pneumonia* (2.3%) mediating the their associations (Fig. 3a, Supplementary Tables 8–12). Regarding subtypes of dyslipidemia, the association between lifestyle and elevated LDL-C was mediated by *[Ruminococcus] gnavus* (5.4%). Association between lifestyle and elevated TC was mediated by *[Ruminococcus] gnavus* (5.8%), *Fusobacterium mortiferum* (3.9%), *Streptococcus mitis* (3.8%), *Fusobacterium varium* (3.3%), *Megamonas funiformis* (3.3%), and *Streptococcus cristatus* (3.1%). Association between lifestyle and elevated TG was additionally influenced by *Ethanoligenens harbinense* (3.6%), *Faecalibacterium prausnitzii* (3.5%), *Lachnospira eligens* (2.9%), *Alistipes shahii* (3.2%), *Alistipes communis* (3.7%), *Alistipes finegoldii* (2.4%), *Alistipes onderdonkii* (2.3%), *Oscillibacter sp. PEA192* (2.7%) and *Oscillibacter valericigenes* (3.8%). Of note, *[Ruminococcus] gnavus* had a mediation effect on the associations between lifestyles and risks for elevated LDL-C, TC, and TG (Fig. 3b). Figure 3c showed the associations between the mediating species and lifestyle factors. Among these mediating species, *Alistipes*, *Escherichia*, *Fecalibacterium, Fusobacterium*, *Megamonas*, *Oscillibacter*, *Ruminococcus*, and *Streptococcus* have been reported to be related to obesity or metabolite diseases ^25–29^.

### Dyslipidemia-related metabolites and integrity analysis

**Fig. 4:**
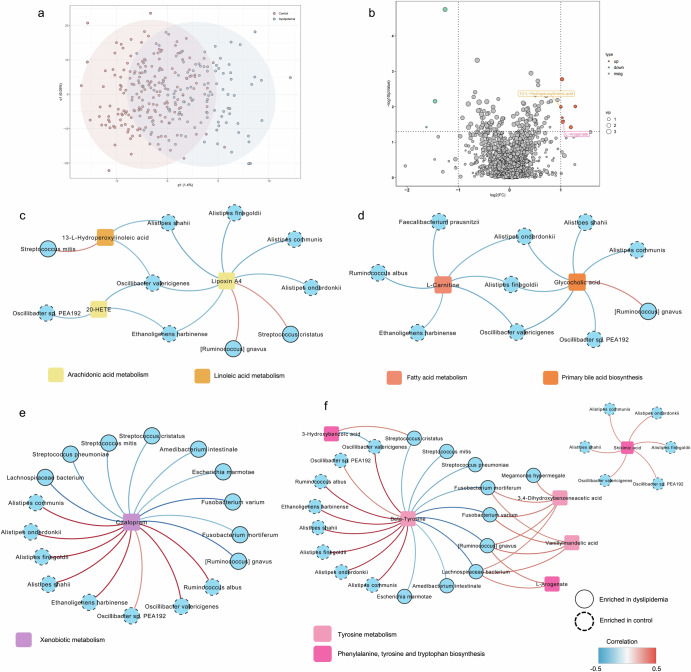
Metabolic pathways of mediating species in the associations between lifestyles and risk for dyslipidemia. **a** Score plot for orthogonal partial least squares-discriminant analysis. **b** Volcano plot shows the distribution of differential metabolites. **c**–**f** Spearman’s correlation coefficients between mediating species with dyslipidemia-related metabolites (absolute correlation coefficient > 0.25 were shown).

A total of 1695 fecal metabolites were annotated. Figure 4a, b showed differential metabolites, which were involved in linoleic acid metabolism and phenylalanine, tyrosine, and tryptophan (aromatic amino acids, AAAs) metabolism (Supplementary Table 13). The results in subgroups additionally included metabolites involved in arachidonic acid metabolism, primary bile acid biosynthesis, and xenobiotics metabolism (Supplementary Tables 14–17, Supplementary Fig. 6).

Lipoxin A4 and 20-HETE were lipid mediators derived from arachidonic acid. 13-L-Hydroperoxylinoleic acid (or 13-HPODE) was a major fatty acid oxidation product of linoleic acid and found to be esterified to cholesterol^30^. These metabolites were increased in dyslipidemia group and negatively correlated with control-enriched species, and positively correlated with *[Ruminococcus] gnavus*, *Streptococcus mitis*, and *Streptococcus cristatus* (Fig. 4c). Arachidonic acid, a polyunsaturated fatty acid, converts to Lipoxin A4 by lipoxygenase (LOX) pathway^31^. It also converts to 16(R)-HETE, 8,9-DiHETrE, 20-HETE by cytochrome P-450 (CYP) pathway^31,32^. Both pathways participate in adipose tissue inflammation, lipid dysfunction, and liver diseases ^33–35^.

The control-enriched species were negatively correlated with L-carnitine and glycocholic acid, while *[Ruminococcus] gnavus* was positively correlated with glycocholic acid (Fig. 4d). L-carnitine involves in mitochondrial β-oxidation and fatty acid metabolism, and generates trimethylamine, which is further processed to trimethylamine N-oxide (TMAO) in liver^36^. TMAO contributes to atherosclerosis by inhibiting reverse cholesterol transport^37^ and by inducing atherosclerosis-promoting inflammatory proteins in vascular cells^38^. Glycocholic acid, one of the conjugated primary bile acids, was found to increase with a high-TC diet and positively correlated with TG, TC, and LDL levels^39^, while its level can be reduced by probiotics intake^40,41^. Increased glycocholic acid inhibits farnesoid X receptor (FXR), thus stimulating TC de novo synthesis by induction of CYP7A1 activity ^42,43^.

Citalopram, involved in xenobiotic metabolism, was positively correlated with control-enriched species, and negatively correlated with dyslipidemia-enriched species (Fig. 4e).

Figure 4f illustrated the correlations between mediating species and metabolites involved in AAAs metabolism. Beta-tyrosine is not directly involved in the core metabolic pathways of tyrosine, while its decreased level may reflect phenylalanine/tyrosine metabolism disorder^44^. The increased Vanillylmandelic acid, 3,4-Dihydroxybenzeneacetic acid, 3-Hydroxybenzoic acid, and L-arogenate are metabolites of AAAs, and are positively correlated with dyslipidemia-enriched species. AAAs are known risk factors for cardiovascular diseases and have been found enriched in obesity individuals^45^. By inhibiting intestinal FXR, AAAs increased hepatic bile acid synthesis, leading to increased body weight and white adipose tissue^46^. Shikimic acid, a key intermediate in AAAs metabolic pathway, primarily enters the human body through diet. Shikimic acid has hypolipogenic effect by attenuating the mRNA expression of de novo lipogenesis related genes (FAS, SREBP-1c, and LXR-α) and activating phosphorylation of AMP-activated protein kinase /acetyl-CoA carboxylase ^47^.

### Sensitivity analysis

The mediating species were stable in the sensitivity analysis when excluding those receiving anti-hyperlipidemia therapy (Supplementary Tables 18–27 and Fig. 3d), and excluding ethnic minorities (Supplementary Tables 33–40 and Fig. 3e). The differential metabolites were also stable, which were involved in linoleic acid metabolism, arachidonic acid metabolism, AAAs metabolism, bile acid and xenobiotics metabolism (Supplementary Tables 28–32 and 41–45).

### Validation

**Fig. 5:**
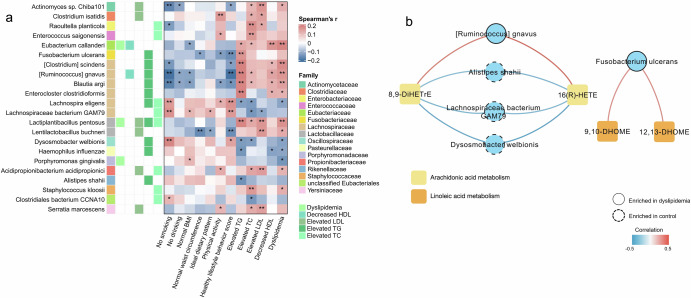
Differential species and metabolic pathways in validation cohort. **a** Heatmap of the Spearman’s correlation between differential species and clinical indices. **b** Spearman’s correlation coefficients between dyslipidemia-related species with metabolites (absolute correlation coefficient >0.25 were shown). *P < 0.05; **P < 0.01.

The HLPC included 290 adults, with 53.8% females and a mean age of 57.5 ± 11.6 years. Ninety-eight (33.8%) subjects were diagnosed as dyslipidemia, including 31 with decreased HDL-C, 19 with elevated LDL-C, 48 with elevated TC, and 58 with elevated TG. The species accumulation curve and diversity analysis in validation cohort are illustrated in Supplementary Fig. 7. A total of 22 differential microbial species were identified (Fig. 5a, Supplementary Tables 46–50). *[Ruminococcus] gnavus*, *Lachnospira eligens*, and *Alistipes shahii* were replicated as the mediating species identified in the discovery cohort. *Blautia argi*, *Clostridiales bacterium CCNA10*, *Clostridium isatidis*, *[Clostridium] scindens*, *Dysosmobacter welbionis*, *Eubacterium callanderi*, *Enteroclostridium clostridioformis*, *Fusobacterium ulcerans*, *Haemophilus influenzae*, *Porphyromonas gingivalis*, *Raoultella planticola* were identified as differential species associated with dyslipidemia, which overlapped with the differential species in subtypes of dyslipidemia in the discovery cohort.

A total of 1226 fecal metabolites were identified in HLPC. Dyslipidemia-related metabolites were mainly related to arachidonic acid and linoleic acid metabolism (Supplementary Tables 51–55). 9,10-DHOME and 12,13-DHOME, epoxygenase hydrolase products of linoleic acid^48^, were positively correlated with *Fusobacterium ulcerans*. 16(R)-HETE and 8,9-DiHETrE, metabolites of arachidonic acid, were negatively correlated with *Alistipes shahii*, *Dysosmobacter welbionis*, *Lachnospiraceae bacterium GAM79*, and positively correlated with *[Ruminococcus] gnavus* (Fig. 5b). Similar to the discovery cohort, *Alistipes shahii* and *[Ruminococcus] gnavus* exhibited opposing correlations with metabolites involved in arachidonic acid metabolism.

## Discussion

In the present study with gut microbiota and lifestyle data, we identified 26 microbes that mediated the association between lifestyle and blood lipid levels in the discovery cohort. Among them, associations of dyslipidemia with three microbes *([Ruminococcus] gnavus, Alistipes shahii, and Lachnospira eligens*) were replicated in the validation cohort.

*[Ruminococcus] gnavus*, reclassified as *Mediterraneibacter gnavus*, was identified as a key mediator^49^. Its abundance is positively associated with animal product-rich diets^50^, and negatively associated with healthy dietary^51^. In the present study, *[Ruminococcus] gnavus* was found to be additionally associated with smoking, alcohol consumption, BMI, and the overall lifestyle behavior score. *[Ruminococcus] gnavus* has been found associated with an increasing number of inflammatory bowel diseases, neurological disorders, and metabolic diseases^49^. The underpinning molecular mediators include short-chain fatty acids and secondary metabolites. *[Ruminococcus] gnavus* may produce acetate, propionate, and a small amount of lactate, but not butyrate^49^. The secondary metabolites include bile acid and tryptophan metabolites, thereby contribute its role in the gut-liver and gut-brain axis^49^. *[Ruminococcus] gnavus* is one of the microbiota that transforms primary bile acids in the intestine into secondary bile acids. These secondary bile acids profoundly impact lipid metabolism as regulatory molecules through FXR and pregnane X receptor, along with their downstream signal pathways. By the action of a tryptophan decarboxylase enzyme, *[Ruminococcus] gnavus* converts tryptophan into tryptamine, which can activate aryl hydrocarbon receptor signaling and promote systemic inflammation ^52^.

Other mediating species can also regulate lipid metabolism by influencing enterohepatic circulation. Sulfoconjugation of the toxic secondary bile acids (especially lithocholic acid) would protect hepatic, biliary, and intestinal tissues^53^, as well as improve glucose homeostasis and weight loss^54^. While the *Fusobacterium* is responsible for bile acid desulfation^55^, which results in the lengthened half-life of bile acids. In contrast, *Faecalibacterium, Alistipes, and Ruminococcus* play a role on cholesterol conversion into coprostanol making it easily eliminable with defection and reducing its absorption^56^. Besides the bile acid metabolism, *Lachnospira eligens*^11^, *Faecalibacterium prausnitzii*^57^, and *Oscillibacter* species^58^ have been reported to produce butyrate, and their abundance could be increased by exercise.

*Alistipes shahii* was negatively correlated with elevated TG in both cohorts. *Alistipes* is a relatively new genus of anaerobic gram-negative bacteria, isolated primarily from medical clinical samples^59^. *Alistipes* species have protective effects against some diseases, including liver fibrosis, colitis, cancer immunotherapy, and cardiovascular disease^59^. In a multi-regional obese population, the abundance of *Alistipes finegoldii* and *Alistipes shahii* decreased compared to non-obese controls^25^. Both species are resistant to 20% bile and can hydrolyze tryptophan to indole^59^. Notably, the decreased abundance of *Alistipes shahii* in the obesity population has been proved to be negatively correlated to AAAs^27^. Its influence on AAAs is partly attributed to the ability to hydrolyze tryptophan.

In a study about inflammatory bowel disease, *Alistipes shahii* and *Alistipes finegoldii* were positively correlated with caprylic acid, a medium-chain fatty acid with an inhibitory effect on the growth of *[Ruminococcus] gnavus*^60^. This finding suggests a potential antagonistic effect between *Alistipes* and *[Ruminococcus] gnavus*. A similar antagonistic effect was found between *Alistipes shahii* and *[Ruminococcus] gnavus* in atherosclerotic cardiovascular diseases^61^. Our study also revealed the antagonistic effect between *Alistipes shahii* and *[Ruminococcus] gnavus* in mediating dyslipidemia via lifestyles. However, the direct mechanisms underlying their interactions remain unexplored, and we hope future studies will address this gap using controlled experimental approaches.

Adherence to lifestyle changes, such as dietary modifications and exercise, can be challenging, especially among older adults. High cost, side effects, and the existence of multiple comorbidities often lead to low adherence to medications for dyslipidemia^62^. Therefore, microbiota-targeted interventions could provide a more sustainable solution for the prevention of dyslipidemia. In clinical settings, fecal microbiota transplantation, or probiotics combined with medications, could enhance lipid-lowering effects^63,64^. For public health, educational campaigns promoting gut-friendly diets and lifestyle modifications could be implemented to reduce the population-level burden of dyslipidemia. However, further research is needed to validate these findings and explore the mechanisms underlying these effects.

We have to acknowledge some limitations. First, because of the cross-sectional study design, a causal relationship between lifestyles and dyslipidemia over time cannot be established. Future longitudinal studies could address this limitation by tracking participants over time to observe the temporal sequence of lifestyle changes and the development of dyslipidemia. Second, both cohorts were from China, the inclusion of a widespread population may be needed to verify the stability of the findings. Third, the sample size in the validation cohort was small, which might have limited our ability to detect dyslipidemia-related species. Despite these limitations, this study provides new evidence on the mediating role and potential mechanisms of the gut microbiota in the associations between multiple lifestyle factors and dyslipidemia.

In conclusion, our study demonstrates the pivotal role of the gut microbiome in mediating the relationship between lifestyle factors and dyslipidemia, including its subtypes. Our findings suggest that *[Ruminococcus] gnavus, Lachnospira eligens,* and *Alistipes shahii* are key microbes involved in the pathway linking lifestyle factors to dyslipidemia.

## Methods

### Study design and participants

The research was conducted as a cross-sectional study. The discovery cohort, CMEC, completed the baseline survey in 2018 with participants recruited from five provinces in Southwest China^20,21^^,^^65^, aimed to investigate the disease burden and risk factors affecting residents in this region. During the first wave of follow-up in 2020, 1603 participants provided stool samples, of which 554 were excluded due to reporting use of antibiotics or probiotics within 1 month before stool sample collection, and 1049 were further used for metagenomic sequencing. The present study excluded 31 individuals due to the absence of blood biochemistry testing results and 24 individuals due to missing lifestyle information. As a result, a total of 994 individuals were included for analysis.

The protocol was repeated in HLPC, used here as the validation cohort^22^. The HLPC recruited participants in 2018 (at baseline) from four counties in Sichuan Province, China, using a multi-stage random sampling approach. Follow-up assessments were conducted in 2020 and 2023. In the 2023 wave, 370 of them provided stool samples. We excluded 65 people who used antibiotics and 15 participants with missing information, and 290 participants were included for analysis.

The protocol of CMEC was approved by the Sichuan University Medical Ethical Review Board (K2016038), and the HLPC was approved by Ethic Review Committee of West China Tianfu Hospital, Sichuan University (2022 [006]). All participants provided written informed consent before enrollment.

### Measurement of lifestyles

The lifestyle behavior score was measured by assessing six lifestyle behavior factors, including tobacco use, alcohol intake, physical activity, dietary pattern, waist circumference, and body mass index^66^. Never smokers were scored as 1, and 0 otherwise. Individuals who never or rarely consume alcohol were scored as 1, and 0 otherwise. Physical activity was scored as 1 for those engaging in a moderate-high intensity physical activity^67^ for more than 75 min per week, and 0 otherwise. An ideal dietary pattern^18^ was scored as 1, and 0 otherwise. A normal waist circumference of less than 85 cm for males and less than 80 cm for females was scored as 1, with 0 assigned otherwise. A normal body mass index (BMI) of 18.5 ~ 23.9 kg/m^2^ was scored as 1, and 0 otherwise. Among these factors, physical activity (PA) was assessed with the International Physical Activity Questionnaire^68,69^. PA was quantified using a metabolic equivalent (MET) value by summing recreation, transportation, occupation, and household activities, weighted by intensity and duration^70,71^. The ideal dietary pattern was defined as consuming vegetables and fruits daily and red meat 1 to 6 days per week, aligning with current recommendations that emphasize increased vegetable and fruit intake while limiting red meat consumption^18^. The total healthy lifestyle behavior score^66^ was the sum of each lifestyle score, ranging from 0 to 6, with a higher score indicating healthier lifestyle.

Regarding the binary approach to assess dietary pattern, we also calculated the dietary approach to stop hypertension (DASH) scores referring to American Heart Association guidelines on lifestyle management ^72^.

### Measurement of dyslipidemia

Fasting blood samples for all participants were collected from the study subjects. According to the 2023 Chinese Guidelines for Lipid Management^2^, dyslipidemia was defined as one or more abnormities of (1) elevated total cholesterol (TC) ≥ 6.2 mmol/L; (2) elevated low-density lipoprotein cholesterol (LDL-C) ≥ 4.1 mmol/L; (3) decreased high-density lipoprotein cholesterol (HDL-C) < 1.0 mmol/L; and (4) elevated triglycerides (TG) ≥ 2.3 mmol/L.

### Stool sampling and DNA extraction

Stool samples were freshly collected in a pre-distributed PP material stool samplers at defecation on the day of the survey. Stool samples were stored at −80 °C until DNA extraction through cold chain transportation within 2 h. Genomic DNA was extracted using hexadecyl trimethyl ammonium bromide (CTAB). DNA concentration and purity were measured on a 1% polyagarose gel. Qubit® dsDNA detection kit in the 2.0 fluorometer (Life Technologies, Carlsbad, CA, USA) was used to measure DNA concentration.

### DNA library construction and shotgun metagenomic sequencing

Sequencing libraries were generated using the NEBNext^®^ Ultra^TM^ DNA Library Prep Kit (Illumina, NEB, USA). DNA was randomly fragmented to 350 bp using a Covaris M220 sonicator (Covaris, USA). Fragments were amplified and purified by polymerase chain reaction (PCR). Library quality was assessed on a Qubit 2.0 fluorometer (Thermo Fisher Scientific Inc., Waltham, MA, USA) and an Agilent Bioanalyzer 2100 system (Agilent Technologies, Palo Alto, CA, USA).

Shotgun metagenomic sequencing was performed at Novogene Bioinformatics Technology, Beijing, on an Illumina NovaSeq 6000 platform to generate 150 bp paired-end reads. In total, 11.12 ± 1.06 Gb of raw reads and 11.05 ± 1.07 Gb of clean reads per sample were generated. KneadData (https://github.com/biobakery/kneaddata) was used to trim the reads and remove Illumina adapters and low-quality readouts with Trimmomatic (v0.39)^73^. Following trimming, the KneadData integrated Bowtie2 tool (v2.4.5) was used to remove reads that aligned to the human genome (GRCh38/hg38)^74^. The taxonomic composition of the metagenome was profiled at all levels (phylum, class, family, genus, species) using Kraken 2 with the RefSeq database (release 212)^75^. The metagenome of each sample was reassembled into larger genomic fragments (alleles) using MEGAHIT (v.1.2.9)^76^. After metagenomic sequencing, a total of 11,119 species were identified.

### Fecal metabolic profiling

In this study, metabolomic analysis was performed on 297 stool samples randomly selected from the 1049 samples of CMEC, based on liquid chromatography-mass spectrometry (LC-MS). The quality control (QC) sample was prepared by mixing an equal aliquot of the supernatants from all samples^77^. LC-MS/MS non-targeted metabolomics profiling analyses were adopted using ACQUITY UHPLC system (Waters Corporation, Milford, USA) coupled with LTQ Orbitrap XL (Thermo Fisher Scientific, USA)^78,79^. The raw mass spectra downcomer files were converted to mzXML format using the MSConvert tool (Proteowizard package, v3.0.8789)^78^. Peak detection, filtering, and alignment were performed using the “XCMS” package in R^79^ to obtain a list of quantitative substances. Public databases such as the Human Metabolome Database (HMDB)^80^, LipidMaps^81^, the Kyoto Encyclopedia of Genes and Genomes (KEGG)^82^, and self-constructed substance libraries were used to identify metabolites (< 30 ppm).

### Covariates

According to previous studies^83,84^, we included a variety of covariates, including demographic characteristics and medical history, which were collected by trained investigators through face-to-face interview. Demographic characteristics included sex (male, female), age (year), ethnicity (Han, minority) and educational level (illiteracy, primary or school, college or above). Medical history included self-reported diseases and self-reported medication history.

### Statistical analyses

The statistical analyses were conducted in R software version 4.2.2. Categorical variables were reported as numbers and proportions and were compared with the Chi-square test (*chisq.test*). Continuous variables were described as the mean ± SD and were assessed for normality using the Shapiro-Wilk test (*shapiro.test*). For normally distributed data, we used the Student’s *t* test (*t.test*); for non-normally distributed data, we applied the Wilcoxon signed-rank test (*wilcox.test*). The association between lifestyle behavior score and dyslipidemia was analyzed by logistic models adjusted for covariates, using the *glm* function in R. Statistical significance was defined as *P* < 0.05.

### Analysis of the differential species related to dyslipidemia

After excluding viruses, protozoa, and fungi, we focused on the species with a minimum mean relative abundance of 0.01% in at least 10% of samples^85,86^. We calculated α diversity measured by Simpson, Shannon, and Pielou entropy (*diversity* in the *vegan* package). Rarefaction curves were generated to assess species richness and estimate the sequencing depth (*rarecurve* in *vegan*). Beta diversity was assessed using Principal Coordinate Analysis (PCoA) with Bray–Curtis distances (*vegdist* in *vegan*). PERMANOVA (*adonis2* in *vegan*) was used to test for group differences. *P* < 0.05 was considered statistically significant.

Differential species and function were identified using Microbiome Multivariable Associations with Linear Models (Maaslin) employing the R package *Maaslin2*^87^. The multiple comparisons were corrected by controlling the false discovery rate (FDR) using the Benjamini–Hochberg method. We set *q* < 0.25 as the significance threshold ^88,89^.

To better capture the complexity of microbial interactions, we have incorporated a network analysis of differential species using a Python-based SparCC tool^90^. Significant co-abundance was controlled at FDR < 0.05 using 100× permutation. The microbial species co-abundance networks were created by Gephi.

### Mediation analysis

Mediation analyses were performed using the *mediate* function in the R package *mediation*, adjusted for sex, age, and ethnicity. The mediation effect of differential microbial species in the associations between lifestyle behavior score and dyslipidemia was identified using mediation analyses. The mediated proportion was determined by dividing the mediated effect by the total effect. Statistical significance was defined as *P* < 0.05.

### Analysis of the dyslipidemia-related metabolites

R package *MetaboAnalystR* was used to analyze the differential metabolites between the participants with and without dyslipidemia. Dyslipidemia-related metabolites were screened by orthogonal partial least squares-discriminant analysis and volcano maps, with VIP > 1, log2(FC) > 2, and *P* < 0.05.

Spearman’s correlation analysis (*cor.test*) was used to determine the associations between dyslipidemia-related metabolites and the mediating species. The biochemical pathways of differential metabolites were identified using the KEGG database and then classified based on their involvement in these pathways. To demonstrate as more relevant correlations, absolute correlation coefficient >0.25 was shown in network plot.

### Sensitivity analysis

To evaluate the robustness of the findings, we performed two sensitivity analyses in discovery cohort with the following strategies: (1) excluding those who were ethnic minorities, since the minorities have different lifestyles or gut microbiome with the Han people^21,91^, and (2) excluding those with hyperlipidemia medications that may affect the gut microbiota.

### External validation

In the validation cohort, we replicated the associations between potential mediating species identified in the discovery cohort and hyperlipidemia risk. Microbiome analysis, metabolomics analysis, and Spearman’s correlation were as described for the discovery cohort.

## Supplementary information

Supplementary Material

## Data Availability

The raw microbiome sequencing data of CMEC used in this study have been deposited into the CNGB Sequence Archive (CNSA) (https://db.cngb.org/cnsa) of China National GeneBank DataBase (CNGBdb), with accession number CNP0004236 (Metagenome data of CMEC) and CNP0005937 (Metagenome data of HLPC).

## Code availability

The code used in this study for developing the model is provided at https://github.com/LijunZeng-2024/Dyslipidemia.
