# Supplementary material for: Mediation effect and metabolic pathways of gut microbiota in the associations between lifestyles and dyslipidemia
Source: NPJ Biofilms Microbiomes. 2025 May 28;11:90. doi: 10.1038/s41522-025-00721-x (PMC12120021; doi:10.1038/s41522-025-00721-x)
Supplement: Supplementary file 1 — Supplementary Material [file 41522_2025_721_MOESM1_ESM.pdf]

Supplementary Figure 1 Top abundant species

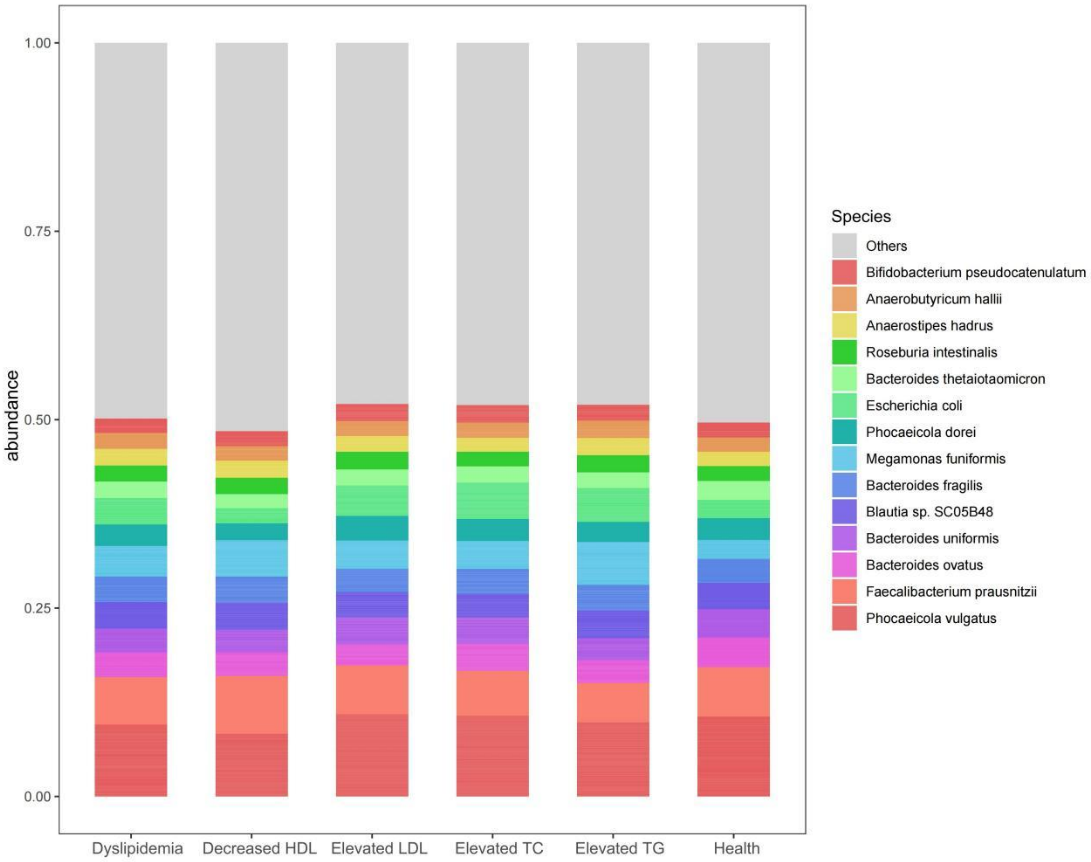

Supplementary Figure 2 Rarefaction curves

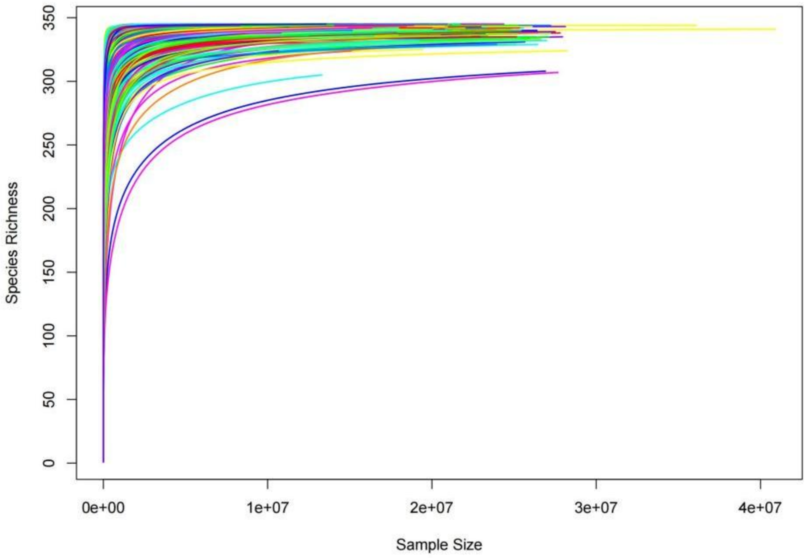

Supplementary Figure 3 Alpha and beta diversity in subgroups

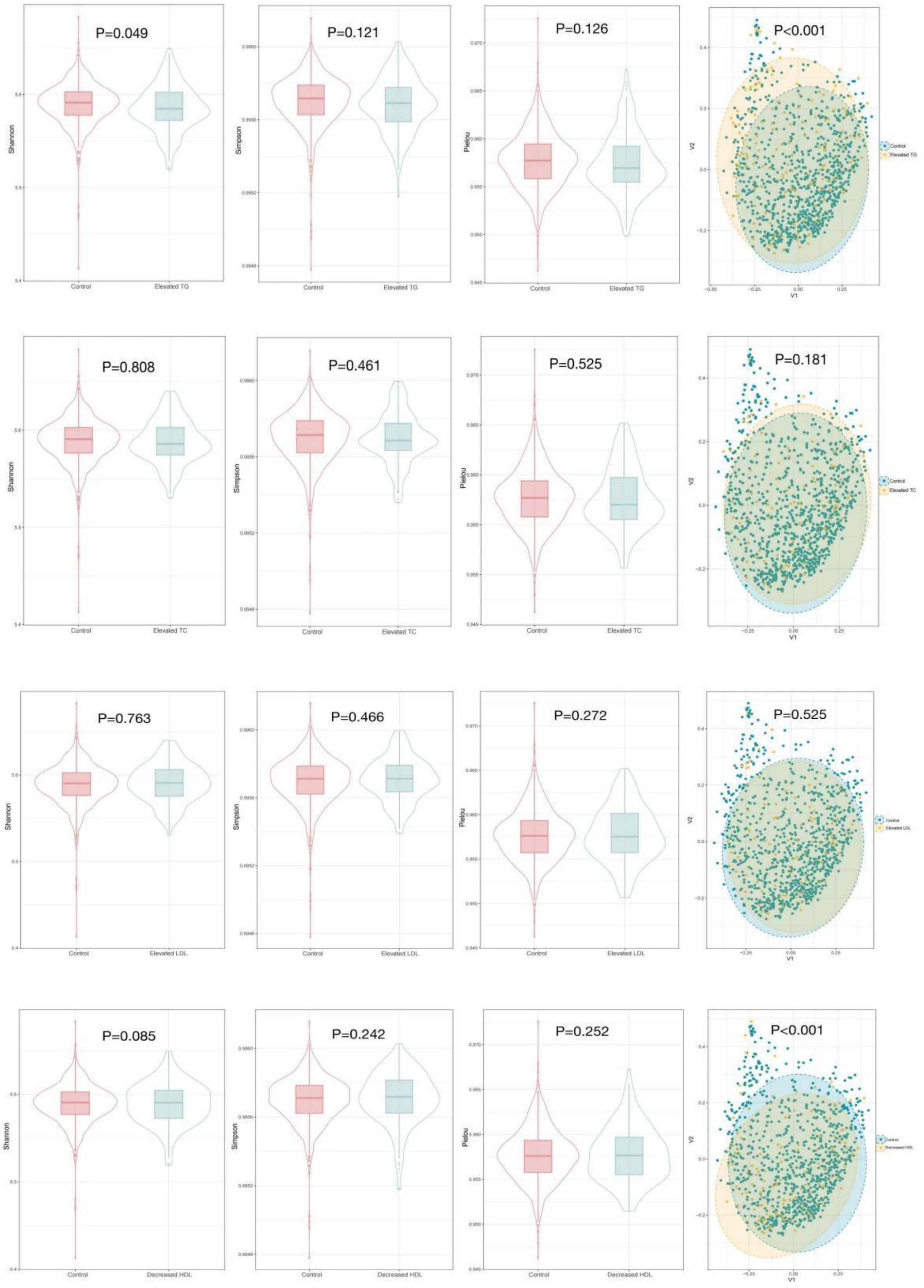

Supplementary Figure 4 Microbial species co-abundance networks

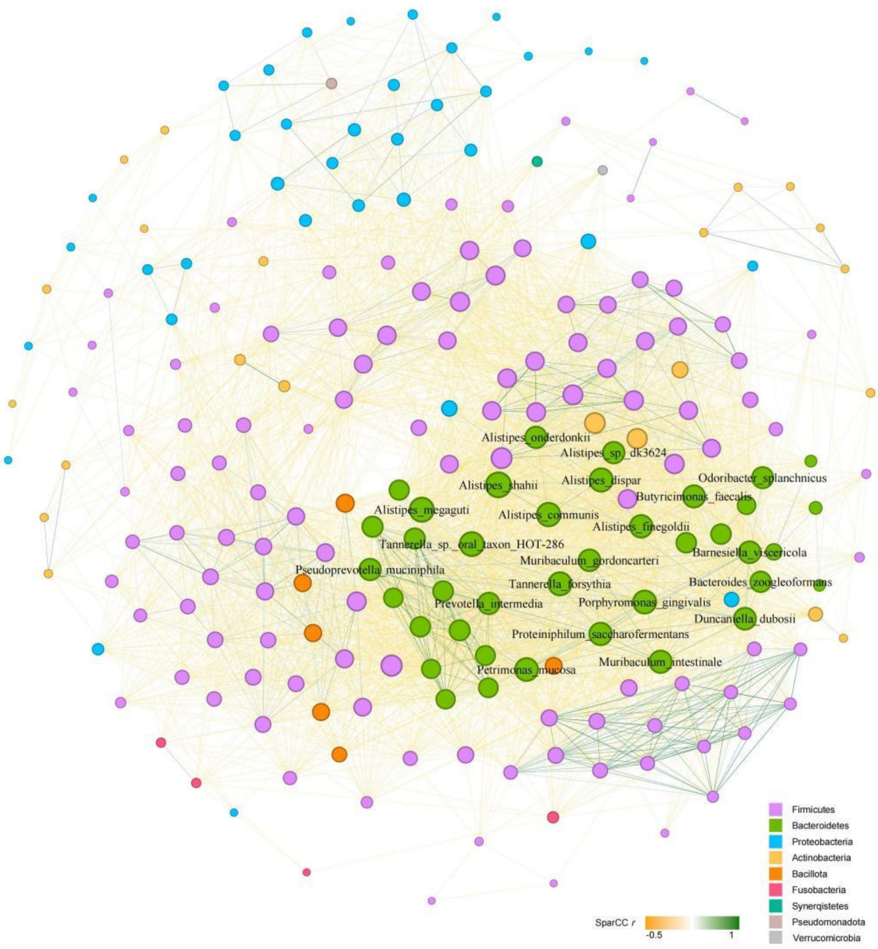

Only showed the edges with |r | > 0.30 to simplify the figure. The size of the nodes represents the level of degrees, and the thickness of the edges represents the absolute value of r.

Supplementary Figure 5 Heatmap of spearman's correlation between microbial function and lifestyle

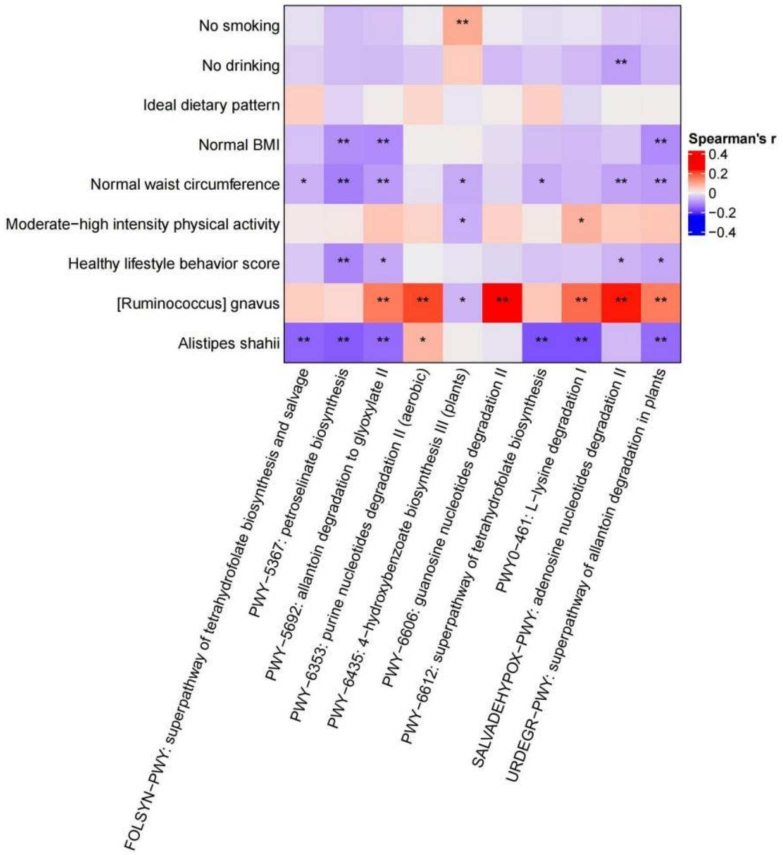

*: P < 0.05; **: P < 0.01.

Supplementary Figure 6 Differential metabolites and enrichment analysis in subgroups

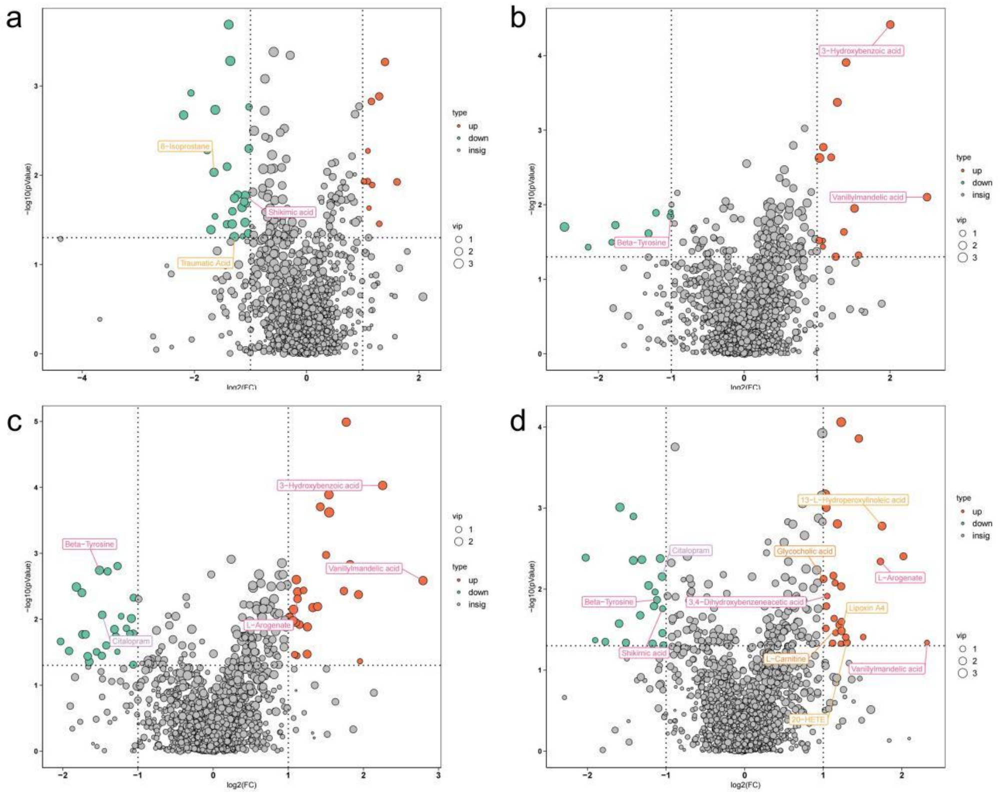

Differential metabolites in subgroup of decreased HDL (a), elevated LDL (b), elevated TC (c), and elevated TG (d).

Supplementary Figure 7 Species accumulation curve, alpha and beta diversity in validation cohort

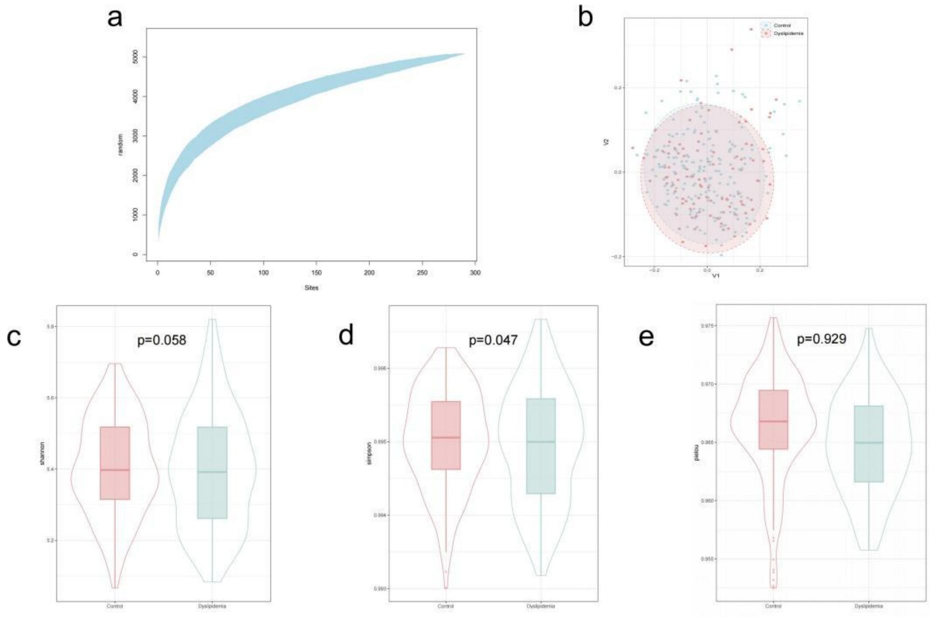

a. Species accumulation curve based on total numbers of species. b. PCoA plot shows that dyslipidemia and healthy people have essentially different gut microbiome profiles. c-e. Alpha diversity assessed by shannon, simpson, and pielou index.

Supplementary Table 1 Differential gut microbiota between dyslipidemia and control group
Supplementary Table 2 Differential gut microbiota between decreased HDL and control group
Supplementary Table 3 Differential gut microbiota between elevated LDL and control group
Supplementary Table 4 Differential gut microbiota between elevated TC and control group
Supplementary Table 5 Differential gut microbiota between elevated TG and control group
Supplementary Table 6 Species co-abundance network and keystone species
Supplementary Table 7 Microbial functions analysis
Supplementary Table 8 Mediating effect of gut microbiota between HLBS and dyslipidemia
Supplementary Table 9 Mediating effect of gut microbiota between HLBS and decreased HDL
Supplementary Table 10 Mediating effect of gut microbiota between HLBS and elevated LDL
Supplementary Table 11 Mediating effect of gut microbiota between HLBS and elevated TC
Supplementary Table 12 Mediating effect of gut microbiota between HLBS and elevated TG
Supplementary Table 13 Differential metabolites between dyslipidemia and control group
Supplementary Table 14 Differential metabolites between decreased HDL and control group
Supplementary Table 15 Differential metabolites between elevated LDL and control group
Supplementary Table 16 Differential metabolites between elevated TC and control group
Supplementary Table 17 Differential metabolites between elevated TG and control group
Supplementary Table 18 Sensitivity analysis (exclusion of who received anti-hyperlipidemia therapy): Differential gut microbiota between dyslipidemia and control group
Supplementary Table 19 Sensitivity analysis (exclusion of who received anti-hyperlipidemia therapy): Differential gut microbiota between decreased HDL and control group
Supplementary Table 20 Sensitivity analysis (exclusion of who received anti-hyperlipidemia therapy): Differential gut microbiota between elevated LDL and control group
Supplementary Table 21 Sensitivity analysis (exclusion of who received anti-hyperlipidemia therapy): Differential gut microbiota between elevated TC and control group
Supplementary Table 22 Sensitivity analysis (exclusion of who received anti-hyperlipidemia therapy): Differential gut microbiota between elevated TG and control group
Supplementary Table 23 Sensitivity analysis (exclusion of who received anti-hyperlipidemia therapy): Mediating effect of gut microbiota between HLBS and dyslipidemia
Supplementary Table 24 Sensitivity analysis (exclusion of who received anti-hyperlipidemia therapy): Mediating effect of gut microbiota between HLBS and decreased HDL
Supplementary Table 25 Sensitivity analysis (exclusion of who received anti-hyperlipidemia therapy): Mediating effect of gut microbiota between HLBS and elevated LDL
Supplementary Table 26 Sensitivity analysis (exclusion of who received anti-hyperlipidemia therapy): Mediating effect of gut microbiota between HLBS and elevated TC
Supplementary Table 27 Sensitivity analysis (exclusion of who received anti-hyperlipidemia therapy): Mediating effect of gut microbiota between HLBS and elevated TG
Supplementary Table 28 Sensitivity analysis (exclusion of who received anti-hyperlipidemia therapy): Differential metabolites between dyslipidemia and control group
Supplementary Table 29 Sensitivity analysis (exclusion of who received anti-hyperlipidemia therapy): Differential metabolites between decreased HDL and control group
Supplementary Table 30 Sensitivity analysis (exclusion of who received anti-hyperlipidemia therapy): Differential metabolites between elevated LDL and control group
Supplementary Table 31 Sensitivity analysis (exclusion of who received anti-hyperlipidemia therapy): Differential metabolites between elevated TC and control group
Supplementary Table 32 Sensitivity analysis (exclusion of who received anti-hyperlipidemia therapy): Differential metabolites between elevated TG and control group
Supplementary Table 33 Sensitivity analysis (exclusion of other ethnicity): Differential gut microbiota between dyslipidemia and control group
Supplementary Table 34 Sensitivity analysis (exclusion of other ethnicity): Differential gut microbiota between decreased HDL and control group
Supplementary Table 35 Sensitivity analysis (exclusion of other ethnicity): Differential gut microbiota between elevated TC and control group
Supplementary Table 36 Sensitivity analysis (exclusion of other ethnicity): Differential gut microbiota between elevated TG and control group
Supplementary Table 37 Sensitivity analysis (exclusion of other ethnicity): Mediating effect of gut microbiota between HLBS and dyslipidemia
Supplementary Table 38 Sensitivity analysis (exclusion of other ethnicity): Mediating effect of gut microbiota between HLBS and decreased HDL
Supplementary Table 39 Sensitivity analysis (exclusion of other ethnicity): Mediating effect of gut microbiota between HLBS and elevated TC
Supplementary Table 40 Sensitivity analysis (exclusion of other ethnicity): Mediating effect of gut microbiota between HLBS and elevated TG
Supplementary Table 41 Sensitivity analysis (exclusion of other ethnicity): Differential metabolites between dyslipidemia and control group
Supplementary Table 42 Sensitivity analysis (exclusion of other ethnicity): Differential metabolites between decreased HDL and control group
Supplementary Table 43 Sensitivity analysis (exclusion of other ethnicity): Differential metabolites between elevated LDL and control group
Supplementary Table 44 Sensitivity analysis (exclusion of other ethnicity): Differential metabolites between elevated TC and control group
Supplementary Table 45 Sensitivity analysis (exclusion of other ethnicity): Differential metabolites between elevated TG and control group
Supplementary Table 46 Differential gut microbiota between dyslipidemia and control group in validation cohort
Supplementary Table 47 Differential gut microbiota between decreased HDL and control group in validation cohort
Supplementary Table 48 Differential gut microbiota between elevated LDL and control group in validation cohort
Supplementary Table 49 Differential gut microbiota between elevated TC and control group in validation cohort
Supplementary Table 50 Differential gut microbiota between elevated TG and control group in validation cohort
Supplementary Table 51 Differential metabolites between dyslipidemia and control group in validation cohort
Supplementary Table 52 Differential metabolites between decreased HDL and control group in validation cohort
Supplementary Table 53 Differential metabolites between elevated LDL and control group in validation cohort
Supplementary Table 54 Differential metabolites between elevated TC and control group in validation cohort
Supplementary Table 55 Differential metabolites between elevated TG and control group in validation cohort

Supplementary Table 1 Differential gut microbiota between dyslipidemia and control group

Species	coef	stderr	p value	q value
[Ruminococcus] gnavus	0.27818289	0.060157944	4.26E-06	0.001469781
Lachnospiraceae bacterium	0.152949086	0.041730999	0.000260325	0.04490611
Aeromonas veronii	-0.158549102	0.044914009	0.000436461	0.050193044
Lachnospira eligens	-0.187117093	0.058395353	0.001397184	0.108218609
Ligilactobacillus ruminis	0.25165165	0.079371799	0.001568386	0.108218609
Bifidobacterium catenulatum	0.315129692	0.101672829	0.001998939	0.114939002
Lachnospiraceae bacterium Choco86	0.111940384	0.036789258	0.002422251	0.119382372
Haemophilus parainfluenzae	-0.315378426	0.107062319	0.003339576	0.119901087
Blautia argi	0.125035175	0.04266258	0.003475394	0.119901087
Streptococcus mitis	0.169129326	0.056679659	0.002915555	0.119901087
Finegoldia magna	0.103670317	0.036410457	0.004507527	0.141372439
[Clostridium] hylemonae	0.095036871	0.034465383	0.005932735	0.16132563
Fusobacterium varium	0.205066158	0.074585723	0.006078937	0.16132563
Ruminococcus bicirculans	-0.221933601	0.084269172	0.008579284	0.164436274
Streptococcus sp. LPB0220	0.209775045	0.079314832	0.008395949	0.164436274
Streptococcus sp. HSISM1	0.204665671	0.07750067	0.008487877	0.164436274
Streptococcus parasanguinis	0.199692455	0.07581317	0.008569755	0.164436274
Amedibacterium intestinale	0.163544323	0.061167224	0.007625578	0.164436274
Streptococcus vestibularis	0.202036967	0.077459304	0.009291307	0.168710572
Brevundimonas naejangsanensis	-0.201461111	0.079437177	0.011388475	0.184327304
Lachnospiraceae bacterium KGMB03038	0.084097484	0.033526863	0.012288487	0.184327304
[Clostridium] scindens	0.092340871	0.036428562	0.011403082	0.184327304
Streptococcus pneumoniae	0.105182814	0.041754407	0.011926216	0.184327304
Faecalitalea cylindroides	0.104537732	0.042441791	0.013984749	0.185566859
Megamonas hypermegale	0.181338524	0.073601065	0.013915911	0.185566859
Bifidobacterium breve	0.157211448	0.06354889	0.013532843	0.185566859
Escherichia marmotae	0.175259802	0.071934088	0.01501265	0.191828307
Bacteroides intestinalis	-0.195278736	0.081603569	0.016896786	0.20819254
Eubacterium limosum	0.105384931	0.04445906	0.01800389	0.214184211
Psychrobacter sp. P11G5	-0.22700143	0.097312416	0.01986324	0.228427263
Escherichia coli	0.234278275	0.104208149	0.024798771	0.244075754
Lachnoclostridium phocaeense	0.073667628	0.032679902	0.024400598	0.244075754
Anaerotignum propionicum	0.073901548	0.033135349	0.025961297	0.244075754
Ruminococcus albus	-0.098050915	0.043482038	0.024352238	0.244075754
Lactococcus cremoris	0.101319536	0.045497549	0.02617624	0.244075754
Megamonas funiformis	0.344205503	0.152158021	0.023903311	0.244075754
Bifidobacterium pseudocatenulatum	0.31528843	0.138409372	0.022943983	0.244075754
Sporosarcina psychrophila	-0.154271541	0.069632932	0.026952604	0.244701276

Supplementary Table 2 Differential gut microbiota between decreased HDL and control group

Species	coef	stderr	p value	q value
Olsenella sp. GAM18	0.533386261	0.17466459	0.002414356	0.033059184
Phocaeicola vulgatus	-0.581040383	0.256447604	0.023744249	0.160622861
Bacteroides uniformis	-0.559918615	0.269768662	0.038267451	0.21293985
Phocaeicola dorei	-0.47435771	0.229683357	0.039263315	0.215013391
Bacteroides caecimuris	-0.43968526	0.213628616	0.039918959	0.21518814
Bacteroides intestinalis	-0.47217125	0.235139251	0.044989256	0.235171109
Bifidobacterium catenulatum	1.841845358	0.287613903	3.31E-10	5.72E-08
Bifidobacterium adolescentis	1.675514989	0.330386714	4.95E-07	4.60E-05
Bifidobacterium angulatum	1.127961602	0.223068223	5.34E-07	4.60E-05
Bifidobacterium bifidum	1.329002891	0.266865251	7.85E-07	5.41E-05
Bifidobacterium pseudocatenulatum	1.431636011	0.387145481	0.000233855	0.008964447
Bifidobacterium breve	0.538822634	0.181112667	0.003020594	0.035934653
Bifidobacterium longum	0.822164715	0.284718365	0.004079137	0.044787832
Christensenella sp. Marseille-P3954	0.358970913	0.120021489	0.002870198	0.035364943
Christensenella minuta	0.29441889	0.139884966	0.035638216	0.204919742
Clostridium sp. SY8519	0.27752643	0.109919663	0.011780568	0.09451851
Collinsella aerofaciens	0.694733716	0.273546267	0.011289169	0.092732462
Enterobacter roggenkampii	-1.315854915	0.301866226	1.55E-05	0.000891453
Raoultella planticola	-0.903752842	0.247488701	0.000291388	0.010052877
Klebsiella pneumoniae	-1.075029764	0.29775606	0.000325462	0.01020768
Klebsiella variicola	-1.029847229	0.309977923	0.000935406	0.018983241
Raoultella ornithinolytica	-1.010850391	0.302598886	0.000890112	0.018983241
Klebsiella quasipneumoniae	-0.851298034	0.260541619	0.00113476	0.021569818
Klebsiella aerogenes	-0.630903579	0.204753618	0.002135314	0.032029715
Enterobacter kobei	-0.815567924	0.266048056	0.002250895	0.032356613
Klebsiella sp. FDAARGOS_511	-0.70239935	0.233347976	0.002702981	0.034538092
Klebsiella oxytoca	-0.567768793	0.199363028	0.004523795	0.047294216
Enterobacter cloacae	-0.512587921	0.207471211	0.013703015	0.107444098
Enterobacter bugandensis	-0.564472014	0.252074905	0.025423347	0.165491596
Enterococcus casseliflavus	-0.676603964	0.265072309	0.010885887	0.091600755
Enterococcus avium	-0.566967766	0.232939038	0.015160049	0.113700371
Faecalitalea cylindroides	0.464854887	0.12475225	0.000213154	0.008964447
Intestinibaculum porci	0.388124377	0.11108576	0.000503133	0.014344104
Lachnospira eligens	-0.534268634	0.164776344	0.001237426	0.021569818
Lachnospiraceae bacterium Choco86	0.325083046	0.107081658	0.002491417	0.033059184
Lachnospiraceae bacterium KM106-2	0.25536674	0.109389521	0.019838419	0.142372895
Ligilactobacillus ruminis	1.613889745	0.234666997	1.26E-11	4.35E-09
Leuconostoc mesenteroides	0.476246799	0.162629206	0.003544307	0.040759526
Fructilactobacillus sanfranciscensis	0.460786175	0.160284805	0.004154234	0.044787832
Limosilactobacillus mucosae	0.624273054	0.2986423	0.036911459	0.208761531
Lactobacillus delbrueckii	0.256901582	0.127933535	0.044982033	0.235171109
Acinetobacter johnsonii	-0.436376049	0.201744307	0.030848277	0.18038399
Psychrobacter sp. P11F6	-0.556302038	0.277979413	0.045745437	0.235554862
Oscillibacter valericigenes	0.592460835	0.177954277	0.000912003	0.018983241
Ethanoligenens harbinense	0.418126085	0.130168259	0.001371965	0.022539431
Dysosmobacter welbionis	0.397296056	0.141612338	0.005149964	0.052256987
Caproiciproducens sp. NJN-50	0.298693927	0.114022746	0.008976253	0.078113009
Oscillibacter sp. PEA192	0.434420089	0.179471679	0.015727124	0.11544378
Ruminococcus champanellensis	0.361086749	0.166132798	0.030047303	0.180014638
Haemophilus parainfluenzae	-0.978340171	0.30203977	0.001250424	0.021569818
Prevotella fusca	0.586847188	0.25121304	0.020221078	0.142372895
Prevotella melaninogenica	0.566036619	0.248775631	0.023166787	0.159850829
Prevotella ruminicola	0.589232082	0.260578258	0.024302245	0.161236052

Species	coef	stderr	p value	q value
Prevotella dentalis	0.556984367	0.255960673	0.02985429	0.180014638
Prevotella scopos	0.512426661	0.236071271	0.03026333	0.180014638
Lactococcus cremoris	0.545887311	0.127742496	2.17E-05	0.001067708
Streptococcus koreensis	-0.741249824	0.213251441	0.000540502	0.014344104
Streptococcus ilei	-0.733529257	0.212760672	0.000600635	0.014801371
Lactococcus lactis	0.442274328	0.16226499	0.006563279	0.061198143
Streptococcus viridans	-0.468523853	0.1908512	0.014332927	0.109885774
Cloacibacillus porcorum	0.377421228	0.172549921	0.029019169	0.180014638
Intestinimonas butyriciproducens	0.479701328	0.175575806	0.006435825	0.061198143
Clostridiales bacterium CCNA10	0.34868152	0.131535896	0.008193778	0.074390883
Flintibacter sp. KGMB00164	0.350167561	0.133828773	0.009056581	0.078113009
Dialister hominis	1.110802078	0.35992318	0.002099608	0.032029715
Dialister massiliensis	0.982764754	0.35216805	0.005390644	0.05313635
Yersinia enterocolitica	-0.33048067	0.150894465	0.02885909	0.180014638

Supplementary Table 3 Differential gut microbiota between elevated LDL and control group

Species	coef	stderr	p value	q value
[Ruminococcus] gnavus	0.701050803	0.188102931	0.00020889	0.072066906

Supplementary Table 4 Differential gut microbiota between elevated TC and control group

Species	coef	stderr	p value	q value
[Ruminococcus] gnavus	0.790310354	0.191931717	4.27E-05	0.014733205
Streptococcus pneumoniae	0.44483052	0.134481487	0.000987185	0.095057994
Streptococcus mitis	0.625168109	0.184599385	0.000745987	0.095057994
Streptococcus cristatus	0.526037877	0.160558068	0.001102122	0.095057994
Escherichia coli	1.028322065	0.341989333	0.002731325	0.12728087
Escherichia marmotae	0.690573804	0.237534114	0.003758655	0.12728087
Citrobacter tructae	0.809065694	0.280609199	0.004058231	0.12728087
Streptococcus oralis	0.534332749	0.179781536	0.003055971	0.12728087
Megamonas funiformis	1.458972086	0.50292562	0.003833392	0.12728087
Fusobacterium varium	0.70504232	0.238885419	0.003266292	0.12728087
Fusobacterium mortiferum	1.035429418	0.342179573	0.002567467	0.12728087
Escherichia fergusonii	0.696386724	0.260080913	0.007584417	0.218051976
Escherichia albertii	0.646819936	0.245896837	0.008709563	0.231138415

Supplementary Table 5 Differential gut microbiota between elevated TG and control group

Species	coef	stderr	p value	q value
[Ruminococcus] gnavus	0.998962281	0.15697452	3.33E-10	1.15E-07
Lachnospiraceae bacterium	0.566203903	0.110747868	3.97E-07	5.07E-05
Blautia argi	0.574179081	0.112754818	4.41E-07	5.07E-05
Streptococcus mitis	0.720549573	0.14559296	9.12E-07	6.99E-05
Amedibacterium intestinale	0.796099138	0.161548423	1.01E-06	6.99E-05
Absiella argi	0.568919366	0.122090271	3.70E-06	0.000212854
Fusobacterium varium	0.922532557	0.199377755	4.32E-06	0.000213117
Streptococcus parasanguinis	0.906254805	0.198618478	5.84E-06	0.000251795
Streptococcus vestibularis	0.895196655	0.201703436	1.14E-05	0.000437986
Brevundimonas naejangsanensis	-0.912274862	0.213356335	2.39E-05	0.000764923
Streptococcus cristatus	0.540877285	0.127409363	2.44E-05	0.000764923
Streptococcus sp. LPB0220	0.886099167	0.209946582	2.86E-05	0.000765956
Streptococcus pneumoniae	0.450343352	0.10702814	2.89E-05	0.000765956
Streptococcus sp. HSISM1	0.856326746	0.205800588	3.67E-05	0.000903648
Aeromonas veronii	-0.497085474	0.123432894	6.29E-05	0.00144635
Odoribacter splanchnicus	-0.869352753	0.225215341	0.000122903	0.002231666
Porphyromonas asaccharolytica	-0.789245518	0.203862218	0.000120107	0.002231666
Oscillibacter valericigenes	-0.606427922	0.157140617	0.000122889	0.002231666
Ethanoligenens harbinense	-0.464153492	0.119861696	0.00011654	0.002231666
Clostridioides difficile	0.296375967	0.078794615	0.000181267	0.002977966
Streptococcus salivarius	0.813604537	0.215513804	0.000172677	0.002977966
Alistipes communis	-0.891260631	0.238336499	0.000197445	0.002981174
Schaalia odontolytica	0.558847563	0.149501274	0.000198745	0.002981174
Escherichia marmotae	0.696083697	0.189377296	0.000253514	0.003644268
Escherichia coli	0.982888021	0.273806268	0.000352247	0.004742486
[Clostridium] scindens	0.347187138	0.097635205	0.000398644	0.004742486
[Eubacterium] sulci	0.369023044	0.103403651	0.000379987	0.004742486
Streptococcus pasteurianus	0.572900133	0.160087395	0.000384182	0.004742486
Gemella sanguinis	0.517431072	0.145268997	0.000390827	0.004742486
Streptococcus sp. FDAARGOS_192	0.779944164	0.219919176	0.000414108	0.004762243
Muribaculum gordoncarteri	-0.631132362	0.179842509	0.000501486	0.005581059
Alistipes shahii	-0.950369507	0.273545883	0.00053964	0.00564169
Megamonas hypermegale	0.677401354	0.194513687	0.000523434	0.00564169
Faecalibacterium prausnitzii	-0.604067011	0.175401198	0.00060339	0.006122631
Butyricimonas faecalis	-0.705715587	0.209070432	0.000779439	0.007683042
Muribaculum intestinale	-0.573622726	0.170591542	0.000808673	0.007749785
Ruminococcus albus	-0.381926285	0.11635363	0.001073372	0.010008472
Anaerostipes rhamnosivorans	0.313804611	0.095924135	0.001166148	0.010468469
Fusobacterium mortiferum	0.890542035	0.273629049	0.001183392	0.010468469
Alistipes megaguti	-0.798049151	0.245967447	0.001225409	0.01056915
Barnesiella viscericola	-0.643419815	0.201079202	0.001430646	0.011433825
Lachnospiraceae bacterium Choco86	0.314077263	0.098448711	0.001491368	0.011433825
[Clostridium] hylemonae	0.296377285	0.092853555	0.001468547	0.011433825
Lacrimispora sphenoides	0.303299563	0.095109346	0.001483196	0.011433825
Streptococcus sp. oral taxon 431	0.489223951	0.153151516	0.001465277	0.011433825
Mogibacterium diversum	0.37964794	0.119486675	0.001547008	0.011602558
Alistipes onderdonkii	-0.894877055	0.283012139	0.001625977	0.011709373
Brevundimonas diminuta	-0.702017326	0.221430015	0.00162913	0.011709373
Oscillibacter sp. PEA192	-0.519668963	0.165704359	0.001774779	0.012495891
Escherichia albertii	0.606341524	0.194661047	0.001908666	0.013169792
Clostridium beijerinckii	0.297452364	0.09572541	0.001982511	0.013411103
Porphyromonas gingivalis	-0.511481733	0.165330541	0.002080224	0.013541083
Blautia producta	0.316076103	0.102193311	0.002050654	0.013541083

Species	coef	stderr	p value	q value
Alistipes finegoldii	-0.694536403	0.226655042	0.0022548	0.013647472
Ruminococcus champanellensis	-0.473368025	0.154458607	0.002251566	0.013647472
Intestinimonas butyriciproducens	-0.49774956	0.161637498	0.002144435	0.013647472
Eubacterium limosum	0.364395758	0.118802997	0.002253947	0.013647472
[Clostridium] innocuum	0.417970895	0.136932316	0.002400413	0.014278316
Duncaniella dubosii	-0.499109939	0.165493653	0.002672375	0.015366157
Arabia massiliensis	-0.502940307	0.166705867	0.002634664	0.015366157
Eubacterium callanderi	0.36184257	0.123304642	0.003435263	0.019428945
Prevotella enoeca	-0.663896927	0.227456486	0.003611796	0.019542573
Escherichia fergusonii	0.606024848	0.20765205	0.003625289	0.019542573
Lachnospiraceae bacterium KGMB03038	0.262802914	0.090052338	0.003617472	0.019542573
Fusobacterium ulcerans	0.593560977	0.206058058	0.004077365	0.021641399
Streptococcus oralis	0.404681181	0.14117258	0.004261701	0.022277074
Lachnospira eligens	-0.442962817	0.155423509	0.004483524	0.023086804
Prevotella dentalis	-0.676852829	0.240982392	0.005094162	0.02584538
Ruminococcus bicirculans	-0.62747852	0.223848037	0.005182234	0.025911171
Lachnoclostridium phocaeense	0.238956792	0.087182667	0.006263926	0.030872207
Finegoldia magna	0.258749513	0.094617113	0.006386348	0.031032255
Alistipes sp. dk3624	-0.60282074	0.221497853	0.006638182	0.03167702
Anaerotignum propionicum	0.239606809	0.088131103	0.006702674	0.03167702
Bacteroides intestinalis	-0.57235121	0.216251804	0.008289201	0.038130326
Clostridium sp. DL-VIII	0.249494432	0.094135032	0.008210548	0.038130326
Tannerella forsythia	-0.452728809	0.171737717	0.008546836	0.038294266
Lachnoclostridium sp. YL32	0.28721392	0.108859061	0.00849076	0.038294266
Prevotella intermedia	-0.509945771	0.194132741	0.008783722	0.038359293
Hungatella hathewayi	0.267480278	0.101488443	0.008695679	0.038359293
Enterocloster bolteae	0.365600316	0.141336463	0.009917072	0.042239382
Lactobacillus johnsonii	0.285971066	0.110483561	0.009875952	0.042239382
Megamonas funiformis	1.031033682	0.401130713	0.010339018	0.043499525
Streptococcus australis	0.401876492	0.156686235	0.010502056	0.043653123
Petrimonas mucosa	-0.45122107	0.176503003	0.0107573	0.044028173
Clostridium perfringens	0.320842178	0.125581972	0.010847521	0.044028173
Fusobacterium nucleatum	0.256099667	0.10078753	0.011241863	0.045098173
Streptococcus equinus	0.408968912	0.161272659	0.011404166	0.045223418
Anaerocolumna sedimenticola	0.204741596	0.081385199	0.012157354	0.047662353
Psychrobacillus glaciei	-0.380430162	0.152632908	0.012886233	0.049952252
Comamonas kerstersii	-0.558253058	0.224216492	0.013042571	0.049996523
Alistipes dispar	-0.568879534	0.229520479	0.013395955	0.050786861
Streptococcus sanguinis	0.326621836	0.132368947	0.013812356	0.051796336
Lacrimispora saccharolytica	0.23935578	0.097167847	0.01397419	0.051839738
Pseudomonas taetrolens	-0.801880956	0.328349853	0.014816397	0.054379331
Sutterella faecalis	-0.529768069	0.218772855	0.015674502	0.056923192
Prevotella sp. oral taxon 299	-0.522993569	0.216733711	0.016041767	0.0576501
Streptococcus viridans	0.404300085	0.169474623	0.017280943	0.061463149
Peptacetobacter hiranonis	0.279364276	0.117577975	0.017760537	0.062524339
Arthrobacter sp. YC-RL1	-0.44668707	0.189152501	0.018437249	0.06425102
Campylobacter jejuni	0.307144262	0.131222711	0.019509779	0.067308737
Prevotella denticola	-0.534464914	0.229812132	0.02053526	0.069457498
Lachnoclostridium phytofermentans	0.196392048	0.08459257	0.020513491	0.069457498
Clostridium saccharoperbutylacetonicum	0.208119253	0.090443042	0.021711223	0.072521603
Clostridium bornimense	0.231379561	0.100846148	0.022032876	0.072521603
Adlercreutzia sp. 8CFCBH1	-0.657251167	0.286533475	0.022071792	0.072521603
Enterococcus faecium	0.417279314	0.183390742	0.023146305	0.07533467
Caproiciproducens sp. NJN-50	-0.237237438	0.10448628	0.023440337	0.075578658
Streptococcus ilei	0.413594405	0.185366365	0.025940431	0.082865267
Paraprevotella xylaniphila	-0.490377893	0.220898208	0.027024587	0.083995337

Species	coef	stderr	p value	q value
Clostridium baratii	0.285175348	0.128410785	0.026655658	0.083995337
Adlercreutzia equolifaciens	-0.767180017	0.34619928	0.02700292	0.083995337
Prevotella oris	-0.546018365	0.249093727	0.02888225	0.084792619
Pseudoprevotella muciniphila	-0.411228909	0.186737961	0.027935742	0.084792619
Alcaligenes faecalis	-0.285650661	0.130573719	0.029001533	0.084792619
Ruminococcus sp. JE7A12	-0.426727226	0.193895764	0.028036745	0.084792619
Streptococcus sp. A12	0.370657453	0.168228732	0.027857468	0.084792619
Sporosarcina psychrophila	-0.405498528	0.184927289	0.028611427	0.084792619
Oerskovia sp. KBS0722	-0.397554101	0.181415103	0.028725606	0.084792619
Anaerobutyricum hallii	0.293280514	0.134601322	0.0296384	0.085926454
Sutterella megalosphaeroides	-0.504196679	0.231930673	0.030002168	0.086256233
Lachnoanaerobaculum umeaense	0.178671754	0.082919434	0.031809793	0.090465095
Gordonibacter pamelaeae	-0.400299575	0.186318241	0.031990555	0.090465095
Tannerella sp. oral taxon HOT-286	-0.395009383	0.185632966	0.033650619	0.094385881
Streptococcus koreensis	0.397943626	0.187372358	0.033989856	0.094568551
Massilistercora timonensis	0.266032348	0.125841511	0.034829095	0.096128302
Clostridium botulinum	0.197143611	0.093821195	0.036019627	0.098363531
Bacillus sp. N3536	-0.499458152	0.238055292	0.036209184	0.098363531
Clostridium chauvoei	0.309084404	0.148985062	0.038365083	0.103405889
Bacteroides cellulosilyticus	-0.446663813	0.21614223	0.039098696	0.10456628
Clostridium pasteurianum	0.158595812	0.076979963	0.039699773	0.10535709
Flintibacter sp. KGMB00164	-0.255314135	0.124287037	0.040275973	0.106070311
Dialister massiliensis	-0.638674245	0.314103298	0.042346812	0.109846995
Actinomyces sp. oral taxon 414	0.296039069	0.145436863	0.042126833	0.109846995
Blautia sp. SC05B48	0.257854858	0.129435495	0.046741396	0.120341653
Proteiniphilum saccharofermentans	-0.339804762	0.173233718	0.050160602	0.125212471
Desulfovibrio piger	-0.422428102	0.215759012	0.050606633	0.125212471
Clostridium butyricum	0.218520856	0.111699954	0.050810858	0.125212471
Clostridium saccharobutylicum	0.208333851	0.105935214	0.049698446	0.125212471
Streptococcus dysgalactiae	0.199547738	0.101861521	0.050483358	0.125212471
Rhodococcus erythropolis	-0.450286191	0.229322003	0.049933075	0.125212471
Prevotella jejuni	-0.409690853	0.21049087	0.052076269	0.127420657
Prevotella ruminicola	-0.470416621	0.243113916	0.053344308	0.129604127
Bifidobacterium breve	0.316284872	0.164212432	0.054446872	0.131357838
Enterocloster clostridioformis	0.228696819	0.119414812	0.055828698	0.131923979
Clostridium septicum	0.287038844	0.149498499	0.055439464	0.131923979
Streptococcus gordonii	0.273882887	0.142840965	0.055542319	0.131923979
Gordonibacter urolithinfaciens	-0.323559404	0.170686639	0.058373241	0.136998424
Dysosmobacter welbionis	-0.245514009	0.130368108	0.060028389	0.139931042
Olsenella sp. GAM18	-0.298501632	0.159546	0.061714971	0.14289708
Anaerostipes hadrus	0.280896251	0.150672416	0.062678181	0.144159816
Bacteroides uniformis	-0.462951517	0.250527129	0.064982509	0.147498646
Streptococcus thermophilus	0.334909088	0.181238591	0.064984911	0.147498646
Prevotella fusca	-0.432539856	0.236516076	0.068013369	0.15336348
Lelliottia sp. WB101	-0.332113091	0.183563978	0.070849329	0.157727208
Psychrobacillus sp. AK 1817	-0.469194014	0.259402886	0.070862948	0.157727208
Prevotella melaninogenica	-0.415297294	0.232439609	0.074366064	0.163415874
Rothia mucilaginosa	0.289822348	0.161961071	0.073917331	0.163415874
Bacteroides xylanisolvens	-0.382510336	0.214640216	0.075178184	0.164154896
Pseudomonas stutzeri	-0.286405617	0.161478358	0.076500795	0.165992291
Pseudomonas versuta	-0.549438551	0.31283355	0.079413499	0.171235358
Clostridium isatidis	0.233218192	0.134234952	0.082789745	0.177406597
Carnobacterium maltaromaticum	0.208448864	0.120394004	0.083779415	0.177941149
Erysipelotrichaceae bacterium GAM147	0.281611853	0.16269433	0.084070746	0.177941149
Bacteroides zoogleoformans	-0.332305739	0.19282665	0.085210707	0.179254231
Psychrobacter sp. P11G5	-0.452264485	0.263169215	0.086085254	0.17999644

Species	coef	stderr	p value	q value
Akkermansia muciniphila	-0.438076216	0.260384516	0.092874703	0.193022726
Microbacterium oxydans	-0.379255599	0.226840076	0.095012641	0.1962836
Oxalobacter formigenes	-0.349316696	0.210191883	0.096936512	0.19906605
Alcaligenes aquatilis	-0.359382463	0.218304951	0.100116768	0.204380384
Bifidobacterium pseudocatenulatum	0.604582079	0.36961658	0.102307609	0.207624264
Clostridium taeniosporum	0.201139576	0.124517999	0.106631459	0.215133645
Clostridium novyi	0.155365378	0.098081845	0.113580849	0.227510677
Bifidobacterium dentium	-0.316304299	0.199712451	0.114085064	0.227510677
Streptococcus infantarius	0.298728922	0.189233431	0.114894161	0.227807387
Veillonella atypica	0.342106256	0.221765116	0.123772541	0.244008724

Supplementary Table 6 Species co-abundance network and keystone species

Species	phylum	class	order	family	genus	Node degree	closnesscentrality	betweenesscentrality
Bacteroides cellulosilyticus	Bacteroidetes	Bacteroidia	Bacteroidales	Bacteroidaceae	Bacteroides	86	0.537313	108.583922
Bacteroides xylanisolvens	Bacteroidetes	Bacteroidia	Bacteroidales	Bacteroidaceae	Bacteroides	32	0.444444	0.427024
Bacteroides intestinalis	Bacteroidetes	Bacteroidia	Bacteroidales	Bacteroidaceae	Bacteroides	74	0.509434	62.570837
Bacteroides caecimuris	Bacteroidetes	Bacteroidia	Bacteroidales	Bacteroidaceae	Bacteroides	61	0.493151	53.434853
Bacteroides zoogleoformans	Bacteroidetes	Bacteroidia	Bacteroidales	Bacteroidaceae	Bacteroides	91	0.548223	135.246558
Phocaeicola vulgatus	Bacteroidetes	Bacteroidia	Bacteroidales	Bacteroidaceae	Phocaeicola	31	0.440816	9.892674
Phocaeicola dorei	Bacteroidetes	Bacteroidia	Bacteroidales	Bacteroidaceae	Phocaeicola	39	0.449064	21.105601
Tannerella forsythia	Bacteroidetes	Bacteroidia	Bacteroidales	Tannerellaceae	Tannerella	107	0.595041	232.872827
Tannerella sp. oral taxon HOT-286	Bacteroidetes	Bacteroidia	Bacteroidales	Tannerellaceae	Tannerella	112	0.618911	340.188043
Paraprevotella xylaniphila	Bacteroidetes	Bacteroidia	Bacteroidales	Prevotellaceae	Paraprevotella	86	0.549618	145.059223
Prevotella intermedia	Bacteroidetes	Bacteroidia	Bacteroidales	Prevotellaceae	Prevotella	97	0.57754	151.848296
Prevotella ruminicola	Bacteroidetes	Bacteroidia	Bacteroidales	Prevotellaceae	Prevotella	78	0.546835	74.224494
Prevotella dentalis	Bacteroidetes	Bacteroidia	Bacteroidales	Prevotellaceae	Prevotella	90	0.566929	111.644367
Prevotella melaninogenica	Bacteroidetes	Bacteroidia	Bacteroidales	Prevotellaceae	Prevotella	79	0.549618	69.431511
Prevotella fusca	Bacteroidetes	Bacteroidia	Bacteroidales	Prevotellaceae	Prevotella	82	0.55243	169.162458
Prevotella denticola	Bacteroidetes	Bacteroidia	Bacteroidales	Prevotellaceae	Prevotella	90	0.566929	110.211207
Prevotella oris	Bacteroidetes	Bacteroidia	Bacteroidales	Prevotellaceae	Prevotella	85	0.55814	92.362802
Prevotella jejuni	Bacteroidetes	Bacteroidia	Bacteroidales	Prevotellaceae	Prevotella	81	0.55243	71.873688
Prevotella sp. oral taxon 299	Bacteroidetes	Bacteroidia	Bacteroidales	Prevotellaceae	Prevotella	87	0.561039	99.356147
Prevotella scopos	Bacteroidetes	Bacteroidia	Bacteroidales	Prevotellaceae	Prevotella	80	0.549618	67.688615
Prevotella enoeca	Bacteroidetes	Bacteroidia	Bacteroidales	Prevotellaceae	Prevotella	89	0.565445	106.245793
Pseudoprevotella muciniphila	Bacteroidetes	Bacteroidia	Bacteroidales	Prevotellaceae	Pseudoprevotella	97	0.57754	143.632866
Butyricimonas faecalis	Bacteroidetes	Bacteroidia	Bacteroidales	Odoribacteraceae	Butyricimonas	101	0.595041	184.527854
Odoribacter splanchnicus	Bacteroidetes	Bacteroidia	Bacteroidales	Odoribacteraceae	Odoribacter	95	0.579088	108.701628
Alistipes shahii	Bacteroidetes	Bacteroidia	Bacteroidales	Rikenellaceae	Alistipes	118	0.652568	753.853267
Alistipes megaguti	Bacteroidetes	Bacteroidia	Bacteroidales	Rikenellaceae	Alistipes	113	0.637168	502.683246
Alistipes communis	Bacteroidetes	Bacteroidia	Bacteroidales	Rikenellaceae	Alistipes	109	0.626087	392.895791
Alistipes sp. dk3624	Bacteroidetes	Bacteroidia	Bacteroidales	Rikenellaceae	Alistipes	97	0.591781	248.852392
Alistipes finegoldii	Bacteroidetes	Bacteroidia	Bacteroidales	Rikenellaceae	Alistipes	103	0.615385	305.309087
Alistipes onderdonkii	Bacteroidetes	Bacteroidia	Bacteroidales	Rikenellaceae	Alistipes	96	0.595041	409.373913
Alistipes dispar	Bacteroidetes	Bacteroidia	Bacteroidales	Rikenellaceae	Alistipes	108	0.624277	409.200802
Muribaculum gordoncarteri	Bacteroidetes	Bacteroidia	Bacteroidales	Muribaculaceae	Muribaculum	98	0.579088	126.760647
Muribaculum intestinale	Bacteroidetes	Bacteroidia	Bacteroidales	Muribaculaceae	Muribaculum	102	0.583784	177.168437
Duncaniella dubosii	Bacteroidetes	Bacteroidia	Bacteroidales	Muribaculaceae	Duncaniella	99	0.580645	188.677957
Petrimonas mucosa	Bacteroidetes	Bacteroidia	Bacteroidales	Dysgonomonadaceae	Petrimonas	105	0.590164	192.064529
Proteiniphilum saccharofermentans	Bacteroidetes	Bacteroidia	Bacteroidales	Dysgonomonadaceae	Proteiniphilum	103	0.585366	184.226062
Barnesiella viscericola	Bacteroidetes	Bacteroidia	Bacteroidales	Barnesiellaceae	Barnesiella	99	0.585366	185.629732
Porphyromonas gingivalis	Bacteroidetes	Bacteroidia	Bacteroidales	Porphyromonadaceae	Porphyromonas	106	0.593407	230.187398
Porphyromonas asaccharolytica	Bacteroidetes	Bacteroidia	Bacteroidales	Porphyromonadaceae	Porphyromonas	84	0.566929	101.006924
Pseudomonas versuta	Proteobacteria	Gammaproteobacteria	Pseudomonadales	Pseudomonadaceae	Pseudomonas	23	0.45	417.239871
Pseudomonas taetrolens	Proteobacteria	Gammaproteobacteria	Pseudomonadales	Pseudomonadaceae	Pseudomonas	21	0.451883	386.030012

Species	phylum	class	order	family	genus	Node degree	closnesscentrality	betweenesscentrality
Pseudomonas stutzeri	Proteobacteria	Gammaproteobacteria	Pseudomonadales	Pseudomonadaceae	Pseudomonas	25	0.436364	1019.198857
Aeromonas veronii	Proteobacteria	Gammaproteobacteria	Aeromonadales	Aeromonadaceae	Aeromonas	4	0.330275	0
Escherichia coli	Proteobacteria	Gammaproteobacteria	Enterobacterales	Enterobacteriaceae	Escherichia	22	0.433735	35.077373
Escherichia albertii	Proteobacteria	Gammaproteobacteria	Enterobacterales	Enterobacteriaceae	Escherichia	25	0.416185	23.031872
Escherichia fergusonii	Proteobacteria	Gammaproteobacteria	Enterobacterales	Enterobacteriaceae	Escherichia	22	0.398524	10.772875
Escherichia marmotae	Proteobacteria	Gammaproteobacteria	Enterobacterales	Enterobacteriaceae	Escherichia	32	0.492027	141.02151
Citrobacter tructae	Proteobacteria	Gammaproteobacteria	Enterobacterales	Enterobacteriaceae	Citrobacter	32	0.446281	56.338338
Enterobacter cloacae	Pseudomonadota	Gammaproteobacteria	Enterobacterales	Enterobacteriaceae	Enterobacter	24	0.428571	22.818161
Enterobacter kobei	Proteobacteria	Gammaproteobacteria	Enterobacterales	Enterobacteriaceae	Enterobacter	22	0.425197	16.789614
Enterobacter roggenkampii	Proteobacteria	Gammaproteobacteria	Enterobacterales	Enterobacteriaceae	Enterobacter	18	0.383659	7.449011
Enterobacter bugandensis	Proteobacteria	Gammaproteobacteria	Enterobacterales	Enterobacteriaceae	Enterobacter	17	0.36	0
Klebsiella pneumoniae	Proteobacteria	Gammaproteobacteria	Enterobacterales	Enterobacteriaceae	Klebsiella	28	0.470588	58.896336
Klebsiella oxytoca	Proteobacteria	Gammaproteobacteria	Enterobacterales	Enterobacteriaceae	Klebsiella	41	0.508235	195.237815
Klebsiella quasipneumoniae	Proteobacteria	Gammaproteobacteria	Enterobacterales	Enterobacteriaceae	Klebsiella	29	0.427723	28.608793
Klebsiella aerogenes	Proteobacteria	Gammaproteobacteria	Enterobacterales	Enterobacteriaceae	Klebsiella	33	0.492027	115.86498
Klebsiella sp. FDAARGOS_511	Proteobacteria	Gammaproteobacteria	Enterobacterales	Enterobacteriaceae	Klebsiella	28	0.463519	40.662037
Klebsiella variicola	Proteobacteria	Gammaproteobacteria	Enterobacterales	Enterobacteriaceae	Klebsiella	17	0.36	0
Raoultella ornithinolytica	Proteobacteria	Gammaproteobacteria	Enterobacterales	Enterobacteriaceae	Raoultella	35	0.466523	93.048436
Raoultella planticola	Proteobacteria	Gammaproteobacteria	Enterobacterales	Enterobacteriaceae	Raoultella	38	0.458599	155.711165
Lelliottia sp. WB101	Proteobacteria	Gammaproteobacteria	Enterobacterales	Enterobacteriaceae	Lelliottia	22	0.385714	56.959457
Yersinia enterocolitica	Proteobacteria	Gammaproteobacteria	Enterobacterales	Yersiniaceae	Yersinia	33	0.509434	338.049188
Acinetobacter johnsonii	Proteobacteria	Gammaproteobacteria	Moraxellales	Moraxellaceae	Acinetobacter	6	0.36	117.092291
Psychrobacter sp. P11G5	Proteobacteria	Gammaproteobacteria	Moraxellales	Moraxellaceae	Psychrobacter	4	0.280519	5.120074
Psychrobacter sp. P11F6	Proteobacteria	Gammaproteobacteria	Moraxellales	Moraxellaceae	Psychrobacter	8	0.357616	173.595392
Haemophilus parainfluenzae	Proteobacteria	Gammaproteobacteria	Pasteurellales	Pasteurellaceae	Haemophilus	5	0.357616	3.663725
Alcaligenes faecalis	Proteobacteria	Betaproteobacteria	Burkholderiales	Alcaligenaceae	Alcaligenes	8	0.334365	368.510575
Alcaligenes aquatilis	Proteobacteria	Betaproteobacteria	Burkholderiales	Alcaligenaceae	Alcaligenes	1	0.250871	0
Comamonas kerstersii	Proteobacteria	Betaproteobacteria	Burkholderiales	Comamonadaceae	Comamonas	2	0.305949	0
Oxalobacter formigenes	Proteobacteria	Betaproteobacteria	Burkholderiales	Oxalobacteraceae	Oxalobacter	21	0.419417	1.801938
Sutterella megalosphaeroides	Proteobacteria	Betaproteobacteria	Burkholderiales	Sutterellaceae	Sutterella	50	0.48	5.001787
Sutterella faecalis	Proteobacteria	Betaproteobacteria	Burkholderiales	Sutterellaceae	Sutterella	55	0.516746	23.266357
Brevundimonas diminuta	Proteobacteria	Alphaproteobacteria	Caulobacterales	Caulobacteraceae	Brevundimonas	5	0.255018	5.691129
Brevundimonas naejangsanensis	Proteobacteria	Alphaproteobacteria	Caulobacterales	Caulobacteraceae	Brevundimonas	5	0.3099	181.497181
Desulfovibrio piger	Proteobacteria	Deltaproteobacteria	Desulfovibrionales	Desulfovibrionaceae	Desulfovibrio	50	0.513064	22.982991
Campylobacter jejuni	Proteobacteria	Epsilonproteobacteria	Campylobacterales	Campylobacteraceae	Campylobacter	30	0.439024	0.417816
Lachnospiraceae bacterium Choco86	Firmicutes	Clostridia	Eubacteriales	Lachnospiraceae		52	0.524272	129.295981
Lachnospiraceae bacterium	Bacillota	Clostridia	Eubacteriales	Lachnospiraceae		65	0.524272	87.27843
Lachnospiraceae bacterium KM106-2	Firmicutes	Clostridia	Eubacteriales	Lachnospiraceae		46	0.492027	74.466599
Lachnospiraceae bacterium KGMB03038	Firmicutes	Clostridia	Eubacteriales	Lachnospiraceae		69	0.548223	164.661544
[Ruminococcus] gnavus	Bacillota	Clostridia	Eubacteriales	Lachnospiraceae	Mediterraneibacter	71	0.544081	258.624612
Blautia sp. SC05B48	Firmicutes	Clostridia	Eubacteriales	Lachnospiraceae	Blautia	57	0.520482	161.08196
Blautia argi	Firmicutes	Clostridia	Eubacteriales	Lachnospiraceae	Blautia	77	0.549618	169.283106
Blautia producta	Firmicutes	Clostridia	Eubacteriales	Lachnospiraceae	Blautia	71	0.538653	120.590138
Anaerostipes hadrus	Firmicutes	Clostridia	Eubacteriales	Lachnospiraceae	Anaerostipes	46	0.483221	10.982687

Species	phylum	class	order	family	genus	Node degree	closnesscentrality	betweenesscentrality
Anaerostipes rhamnosivorans	Firmicutes	Clostridia	Eubacteriales	Lachnospiraceae	Anaerostipes	58	0.510638	48.049798
Anaerobutyricum hallii	Firmicutes	Clostridia	Eubacteriales	Lachnospiraceae	Anaerobutyricum	54	0.495413	202.817902
[Clostridium] hylemonae	Bacillota	Clostridia	Eubacteriales	Lachnospiraceae	Lachnoclostridium	66	0.544081	143.71577
[Clostridium] scindens	Bacillota	Clostridia	Eubacteriales	Lachnospiraceae	Lachnoclostridium	66	0.546835	205.495092
Lachnoclostridium sp. YL32	Firmicutes	Clostridia	Eubacteriales	Lachnospiraceae	Lachnoclostridium	56	0.511848	46.654474
Lachnoclostridium phocaeense	Firmicutes	Clostridia	Eubacteriales	Lachnospiraceae	Lachnoclostridium	66	0.545455	142.238703
Lachnoclostridium phytofermentans	Firmicutes	Clostridia	Eubacteriales	Lachnospiraceae	Lachnoclostridium	66	0.549618	214.194948
Enterocloster bolteae	Firmicutes	Clostridia	Eubacteriales	Lachnospiraceae	Enterocloster	39	0.473684	24.502482
Enterocloster clostridioformis	Firmicutes	Clostridia	Eubacteriales	Lachnospiraceae	Enterocloster	50	0.490909	22.394754
Lachnospira eligens	Firmicutes	Clostridia	Eubacteriales	Lachnospiraceae	Lachnospira	16	0.442623	2.875208
Lacrimispora sphenoides	Firmicutes	Clostridia	Eubacteriales	Lachnospiraceae	Lacrimispora	57	0.526829	143.353859
Lacrimispora saccharolytica	Firmicutes	Clostridia	Eubacteriales	Lachnospiraceae	Lacrimispora	48	0.486486	30.035589
Anaerocolumna sedimenticola	Firmicutes	Clostridia	Eubacteriales	Lachnospiraceae	Anaerocolumna	62	0.541353	131.133814
Lachnoanaerobaculum umeaense	Firmicutes	Clostridia	Eubacteriales	Lachnospiraceae	Lachnoanaerobaculum	59	0.533333	152.110748
Anaerotignum propionicum	Firmicutes	Clostridia	Eubacteriales	Lachnospiraceae	Anaerotignum	46	0.50116	89.109984
Faecalibacterium prausnitzii	Firmicutes	Clostridia	Eubacteriales	Oscillospiraceae	Faecalibacterium	41	0.520482	112.723049
Ruminococcus sp. JE7A12	Firmicutes	Clostridia	Eubacteriales	Oscillospiraceae	Ruminococcus	14	0.40678	0.436185
Ruminococcus bicirculans	Firmicutes	Clostridia	Eubacteriales	Oscillospiraceae	Ruminococcus	28	0.476821	18.624995
Ruminococcus albus	Firmicutes	Clostridia	Eubacteriales	Oscillospiraceae	Ruminococcus	70	0.55243	264.473215
Ruminococcus champanellensis	Firmicutes	Clostridia	Eubacteriales	Oscillospiraceae	Ruminococcus	78	0.572944	459.555245
Dysosmobacter welbionis	Firmicutes	Clostridia	Eubacteriales	Oscillospiraceae	Dysosmobacter	70	0.55243	255.305469
Oscillibacter sp. PEA192	Firmicutes	Clostridia	Eubacteriales	Oscillospiraceae	Oscillibacter	67	0.549618	195.337419
Oscillibacter valericigenes	Firmicutes	Clostridia	Eubacteriales	Oscillospiraceae	Oscillibacter	76	0.568421	419.782809
Caproiciproducens sp. NJN-50	Firmicutes	Clostridia	Eubacteriales	Oscillospiraceae	Caproiciproducens	67	0.549618	357.497833
Ethanoligenens harbinense	Firmicutes	Clostridia	Eubacteriales	Oscillospiraceae	Ethanoligenens	73	0.568421	853.904895
Clostridioides difficile	Firmicutes	Clostridia	Eubacteriales	Peptostreptococcaceae	Clostridioides	90	0.572944	386.405026
Peptacetobacter hiranonis	Firmicutes	Clostridia	Eubacteriales	Peptostreptococcaceae	Peptacetobacter	61	0.493151	17.789707
Massilistercora timonensis	Firmicutes	Clostridia	Eubacteriales		Massilistercora	37	0.465517	6.204223
Flintibacter sp. KGMB00164	Firmicutes	Clostridia	Eubacteriales		Flintibacter	74	0.561039	326.686905
Mogibacterium diversum	Firmicutes	Clostridia	Eubacteriales	Eubacteriales_Family_XIII._Incertae_Sedis	Mogibacterium	76	0.537313	46.855938
[Eubacterium] sulci	Bacillota	Clostridia	Eubacteriales	Eubacteriales_Family_XIII._Incertae_Sedis		64	0.507042	16.780278
Intestinimonas butyriciproducens	Firmicutes	Clostridia	Eubacteriales		Intestinimonas	64	0.546835	196.042023
Clostridium sp. SY8519	Firmicutes	Clostridia	Eubacteriales	Clostridiaceae	Clostridium	49	0.508235	162.715343
Clostridium sp. DL-VIII	Firmicutes	Clostridia	Eubacteriales	Clostridiaceae	Clostridium	60	0.511848	56.84009
Clostridium perfringens	Firmicutes	Clostridia	Eubacteriales	Clostridiaceae	Clostridium	45	0.475771	6.644287
Clostridium botulinum	Firmicutes	Clostridia	Eubacteriales	Clostridiaceae	Clostridium	46	0.490909	11.101235
Clostridium butyricum	Firmicutes	Clostridia	Eubacteriales	Clostridiaceae	Clostridium	47	0.477876	7.8731
Clostridium pasteurianum	Firmicutes	Clostridia	Eubacteriales	Clostridiaceae	Clostridium	43	0.493151	64.222405
Clostridium saccharoperbutylacetonicum	Firmicutes	Clostridia	Eubacteriales	Clostridiaceae	Clostridium	47	0.495413	35.480882
Clostridium beijerinckii	Firmicutes	Clostridia	Eubacteriales	Clostridiaceae	Clostridium	58	0.509434	24.153699
Clostridium bornimense	Firmicutes	Clostridia	Eubacteriales	Clostridiaceae	Clostridium	37	0.467532	3.855594
Clostridium baratii	Firmicutes	Clostridia	Eubacteriales	Clostridiaceae	Clostridium	38	0.45283	2.810997

Species	phylum	class	order	family	genus	Node degree	closnesscentrality	betweenesscentrality
Clostridium isatidis	Firmicutes	Clostridia	Eubacteriales	Clostridiaceae	Clostridium	28	0.431138	1.132017
Clostridium saccharobutylicum	Firmicutes	Clostridia	Eubacteriales	Clostridiaceae	Clostridium	52	0.482143	11.279583
Clostridium chauvoei	Firmicutes	Clostridia	Eubacteriales	Clostridiaceae	Clostridium	33	0.439024	1.974916
Clostridium septicum	Firmicutes	Clostridia	Eubacteriales	Clostridiaceae	Clostridium	38	0.451883	2.941507
Clostridium novyi	Firmicutes	Clostridia	Eubacteriales	Clostridiaceae	Clostridium	57	0.505855	48.424704
Clostridium taeniosporum	Firmicutes	Clostridia	Eubacteriales	Clostridiaceae	Clostridium	42	0.471616	4.931976
Hungatella hathewayi	Firmicutes	Clostridia	Eubacteriales	Clostridiaceae	Hungatella	43	0.492027	45.650066
Clostridiales bacterium CCNA10	Firmicutes	Clostridia	Eubacteriales			65	0.541353	351.033939
Christensenella minuta	Firmicutes	Clostridia	Eubacteriales	Christensenellaceae	Christensenella	22	0.445361	8.20106
Christensenella sp. Marseille-P3954	Firmicutes	Clostridia	Eubacteriales	Christensenellaceae	Christensenella	55	0.515513	349.737066
Eubacterium limosum	Firmicutes	Clostridia	Eubacteriales	Eubacteriaceae	Eubacterium	57	0.513064	33.57024
Eubacterium callanderi	Firmicutes	Clostridia	Eubacteriales	Eubacteriaceae	Eubacterium	49	0.487585	18.662703
Enterococcus avium	Firmicutes	Bacilli	Lactobacillales	Enterococcaceae	Enterococcus	29	0.505855	126.580623
Enterococcus casseliflavus	Firmicutes	Bacilli	Lactobacillales	Enterococcaceae	Enterococcus	39	0.519231	482.290595
Enterococcus faecium	Firmicutes	Bacilli	Lactobacillales	Enterococcaceae	Enterococcus	55	0.530713	271.600472
Streptococcus salivarius	Firmicutes	Bacilli	Lactobacillales	Streptococcaceae	Streptococcus	62	0.519231	36.139517
Streptococcus vestibularis	Firmicutes	Bacilli	Lactobacillales	Streptococcaceae	Streptococcus	59	0.513064	15.603863
Streptococcus sp. LPB0220	Firmicutes	Bacilli	Lactobacillales	Streptococcaceae	Streptococcus	54	0.503497	57.576605
Streptococcus sp. HSISM1	Firmicutes	Bacilli	Lactobacillales	Streptococcaceae	Streptococcus	56	0.505855	61.936253
Streptococcus sp. A12	Firmicutes	Bacilli	Lactobacillales	Streptococcaceae	Streptococcus	72	0.529412	31.96438
Streptococcus sp. oral taxon 431	Firmicutes	Bacilli	Lactobacillales	Streptococcaceae	Streptococcus	75	0.528117	69.14855
Streptococcus sp. FDAARGOS_192	Firmicutes	Bacilli	Lactobacillales	Streptococcaceae	Streptococcus	62	0.519231	36.139517
Streptococcus pneumoniae	Firmicutes	Bacilli	Lactobacillales	Streptococcaceae	Streptococcus	88	0.583784	166.352506
Streptococcus dysgalactiae	Firmicutes	Bacilli	Lactobacillales	Streptococcaceae	Streptococcus	23	0.416185	0.348408
Streptococcus pasteurianus	Firmicutes	Bacilli	Lactobacillales	Streptococcaceae	Streptococcus	47	0.488688	65.383209
Streptococcus thermophilus	Firmicutes	Bacilli	Lactobacillales	Streptococcaceae	Streptococcus	54	0.490909	4.451198
Streptococcus mitis	Firmicutes	Bacilli	Lactobacillales	Streptococcaceae	Streptococcus	72	0.524272	52.344168
Streptococcus sanguinis	Firmicutes	Bacilli	Lactobacillales	Streptococcaceae	Streptococcus	82	0.55527	69.842176
Streptococcus ilei	Firmicutes	Bacilli	Lactobacillales	Streptococcaceae	Streptococcus	73	0.530713	32.607135
Streptococcus parasanguinis	Firmicutes	Bacilli	Lactobacillales	Streptococcaceae	Streptococcus	58	0.507042	28.728717
Streptococcus oralis	Firmicutes	Bacilli	Lactobacillales	Streptococcaceae	Streptococcus	70	0.520482	41.792058
Streptococcus cristatus	Firmicutes	Bacilli	Lactobacillales	Streptococcaceae	Streptococcus	65	0.520482	32.627762
Streptococcus equinus	Firmicutes	Bacilli	Lactobacillales	Streptococcaceae	Streptococcus	71	0.54	134.924602
Streptococcus australis	Firmicutes	Bacilli	Lactobacillales	Streptococcaceae	Streptococcus	79	0.55102	56.02615
Streptococcus koreensis	Firmicutes	Bacilli	Lactobacillales	Streptococcaceae	Streptococcus	73	0.530713	32.607135
Streptococcus gordonii	Firmicutes	Bacilli	Lactobacillales	Streptococcaceae	Streptococcus	68	0.53202	99.260173
Streptococcus viridans	Firmicutes	Bacilli	Lactobacillales	Streptococcaceae	Streptococcus	76	0.534653	40.352325
Streptococcus infantarius	Firmicutes	Bacilli	Lactobacillales	Streptococcaceae	Streptococcus	42	0.469565	4.400193
Lactococcus lactis	Firmicutes	Bacilli	Lactobacillales	Streptococcaceae	Lactococcus	11	0.430279	3.198082
Lactococcus cremoris	Firmicutes	Bacilli	Lactobacillales	Streptococcaceae	Lactococcus	15	0.421875	1.0557
Lactobacillus johnsonii	Firmicutes	Bacilli	Lactobacillales	Lactobacillaceae	Lactobacillus	11	0.424361	373.94955
Lactobacillus delbrueckii	Firmicutes	Bacilli	Lactobacillales	Lactobacillaceae	Lactobacillus	2	0.299169	0
Ligilactobacillus ruminis	Firmicutes	Bacilli	Lactobacillales	Lactobacillaceae	Ligilactobacillus	9	0.417795	134.625479
Leuconostoc mesenteroides	Firmicutes	Bacilli	Lactobacillales	Lactobacillaceae	Leuconostoc	18	0.422701	4.965996

Species	phylum	class	order	family	genus	Node degree	closnesscentrality	betweenesscentrality
Limosilactobacillus mucosae	Firmicutes	Bacilli	Lactobacillales	Lactobacillaceae	Limosilactobacillus	3	0.305949	5.424847
Fructilactobacillus sanfranciscensis	Firmicutes	Bacilli	Lactobacillales	Lactobacillaceae	Fructilactobacillus	8	0.388489	0.72981
Carnobacterium maltaromaticum	Firmicutes	Bacilli	Lactobacillales	Carnobacteriaceae	Carnobacterium	69	0.546835	1147.804124
Bacillus sp. N3536	Firmicutes	Bacilli	Bacillales	Bacillaceae	Bacillus	8	0.393443	137.876555
Psychrobacillus glaciei	Firmicutes	Bacilli	Bacillales	Bacillaceae	Psychrobacillus	12	0.413793	66.474459
Psychrobacillus sp. AK 1817	Firmicutes	Bacilli	Bacillales	Bacillaceae	Psychrobacillus	9	0.401487	152.923321
Sporosarcina psychrophila	Firmicutes	Bacilli	Bacillales	Planococcaceae	Sporosarcina	11	0.402985	340.077585
Gemella sanguinis	Firmicutes	Bacilli	Bacillales		Gemella	80	0.549618	51.689632
[Clostridium] innocuum	Bacillota	Erysipelotrichia	Erysipelotrichales	Coprobacillaceae	Thomasclavelia	51	0.497696	47.316188
Erysipelotrichaceae bacterium GAM147	Firmicutes	Erysipelotrichia	Erysipelotrichales	Erysipelotrichaceae		30	0.455696	3.854506
Absiella argi	Firmicutes	Erysipelotrichia	Erysipelotrichales	Erysipelotrichaceae	Absiella	69	0.525547	88.577669
Faecalitalea cylindroides	Firmicutes	Erysipelotrichia	Erysipelotrichales	Erysipelotrichaceae	Faecalitalea	42	0.486486	374.18767
Amedibacterium intestinale	Firmicutes	Erysipelotrichia	Erysipelotrichales	Erysipelotrichaceae	Amedibacterium	41	0.473684	12.909413
Intestinibaculum porci	Firmicutes	Erysipelotrichia	Erysipelotrichales	Erysipelotrichaceae	Intestinibaculum	22	0.451883	11.577441
Megamonas funiformis	Firmicutes	Negativicutes	Selenomonadales	Selenomonadaceae	Megamonas	1	1	0
Megamonas hypermegale	Firmicutes	Negativicutes	Selenomonadales	Selenomonadaceae	Megamonas	1	1	0
Veillonella atypica	Firmicutes	Negativicutes	Veillonellales	Veillonellaceae	Veillonella	14	0.400742	48.401408
Dialister massiliensis	Firmicutes	Negativicutes	Veillonellales	Veillonellaceae	Dialister	2	0.36425	215
Dialister hominis	Firmicutes	Negativicutes	Veillonellales	Veillonellaceae	Dialister	1	0.267327	0
Finegoldia magna	Firmicutes	Tissierellia	Tissierellales	Peptoniphilaceae	Finegoldia	60	0.50116	25.875947
Bifidobacterium longum	Actinobacteria	Actinomycetia	Bifidobacteriales	Bifidobacteriaceae	Bifidobacterium	9	0.393443	23.949785
Bifidobacterium adolescentis	Actinobacteria	Actinomycetia	Bifidobacteriales	Bifidobacteriaceae	Bifidobacterium	9	0.361204	16.981639
Bifidobacterium breve	Actinobacteria	Actinomycetia	Bifidobacteriales	Bifidobacteriaceae	Bifidobacterium	46	0.497696	825.841721
Bifidobacterium catenulatum	Actinobacteria	Actinomycetia	Bifidobacteriales	Bifidobacteriaceae	Bifidobacterium	8	0.352941	3.111376
Bifidobacterium bifidum	Actinobacteria	Actinomycetia	Bifidobacteriales	Bifidobacteriaceae	Bifidobacterium	8	0.352941	3.111376
Bifidobacterium angulatum	Actinobacteria	Actinomycetia	Bifidobacteriales	Bifidobacteriaceae	Bifidobacterium	10	0.361809	31.023177
Bifidobacterium pseudocatenulatum	Actinobacteria	Actinomycetia	Bifidobacteriales	Bifidobacteriaceae	Bifidobacterium	7	0.342314	0
Bifidobacterium dentium	Actinobacteria	Actinomycetia	Bifidobacteriales	Bifidobacteriaceae	Bifidobacterium	12	0.380952	108.65881
Actinomyces sp. oral taxon 414	Actinobacteria	Actinomycetia	Actinomycetales	Actinomycetaceae	Actinomyces	84	0.563969	136.441758
Schaalia odontolytica	Actinobacteria	Actinomycetia	Actinomycetales	Actinomycetaceae	Schaalia	87	0.571429	101.063473
Rhodococcus erythropolis	Actinobacteria	Actinomycetia	Corynebacteriales	Nocardiaceae	Rhodococcus	2	0.228814	0
Microbacterium oxydans	Actinobacteria	Actinomycetia	Micrococcales	Microbacteriaceae	Microbacterium	10	0.29589	319.401382
Arthrobacter sp. YC-RL1	Actinobacteria	Actinomycetia	Micrococcales	Micrococcaceae	Arthrobacter	8	0.296296	52.093759
Rothia mucilaginosa	Actinobacteria	Actinomycetia	Micrococcales	Micrococcaceae	Rothia	59	0.515513	168.35646
Oerskovia sp. KBS0722	Actinobacteria	Actinomycetia	Micrococcales	Cellulomonadaceae	Oerskovia	5	0.24714	8.936718
Gordonibacter urolithinfaciens	Actinobacteria	Coriobacteriia	Eggerthellales	Eggerthellaceae	Gordonibacter	11	0.412214	50.767698
Gordonibacter pamelaeae	Actinobacteria	Coriobacteriia	Eggerthellales	Eggerthellaceae	Gordonibacter	10	0.4	25.809643
Arabia massiliensis	Actinobacteria	Coriobacteriia	Eggerthellales	Eggerthellaceae	Arabia	10	0.407547	17.602499
Adlercreutzia sp. 8CFCBH1	Actinobacteria	Coriobacteriia	Eggerthellales	Eggerthellaceae	Adlercreutzia	25	0.473684	175.01871
Adlercreutzia equolifaciens	Actinobacteria	Coriobacteriia	Eggerthellales	Eggerthellaceae	Adlercreutzia	28	0.477876	225.194334
Collinsella aerofaciens	Actinobacteria	Coriobacteriia	Coriobacteriales	Coriobacteriaceae	Collinsella	5	0.336973	6.399431
Olsenella sp. GAM18	Actinobacteria	Coriobacteriia	Coriobacteriales	Atopobiaceae	Olsenella	15	0.409867	133.846675
Akkermansia muciniphila	Verrucomicrobia	Verrucomicrobiae	Verrucomicrobiales	Akkermansiaceae	Akkermansia	15	0.445361	0.801275
Fusobacterium varium	Fusobacteria	Fusobacteriia	Fusobacteriales	Fusobacteriaceae	Fusobacterium	17	0.451883	79.939483

Species	phylum	class	order	family	genus	Node degree	closnesscentrality	betweenesscentrality
Fusobacterium ulcerans	Fusobacteria	Fusobacteriia	Fusobacteriales	Fusobacteriaceae	Fusobacterium	17	0.415385	58.89734
Fusobacterium nucleatum	Fusobacteria	Fusobacteriia	Fusobacteriales	Fusobacteriaceae	Fusobacterium	28	0.434608	163.135496
Fusobacterium mortiferum	Fusobacteria	Fusobacteriia	Fusobacteriales	Fusobacteriaceae	Fusobacterium	3	0.33282	0
Cloacibacillus porcorum	Synergistetes	Synergistia	Synergistales	Synergistaceae	Cloacibacillus	21	0.463519	13.87108

Supplementary Table 7 Microbial functions analysis

Pathway	coef	stderr	p value	q value
PWY-6435: 4-hydroxybenzoate biosynthesis III (plants)	0.4407873	0.1240134	0.0003972	0.080238
PWY0-461: L-lysine degradation I	0.394909	0.1193664	0.0009725	0.1204512
PWY-5692: allantoin degradation to glyoxylate II	0.3333557	0.1159075	0.004114	0.1692441
URDEGR-PWY: superpathway of allantoin degradation in plants	0.3333557	0.1159075	0.004114	0.1692441
PWY-5367: petroselinate biosynthesis	0.2282213	0.0716112	0.0014907	0.1204512
SALVADEHYPOX-PWY: adenosine nucleotides degradation II	0.1349423	0.0360585	0.0001929	0.0779381
PWY-6612: superpathway of tetrahydrofolate biosynthesis	0.1241025	0.0424922	0.003574	0.1692441
FOLSYN-PWY: superpathway of tetrahydrofolate biosynthesis and salvage	0.1193722	0.0415886	0.0041892	0.1692441
PWY-6606: guanosine nucleotides degradation II	0.0974208	0.0305369	0.0014665	0.1204512
PWY-6353: purine nucleotides degradation II (aerobic)	0.0896611	0.0310736	0.0039942	0.1692441

Supplementary Table 8 Mediating effect of gut microbiota between HLBS and dyslipidemia

Species	ACME	ADE	Proportion	P value of proportion
Bacteroides intestinalis	-0.001 (-0.004 to 0)	-0.085 (-0.099 to -0.066)	0.014 (-0.005 to 0.049)	0.140
Aeromonas veronii	-0.004 (-0.009 to -0.001)	-0.082 (-0.097 to -0.063)	0.048 (0.013 to 0.111)	0.004
Escherichia coli	-0.002 (-0.005 to 0)	-0.085 (-0.099 to -0.066)	0.017 (-0.003 to 0.055)	0.080
Escherichia marmotae	-0.002 (-0.005 to 0)	-0.084 (-0.099 to -0.065)	0.024 (0.001 to 0.064)	0.034
Psychrobacter sp. P11G5	0 (-0.003 to 0.002)	-0.086 (-0.1 to -0.069)	0.004 (-0.019 to 0.034)	0.636
Haemophilus parainfluenzae	-0.002 (-0.005 to 0)	-0.085 (-0.099 to -0.067)	0.021 (-0.006 to 0.06)	0.110
Brevundimonas naejangsanensis	-0.002 (-0.004 to 0)	-0.085 (-0.099 to -0.065)	0.017 (-0.005 to 0.052)	0.114
Lachnospiraceae bacterium Choco86	0 (-0.003 to 0.002)	-0.086 (-0.099 to -0.066)	0.004 (-0.02 to 0.032)	0.732
Lachnospiraceae bacterium	-0.003 (-0.007 to -0.001)	-0.083 (-0.098 to -0.065)	0.038 (0.008 to 0.089)	0.008
Lachnospiraceae bacterium KGMB03038	0 (-0.002 to 0.002)	-0.086 (-0.1 to -0.068)	0.001 (-0.026 to 0.028)	0.914
[Ruminococcus] gnavus	-0.007 (-0.012 to -0.002)	-0.079 (-0.094 to -0.059)	0.075 (0.029 to 0.146)	0.000
Blautia argi	-0.002 (-0.005 to 0)	-0.084 (-0.098 to -0.066)	0.025 (0 to 0.065)	0.046
[Clostridium] hylemonae	-0.001 (-0.003 to 0.001)	-0.085 (-0.098 to -0.067)	0.006 (-0.016 to 0.037)	0.508
[Clostridium] scindens	-0.001 (-0.003 to 0.001)	-0.086 (-0.099 to -0.067)	0.008 (-0.015 to 0.039)	0.472
Lachnoclostridium phocaeense	0 (-0.002 to 0.002)	-0.086 (-0.101 to -0.065)	-0.001 (-0.025 to 0.025)	0.946
Lachnospira eligens	-0.002 (-0.005 to 0)	-0.084 (-0.098 to -0.066)	0.021 (-0.002 to 0.058)	0.066
Anaerotignum propionicum	0 (-0.002 to 0.003)	-0.087 (-0.1 to -0.07)	-0.001 (-0.033 to 0.02)	0.846
Ruminococcus bicirculans	-0.003 (-0.006 to 0)	-0.084 (-0.098 to -0.065)	0.027 (0.003 to 0.068)	0.016
Ruminococcus albus	-0.003 (-0.007 to 0)	-0.083 (-0.098 to -0.063)	0.035 (0.005 to 0.087)	0.024
Eubacterium limosum	0 (-0.003 to 0.002)	-0.086 (-0.1 to -0.067)	0.002 (-0.025 to 0.033)	0.868
Streptococcus vestibularis	-0.002 (-0.005 to 0)	-0.085 (-0.098 to -0.067)	0.017 (-0.006 to 0.053)	0.118
Streptococcus sp. LPB0220	-0.002 (-0.006 to 0)	-0.084 (-0.098 to -0.067)	0.024 (0.002 to 0.067)	0.028
Streptococcus sp. HSISM1	-0.002 (-0.005 to 0)	-0.085 (-0.099 to -0.066)	0.018 (-0.002 to 0.055)	0.082
Streptococcus pneumoniae	-0.002 (-0.005 to 0)	-0.084 (-0.098 to -0.064)	0.023 (0.002 to 0.061)	0.020
Streptococcus mitis	-0.003 (-0.007 to -0.001)	-0.084 (-0.098 to -0.066)	0.032 (0.006 to 0.08)	0.014
Streptococcus parasanguinis	-0.002 (-0.006 to 0)	-0.084 (-0.098 to -0.063)	0.026 (0.002 to 0.068)	0.024
Lactococcus cremoris	0 (-0.002 to 0.001)	-0.086 (-0.1 to -0.068)	0.002 (-0.011 to 0.023)	0.650
Ligilactobacillus ruminis	0 (-0.001 to 0.001)	-0.086 (-0.1 to -0.068)	0 (-0.011 to 0.009)	0.972
Sporosarcina psychrophila	0 (-0.003 to 0.002)	-0.085 (-0.1 to -0.065)	0.004 (-0.022 to 0.03)	0.692
Faecalitalea cylindroides	0 (-0.001 to 0.002)	-0.087 (-0.101 to -0.068)	-0.002 (-0.022 to 0.011)	0.664
Amedibacterium intestinale	-0.003 (-0.007 to -0.001)	-0.083 (-0.097 to -0.066)	0.035 (0.007 to 0.081)	0.004
Megamonas funiformis	-0.003 (-0.006 to 0)	-0.084 (-0.098 to -0.065)	0.028 (0.002 to 0.075)	0.036
Megamonas hypermegale	-0.003 (-0.006 to 0)	-0.084 (-0.099 to -0.064)	0.028 (0.003 to 0.07)	0.034
Finegoldia magna	-0.001 (-0.004 to 0)	-0.085 (-0.099 to -0.066)	0.014 (-0.006 to 0.05)	0.150
Bifidobacterium breve	0 (-0.002 to 0.001)	-0.086 (-0.1 to -0.067)	0.002 (-0.013 to 0.023)	0.716
Bifidobacterium catenulatum	0 (-0.001 to 0.002)	-0.087 (-0.1 to -0.066)	-0.001 (-0.021 to 0.01)	0.732
Bifidobacterium pseudocatenulatum	0.001 (-0.001 to 0.003)	-0.087 (-0.101 to -0.067)	-0.005 (-0.035 to 0.012)	0.458
Fusobacterium varium	-0.003 (-0.007 to -0.001)	-0.083 (-0.098 to -0.061)	0.035 (0.007 to 0.082)	0.014

Supplementary Table 9 Mediating effect of gut microbiota between HLBS and decreased HDL

Species	ACME	ADE	Proportion	P value of proportion
Bacteroides uniformis	-0.001 (-0.003 to 0.002)	-0.056 (-0.097 to -0.019)	0.008 (-0.041 to 0.073)	0.632
Bacteroides intestinalis	-0.001 (-0.004 to 0.001)	-0.056 (-0.098 to -0.02)	0.008 (-0.026 to 0.077)	0.526
Bacteroides caecimuris	0 (-0.002 to 0.001)	-0.056 (-0.099 to -0.021)	0.002 (-0.026 to 0.04)	0.792
Phocaeicola vulgatus	0 (-0.001 to 0.002)	-0.058 (-0.103 to -0.023)	-0.003 (-0.05 to 0.025)	0.646
Phocaeicola dorei	0 (-0.003 to 0.002)	-0.057 (-0.097 to -0.019)	0.002 (-0.03 to 0.055)	0.782
Prevotella ruminicola	0 (-0.002 to 0.001)	-0.056 (-0.097 to -0.02)	0.001 (-0.028 to 0.035)	0.876
Prevotella dentalis	0 (-0.002 to 0.001)	-0.056 (-0.099 to -0.019)	0 (-0.028 to 0.035)	0.968
Prevotella melaninogenica	0 (-0.002 to 0.001)	-0.058 (-0.099 to -0.021)	0 (-0.025 to 0.027)	0.932
Prevotella fusca	0 (-0.002 to 0.001)	-0.057 (-0.096 to -0.019)	0.001 (-0.031 to 0.042)	0.880
Prevotella scopos	0 (-0.001 to 0.001)	-0.055 (-0.095 to -0.019)	0 (-0.026 to 0.031)	0.890
Enterobacter cloacae	0 (-0.002 to 0.001)	-0.056 (-0.099 to -0.019)	0.003 (-0.022 to 0.052)	0.700
Enterobacter kobei	-0.001 (-0.003 to 0.001)	-0.056 (-0.099 to -0.017)	0.007 (-0.031 to 0.061)	0.598
Enterobacter roggenkampii	-0.002 (-0.005 to 0.001)	-0.054 (-0.097 to -0.017)	0.024 (-0.01 to 0.11)	0.196
Enterobacter bugandensis	0 (-0.002 to 0.001)	-0.058 (-0.099 to -0.02)	0.002 (-0.025 to 0.049)	0.788
Klebsiella pneumoniae	0 (-0.002 to 0.001)	-0.056 (-0.098 to -0.021)	0.002 (-0.03 to 0.046)	0.790
Klebsiella oxytoca	-0.001 (-0.003 to 0.001)	-0.056 (-0.101 to -0.017)	0.01 (-0.019 to 0.068)	0.460
Klebsiella quasipneumoniae	-0.001 (-0.003 to 0.001)	-0.055 (-0.096 to -0.018)	0.009 (-0.022 to 0.071)	0.472
Klebsiella aerogenes	0 (-0.001 to 0.002)	-0.057 (-0.101 to -0.021)	-0.002 (-0.051 to 0.022)	0.724
Klebsiella sp. FDAARGOS_511	-0.001 (-0.005 to 0)	-0.053 (-0.095 to -0.018)	0.023 (-0.01 to 0.115)	0.178
Klebsiella variicola	0 (-0.002 to 0.002)	-0.056 (-0.098 to -0.021)	0.001 (-0.043 to 0.049)	0.858
Raoultella ornithinolytica	-0.001 (-0.005 to 0.001)	-0.055 (-0.095 to -0.019)	0.02 (-0.025 to 0.109)	0.344
Raoultella planticola	-0.003 (-0.008 to 0)	-0.052 (-0.092 to -0.018)	0.053 (-0.005 to 0.183)	0.066
Yersinia enterocolitica	0 (-0.001 to 0.001)	-0.058 (-0.102 to -0.02)	0 (-0.027 to 0.023)	0.986
Acinetobacter johnsonii	0.001 (-0.001 to 0.003)	-0.057 (-0.098 to -0.018)	-0.01 (-0.072 to 0.018)	0.398
Psychrobacter sp. P11F6	0 (-0.004 to 0.003)	-0.058 (-0.1 to -0.022)	0.002 (-0.064 to 0.063)	0.912
Haemophilus parainfluenzae	-0.002 (-0.006 to 0)	-0.054 (-0.095 to -0.018)	0.039 (-0.007 to 0.138)	0.118
Lachnospiraceae bacterium Choco86	0 (-0.003 to 0.002)	-0.055 (-0.097 to -0.018)	0.001 (-0.049 to 0.058)	0.946
Lachnospiraceae bacterium KM106-2	0 (-0.002 to 0.003)	-0.056 (-0.098 to -0.018)	-0.002 (-0.064 to 0.041)	0.838
Lachnospira eligens	-0.002 (-0.005 to 0)	-0.056 (-0.097 to -0.018)	0.026 (-0.007 to 0.106)	0.134
Ruminococcus champanellensis	0.001 (-0.003 to 0.005)	-0.058 (-0.101 to -0.024)	-0.017 (-0.118 to 0.054)	0.568
Dysosmobacter welbionis	0.002 (-0.002 to 0.006)	-0.058 (-0.1 to -0.022)	-0.03 (-0.155 to 0.043)	0.364
Oscillibacter sp. PEA192	0.001 (-0.002 to 0.005)	-0.059 (-0.101 to -0.023)	-0.019 (-0.112 to 0.041)	0.414
Oscillibacter valericigenes	0.001 (-0.003 to 0.005)	-0.057 (-0.1 to -0.02)	-0.011 (-0.104 to 0.06)	0.744
Caproiciproducens sp. NJN-50	0.001 (-0.002 to 0.005)	-0.058 (-0.099 to -0.021)	-0.021 (-0.121 to 0.032)	0.372
Ethanoligenens harbinense	0.002 (-0.002 to 0.006)	-0.058 (-0.097 to -0.021)	-0.023 (-0.136 to 0.031)	0.382
Flintibacter sp. KGMB00164	0.002 (-0.002 to 0.006)	-0.058 (-0.101 to -0.022)	-0.026 (-0.139 to 0.045)	0.370
Intestinimonas butyriciproducens	0 (-0.003 to 0.004)	-0.057 (-0.098 to -0.018)	-0.005 (-0.101 to 0.079)	0.872
Clostridium sp. SY8519	0 (-0.001 to 0.003)	-0.057 (-0.097 to -0.022)	-0.006 (-0.076 to 0.031)	0.620
Clostridiales bacterium CCNA10	0.002 (-0.001 to 0.006)	-0.058 (-0.101 to -0.02)	-0.037 (-0.155 to 0.013)	0.144
Christensenella minuta	0 (-0.002 to 0.001)	-0.055 (-0.097 to -0.019)	0.001 (-0.023 to 0.038)	0.828
Christensenella sp. Marseille-P3954	0.002 (-0.001 to 0.006)	-0.058 (-0.102 to -0.018)	-0.028 (-0.131 to 0.022)	0.240
Enterococcus avium	0 (-0.003 to 0.002)	-0.057 (-0.103 to -0.023)	0.002 (-0.052 to 0.064)	0.852
Enterococcus casseliflavus	0 (-0.003 to 0.002)	-0.055 (-0.097 to -0.017)	0.002 (-0.047 to 0.059)	0.848
Streptococcus ilei	0 (-0.002 to 0.001)	-0.056 (-0.096 to -0.019)	0.001 (-0.026 to 0.042)	0.890
Streptococcus koreensis	0 (-0.002 to 0.001)	-0.057 (-0.099 to -0.021)	0.001 (-0.025 to 0.047)	0.774
Streptococcus viridans	0 (-0.002 to 0.001)	-0.057 (-0.1 to -0.019)	0.002 (-0.029 to 0.051)	0.730
Lactococcus lactis	0 (-0.002 to 0.002)	-0.056 (-0.1 to -0.02)	0 (-0.048 to 0.04)	0.944
Lactococcus cremoris	-0.001 (-0.004 to 0.001)	-0.056 (-0.097 to -0.019)	0.015 (-0.02 to 0.101)	0.344

Species	ACME	ADE	Proportion	P value of proportion
Lactobacillus delbrueckii	0 (-0.001 to 0.001)	-0.057 (-0.101 to -0.019)	0 (-0.029 to 0.022)	0.926
Ligilactobacillus ruminis	0 (-0.002 to 0.001)	-0.056 (-0.1 to -0.021)	0.002 (-0.02 to 0.041)	0.770
Leuconostoc mesenteroides	0 (-0.002 to 0.003)	-0.058 (-0.1 to -0.019)	-0.004 (-0.064 to 0.039)	0.724
Limosilactobacillus mucosae	0 (-0.002 to 0.002)	-0.056 (-0.099 to -0.019)	0 (-0.04 to 0.034)	0.920
Fructilactobacillus sanfranciscensis	0 (-0.001 to 0.001)	-0.057 (-0.099 to -0.019)	-0.001 (-0.034 to 0.021)	0.850
Faecalitalea cylindroides	0 (-0.001 to 0.003)	-0.057 (-0.099 to -0.022)	-0.005 (-0.058 to 0.017)	0.568
Intestinibaculum porci	0 (-0.001 to 0.001)	-0.057 (-0.098 to -0.018)	0 (-0.024 to 0.028)	0.972
Dialister massiliensis	-0.001 (-0.004 to 0.001)	-0.056 (-0.097 to -0.02)	0.012 (-0.024 to 0.08)	0.362
Dialister hominis	-0.001 (-0.005 to 0.001)	-0.054 (-0.096 to -0.017)	0.021 (-0.013 to 0.108)	0.234
Bifidobacterium longum	0.001 (-0.001 to 0.004)	-0.057 (-0.098 to -0.021)	-0.011 (-0.093 to 0.021)	0.438
Bifidobacterium adolescentis	0 (-0.001 to 0.002)	-0.058 (-0.099 to -0.019)	-0.004 (-0.057 to 0.021)	0.630
Bifidobacterium breve	0 (-0.002 to 0.001)	-0.058 (-0.101 to -0.018)	0.001 (-0.032 to 0.035)	0.910
Bifidobacterium catenulatum	0.001 (-0.002 to 0.004)	-0.057 (-0.096 to -0.022)	-0.008 (-0.078 to 0.039)	0.636
Bifidobacterium bifidum	0 (-0.002 to 0.002)	-0.056 (-0.1 to -0.021)	0.003 (-0.029 to 0.051)	0.730
Bifidobacterium angulatum	0 (-0.001 to 0.003)	-0.058 (-0.104 to -0.022)	-0.005 (-0.063 to 0.02)	0.624
Bifidobacterium pseudocatenulatum	0.001 (-0.002 to 0.005)	-0.059 (-0.103 to -0.022)	-0.023 (-0.128 to 0.033)	0.378
Collinsella aerofaciens	0 (-0.001 to 0.001)	-0.057 (-0.099 to -0.021)	0 (-0.033 to 0.023)	0.954
Olsenella sp. GAM18	0 (-0.003 to 0.004)	-0.057 (-0.096 to -0.018)	-0.003 (-0.08 to 0.066)	0.860
Cloacibacillus porcorum	0.003 (-0.001 to 0.008)	-0.059 (-0.101 to -0.022)	-0.049 (-0.183 to 0.021)	0.130

Supplementary Table 10 Mediating effect of gut microbiota between HLBS and elevated LDL

Species	ACME	ADE	Proportion	P value of proportion
[Ruminococcus] gnavus	-0.004 (-0.01 to 0)	-0.072 (-0.12 to -0.025)	0.054 (0.001 to 0.167)	0.048

Supplementary Table 11 Mediating effect of gut microbiota between HLBS and elevated TC

Species	ACME	ADE	Proportion	P value of proportion
Escherichia coli	-0.003 (-0.008 to 0)	-0.111 (-0.159 to -0.06)	0.025 (-0.004 to 0.078)	0.108
Escherichia albertii	-0.003 (-0.007 to 0)	-0.113 (-0.16 to -0.06)	0.02 (-0.002 to 0.065)	0.088
Escherichia fergusonii	-0.002 (-0.007 to 0.001)	-0.112 (-0.161 to -0.055)	0.019 (-0.006 to 0.064)	0.166
Escherichia marmotae	-0.003 (-0.008 to 0)	-0.113 (-0.161 to -0.059)	0.025 (-0.001 to 0.074)	0.056
Citrobacter tructae	-0.001 (-0.005 to 0.003)	-0.116 (-0.159 to -0.062)	0.006 (-0.023 to 0.044)	0.646
[Ruminococcus] gnavus	-0.007 (-0.014 to -0.001)	-0.107 (-0.153 to -0.055)	0.058 (0.012 to 0.133)	0.004
Streptococcus pneumoniae	-0.004 (-0.01 to 0)	-0.112 (-0.16 to -0.062)	0.032 (-0.001 to 0.092)	0.070
Streptococcus mitis	-0.005 (-0.011 to 0)	-0.111 (-0.155 to -0.057)	0.038 (0.002 to 0.102)	0.026
Streptococcus oralis	-0.002 (-0.006 to 0.002)	-0.116 (-0.162 to -0.064)	0.011 (-0.023 to 0.056)	0.418
Streptococcus cristatus	-0.004 (-0.009 to 0)	-0.113 (-0.16 to -0.061)	0.031 (0.001 to 0.088)	0.036
Megamonas funiformis	-0.004 (-0.01 to 0)	-0.112 (-0.16 to -0.057)	0.033 (0.001 to 0.095)	0.040
Fusobacterium varium	-0.004 (-0.009 to 0)	-0.11 (-0.158 to -0.054)	0.033 (0.002 to 0.093)	0.024
Fusobacterium mortiferum	-0.005 (-0.012 to 0)	-0.109 (-0.156 to -0.054)	0.039 (0.002 to 0.114)	0.044

Supplementary Table 12 Mediating effect of gut microbiota between HLBS and elevated TG

Species	ACME	ADE	Proportion	P value of proportion
Bacteroides uniformis	-0.001 (-0.005 to 0)	-0.125 (-0.145 to -0.096)	0.01 (-0.004 to 0.037)	0.196
Bacteroides cellulosilyticus	-0.002 (-0.005 to 0)	-0.125 (-0.144 to -0.099)	0.011 (-0.003 to 0.042)	0.142
Bacteroides xylanisolvens	0 (-0.003 to 0.002)	-0.126 (-0.146 to -0.098)	0.003 (-0.013 to 0.025)	0.604
Bacteroides intestinalis	-0.002 (-0.006 to 0.001)	-0.125 (-0.145 to -0.095)	0.013 (-0.006 to 0.049)	0.198
Bacteroides zoogleoformans	0 (-0.003 to 0.001)	-0.127 (-0.146 to -0.098)	0.002 (-0.012 to 0.023)	0.682
Tannerella forsythia	0 (-0.003 to 0.003)	-0.126 (-0.146 to -0.098)	0.001 (-0.024 to 0.026)	0.860
Tannerella sp. oral taxon HOT-286	0 (-0.003 to 0.002)	-0.126 (-0.146 to -0.096)	0.001 (-0.017 to 0.021)	0.848
Paraprevotella xylaniphila	0 (-0.002 to 0.003)	-0.127 (-0.148 to -0.097)	-0.003 (-0.027 to 0.016)	0.698
Prevotella intermedia	0 (-0.003 to 0.004)	-0.128 (-0.147 to -0.097)	-0.003 (-0.037 to 0.023)	0.758
Prevotella ruminicola	0.001 (-0.002 to 0.004)	-0.128 (-0.148 to -0.1)	-0.004 (-0.035 to 0.017)	0.614
Prevotella dentalis	0 (-0.003 to 0.004)	-0.127 (-0.147 to -0.094)	-0.003 (-0.035 to 0.026)	0.824
Prevotella melaninogenica	0.001 (-0.002 to 0.004)	-0.128 (-0.147 to -0.101)	-0.004 (-0.029 to 0.013)	0.606
Prevotella fusca	0.001 (-0.001 to 0.005)	-0.128 (-0.148 to -0.099)	-0.007 (-0.037 to 0.011)	0.440
Prevotella denticola	0.001 (-0.002 to 0.004)	-0.128 (-0.147 to -0.1)	-0.004 (-0.034 to 0.018)	0.616
Prevotella oris	0 (-0.003 to 0.003)	-0.127 (-0.146 to -0.099)	-0.001 (-0.025 to 0.021)	0.886
Prevotella jejuni	0.001 (-0.002 to 0.004)	-0.128 (-0.147 to -0.098)	-0.005 (-0.034 to 0.013)	0.532
Prevotella sp. oral taxon 299	0.001 (-0.002 to 0.004)	-0.128 (-0.146 to -0.102)	-0.005 (-0.033 to 0.015)	0.550
Prevotella enoeca	0.001 (-0.003 to 0.004)	-0.127 (-0.148 to -0.099)	-0.004 (-0.036 to 0.024)	0.718
Pseudoprevotella muciniphila	0 (-0.002 to 0.003)	-0.127 (-0.147 to -0.099)	-0.003 (-0.026 to 0.017)	0.752
Butyricimonas faecalis	-0.003 (-0.007 to 0)	-0.124 (-0.144 to -0.093)	0.021 (-0.001 to 0.055)	0.062
Odoribacter splanchnicus	-0.003 (-0.007 to 0.001)	-0.124 (-0.144 to -0.092)	0.019 (-0.009 to 0.059)	0.164
Alistipes shahii	-0.004 (-0.009 to -0.001)	-0.123 (-0.143 to -0.092)	0.032 (0.007 to 0.078)	0.010
Alistipes megaguti	-0.001 (-0.004 to 0.003)	-0.127 (-0.146 to -0.101)	0.003 (-0.023 to 0.033)	0.728
Alistipes communis	-0.005 (-0.01 to -0.001)	-0.122 (-0.142 to -0.093)	0.037 (0.008 to 0.082)	0.014
Alistipes sp. dk3624	-0.003 (-0.007 to 0)	-0.125 (-0.144 to -0.096)	0.018 (-0.001 to 0.054)	0.064
Alistipes finegoldii	-0.003 (-0.007 to 0)	-0.123 (-0.143 to -0.096)	0.024 (0.003 to 0.059)	0.024
Alistipes onderdonkii	-0.003 (-0.008 to 0)	-0.123 (-0.144 to -0.094)	0.023 (0 to 0.067)	0.046
Alistipes dispar	-0.002 (-0.005 to 0)	-0.125 (-0.143 to -0.097)	0.014 (-0.002 to 0.042)	0.090
Muribaculum gordoncarteri	-0.002 (-0.006 to 0.001)	-0.125 (-0.144 to -0.098)	0.015 (-0.009 to 0.052)	0.186
Muribaculum intestinale	-0.001 (-0.005 to 0.003)	-0.126 (-0.145 to -0.098)	0.005 (-0.022 to 0.037)	0.586
Duncaniella dubosii	-0.001 (-0.004 to 0.002)	-0.126 (-0.146 to -0.1)	0.005 (-0.019 to 0.032)	0.632
Petrimonas mucosa	0.001 (-0.002 to 0.005)	-0.128 (-0.146 to -0.101)	-0.006 (-0.039 to 0.015)	0.520
Proteiniphilum saccharofermentans	0 (-0.002 to 0.003)	-0.127 (-0.147 to -0.098)	-0.002 (-0.027 to 0.016)	0.658
Barnesiella viscericola	0 (-0.004 to 0.003)	-0.127 (-0.146 to -0.098)	0.001 (-0.028 to 0.031)	0.936
Porphyromonas gingivalis	0 (-0.003 to 0.004)	-0.128 (-0.147 to -0.099)	-0.002 (-0.033 to 0.023)	0.810
Porphyromonas asaccharolytica	-0.002 (-0.006 to 0.002)	-0.126 (-0.145 to -0.098)	0.012 (-0.017 to 0.049)	0.366
Pseudomonas versuta	0 (-0.003 to 0.002)	-0.126 (-0.147 to -0.099)	0.001 (-0.017 to 0.023)	0.808
Pseudomonas taetrolens	0.002 (-0.002 to 0.006)	-0.127 (-0.147 to -0.096)	-0.01 (-0.055 to 0.017)	0.404
Pseudomonas stutzeri	-0.001 (-0.004 to 0.001)	-0.126 (-0.145 to -0.098)	0.005 (-0.007 to 0.03)	0.432
Aeromonas veronii	-0.008 (-0.016 to -0.002)	-0.118 (-0.14 to -0.088)	0.059 (0.018 to 0.131)	0.006
Escherichia coli	-0.003 (-0.007 to 0)	-0.124 (-0.144 to -0.095)	0.02 (-0.003 to 0.059)	0.092
Escherichia albertii	-0.002 (-0.006 to 0)	-0.125 (-0.146 to -0.096)	0.014 (-0.001 to 0.049)	0.080
Escherichia fergusonii	-0.001 (-0.005 to 0.001)	-0.127 (-0.146 to -0.099)	0.008 (-0.01 to 0.037)	0.346
Escherichia marmotae	-0.003 (-0.008 to 0)	-0.123 (-0.143 to -0.094)	0.024 (0.002 to 0.061)	0.028
Lelliottia sp. WB101	-0.001 (-0.004 to 0.001)	-0.125 (-0.145 to -0.098)	0.006 (-0.011 to 0.033)	0.438
Psychrobacter sp. P11G5	0 (-0.003 to 0.003)	-0.126 (-0.146 to -0.096)	0 (-0.025 to 0.024)	0.964
Alcaligenes faecalis	-0.001 (-0.005 to 0.001)	-0.125 (-0.144 to -0.096)	0.009 (-0.007 to 0.038)	0.314
Alcaligenes aquatilis	0 (-0.003 to 0.001)	-0.125 (-0.145 to -0.097)	0.002 (-0.01 to 0.022)	0.610
Comamonas kerstersii	-0.003 (-0.008 to 0)	-0.124 (-0.143 to -0.096)	0.023 (-0.003 to 0.068)	0.070
Oxalobacter formigenes	-0.001 (-0.005 to 0.001)	-0.125 (-0.144 to -0.094)	0.009 (-0.005 to 0.039)	0.234
Sutterella megalosphaeroides	-0.001 (-0.005 to 0.001)	-0.125 (-0.145 to -0.097)	0.009 (-0.004 to 0.039)	0.184
Sutterella faecalis	-0.001 (-0.005 to 0.001)	-0.126 (-0.146 to -0.097)	0.009 (-0.005 to 0.042)	0.206
Brevundimonas diminuta	-0.001 (-0.005 to 0.002)	-0.125 (-0.145 to -0.099)	0.008 (-0.015 to 0.039)	0.430
Brevundimonas naejangsanensis	-0.003 (-0.008 to 0)	-0.122 (-0.142 to -0.093)	0.025 (-0.002 to 0.065)	0.076
Desulfovibrio piger	-0.001 (-0.004 to 0.001)	-0.125 (-0.146 to -0.096)	0.007 (-0.007 to 0.037)	0.370

Species	ACME	ADE	Proportion	P value of proportion
Campylobacter jejuni	-0.001 (-0.005 to 0.001)	-0.125 (-0.144 to -0.097)	0.007 (-0.009 to 0.038)	0.348
Lachnospiraceae bacterium Choco86	-0.001 (-0.005 to 0.003)	-0.124 (-0.145 to -0.097)	0.006 (-0.024 to 0.039)	0.654
Lachnospiraceae bacterium	-0.005 (-0.011 to -0.001)	-0.119 (-0.139 to -0.092)	0.041 (0.007 to 0.09)	0.006
Lachnospiraceae bacterium KGMB03038	0 (-0.004 to 0.003)	-0.125 (-0.145 to -0.097)	0.002 (-0.024 to 0.03)	0.820
[Ruminococcus] gnavus	-0.008 (-0.015 to -0.003)	-0.118 (-0.139 to -0.091)	0.066 (0.023 to 0.124)	0.002
Blautia sp. SC05B48	0 (-0.003 to 0.003)	-0.126 (-0.147 to -0.096)	0 (-0.022 to 0.021)	0.956
Blautia argi	-0.004 (-0.01 to 0)	-0.122 (-0.142 to -0.09)	0.031 (-0.003 to 0.083)	0.070
Blautia producta	0 (-0.004 to 0.003)	-0.126 (-0.146 to -0.096)	0.001 (-0.026 to 0.034)	0.908
Anaerostipes hadrus	0.003 (0 to 0.007)	-0.129 (-0.149 to -0.102)	-0.02 (-0.059 to -0.001)	0.040
Anaerostipes rhamnosivorans	-0.001 (-0.004 to 0.003)	-0.125 (-0.145 to -0.096)	0.003 (-0.026 to 0.033)	0.740
Anaerobutyricum hallii	0 (-0.002 to 0.003)	-0.126 (-0.146 to -0.1)	-0.002 (-0.023 to 0.017)	0.764
[Clostridium] hylemonae	-0.001 (-0.005 to 0.002)	-0.124 (-0.143 to -0.093)	0.01 (-0.015 to 0.04)	0.404
[Clostridium] scindens	-0.001 (-0.006 to 0.002)	-0.125 (-0.144 to -0.095)	0.01 (-0.016 to 0.046)	0.416
Lachnoclostridium sp. YL32	0 (-0.003 to 0.003)	-0.127 (-0.146 to -0.1)	-0.001 (-0.026 to 0.022)	0.872
Lachnoclostridium phocaeense	0 (-0.004 to 0.003)	-0.125 (-0.144 to -0.094)	0.002 (-0.022 to 0.029)	0.804
Lachnoclostridium phytofermentans	0 (-0.003 to 0.003)	-0.126 (-0.146 to -0.097)	0 (-0.021 to 0.021)	0.968
Enterocloster bolteae	0 (-0.003 to 0.003)	-0.126 (-0.145 to -0.099)	-0.001 (-0.028 to 0.024)	0.886
Enterocloster clostridioformis	0.001 (-0.001 to 0.004)	-0.127 (-0.147 to -0.1)	-0.008 (-0.037 to 0.008)	0.298
Lachnospira eligens	-0.004 (-0.009 to -0.001)	-0.122 (-0.142 to -0.094)	0.029 (0.004 to 0.071)	0.020
Lacrimispora sphenoides	-0.001 (-0.005 to 0.002)	-0.125 (-0.145 to -0.098)	0.008 (-0.013 to 0.038)	0.436
Lacrimispora saccharolytica	-0.001 (-0.004 to 0.002)	-0.125 (-0.145 to -0.095)	0.005 (-0.013 to 0.03)	0.540
Anaerocolumna sedimenticola	0 (-0.003 to 0.002)	-0.125 (-0.143 to -0.097)	0.002 (-0.02 to 0.027)	0.780
Lachnoanaerobaculum umeaense	0 (-0.002 to 0.003)	-0.126 (-0.145 to -0.093)	-0.001 (-0.027 to 0.022)	0.884
Anaerotignum propionicum	0 (-0.003 to 0.003)	-0.126 (-0.146 to -0.097)	0 (-0.028 to 0.023)	0.968
Faecalibacterium prausnitzii	-0.005 (-0.01 to -0.001)	-0.123 (-0.142 to -0.096)	0.035 (0.006 to 0.077)	0.018
Ruminococcus sp. JE7A12	-0.002 (-0.005 to 0.001)	-0.125 (-0.144 to -0.096)	0.01 (-0.005 to 0.039)	0.204
Ruminococcus bicirculans	-0.003 (-0.008 to 0)	-0.123 (-0.143 to -0.093)	0.023 (0.001 to 0.065)	0.038
Ruminococcus albus	-0.004 (-0.009 to 0)	-0.123 (-0.143 to -0.095)	0.031 (0.003 to 0.073)	0.026
Ruminococcus champanellensis	-0.004 (-0.009 to 0)	-0.123 (-0.143 to -0.095)	0.028 (-0.001 to 0.074)	0.064
Dysosmobacter welbionis	-0.001 (-0.005 to 0.002)	-0.126 (-0.145 to -0.099)	0.01 (-0.017 to 0.042)	0.384
Oscillibacter sp. PEA192	-0.004 (-0.008 to 0)	-0.124 (-0.144 to -0.096)	0.027 (0.004 to 0.067)	0.030
Oscillibacter valericigenes	-0.005 (-0.01 to -0.001)	-0.122 (-0.142 to -0.095)	0.038 (0.009 to 0.083)	0.006
Caproiciproducens sp. NJN-50	-0.002 (-0.006 to 0.001)	-0.125 (-0.145 to -0.094)	0.013 (-0.009 to 0.046)	0.218
Ethanoligenens harbinense	-0.005 (-0.01 to -0.001)	-0.123 (-0.143 to -0.097)	0.036 (0.008 to 0.079)	0.006
Clostridioides difficile	0 (-0.004 to 0.004)	-0.125 (-0.145 to -0.096)	0.004 (-0.029 to 0.036)	0.776
Peptacetobacter hiranonis	-0.002 (-0.005 to 0)	-0.125 (-0.144 to -0.092)	0.011 (-0.002 to 0.04)	0.156
Massilistercora timonensis	0 (-0.003 to 0.002)	-0.126 (-0.145 to -0.098)	0.002 (-0.016 to 0.023)	0.722
Flintibacter sp. KGMB00164	-0.002 (-0.006 to 0.002)	-0.125 (-0.145 to -0.093)	0.013 (-0.017 to 0.046)	0.304
Mogibacterium diversum	-0.002 (-0.006 to 0.001)	-0.125 (-0.145 to -0.095)	0.013 (-0.007 to 0.047)	0.188
[Eubacterium] sulci	-0.001 (-0.004 to 0.003)	-0.126 (-0.145 to -0.097)	0.004 (-0.022 to 0.031)	0.708
Intestinimonas butyriciproducens	-0.003 (-0.008 to 0)	-0.123 (-0.143 to -0.096)	0.026 (-0.001 to 0.067)	0.066
Clostridium sp. DL-VIII	-0.002 (-0.006 to 0)	-0.124 (-0.144 to -0.094)	0.018 (-0.002 to 0.051)	0.088
Clostridium perfringens	-0.003 (-0.009 to 0.001)	-0.123 (-0.143 to -0.091)	0.026 (-0.008 to 0.075)	0.134
Clostridium botulinum	-0.002 (-0.005 to 0.001)	-0.125 (-0.144 to -0.097)	0.012 (-0.007 to 0.043)	0.200
Clostridium butyricum	-0.001 (-0.004 to 0.001)	-0.125 (-0.145 to -0.094)	0.008 (-0.008 to 0.037)	0.266
Clostridium pasteurianum	-0.001 (-0.005 to 0.001)	-0.125 (-0.145 to -0.096)	0.01 (-0.006 to 0.041)	0.208
Clostridium saccharoperbutylacetonicum	-0.002 (-0.006 to 0.001)	-0.125 (-0.145 to -0.095)	0.013 (-0.009 to 0.051)	0.264
Clostridium beijerinckii	-0.003 (-0.007 to 0)	-0.123 (-0.142 to -0.092)	0.022 (0 to 0.061)	0.062
Clostridium bornimense	-0.002 (-0.006 to 0.001)	-0.124 (-0.144 to -0.093)	0.015 (-0.009 to 0.053)	0.190
Clostridium baratii	-0.002 (-0.006 to 0.001)	-0.124 (-0.144 to -0.095)	0.016 (-0.008 to 0.052)	0.176
Clostridium isatidis	-0.001 (-0.005 to 0.002)	-0.125 (-0.145 to -0.097)	0.009 (-0.014 to 0.044)	0.408
Clostridium saccharobutylicum	-0.001 (-0.005 to 0.001)	-0.126 (-0.145 to -0.098)	0.009 (-0.008 to 0.039)	0.294
Clostridium chauvoei	-0.002 (-0.006 to 0.001)	-0.124 (-0.145 to -0.095)	0.013 (-0.007 to 0.05)	0.202

Species	ACME	ADE	Proportion	P value of proportion
Clostridium septicum	-0.002 (-0.006 to 0.002)	-0.125 (-0.146 to -0.095)	0.012 (-0.016 to 0.049)	0.348
Clostridium novyi	-0.001 (-0.004 to 0.001)	-0.125 (-0.146 to -0.099)	0.006 (-0.007 to 0.033)	0.372
Clostridium taeniosporum	-0.001 (-0.004 to 0.001)	-0.125 (-0.146 to -0.096)	0.008 (-0.009 to 0.035)	0.306
Hungatella hathewayi	0 (-0.003 to 0.003)	-0.124 (-0.146 to -0.094)	0.001 (-0.026 to 0.028)	0.932
Eubacterium limosum	0 (-0.004 to 0.003)	-0.126 (-0.145 to -0.096)	0.001 (-0.027 to 0.031)	0.944
Eubacterium callanderi	0.001 (-0.002 to 0.005)	-0.127 (-0.147 to -0.098)	-0.008 (-0.044 to 0.018)	0.486
Enterococcus faecium	-0.002 (-0.005 to 0)	-0.125 (-0.145 to -0.098)	0.011 (-0.003 to 0.041)	0.190
Streptococcus salivarius	-0.002 (-0.007 to 0.002)	-0.126 (-0.145 to -0.097)	0.015 (-0.018 to 0.055)	0.348
Streptococcus vestibularis	-0.003 (-0.008 to 0.002)	-0.125 (-0.144 to -0.096)	0.02 (-0.015 to 0.066)	0.252
Streptococcus sp. LPB0220	-0.005 (-0.012 to -0.001)	-0.123 (-0.143 to -0.095)	0.041 (0.007 to 0.091)	0.014
Streptococcus sp. HSISM1	-0.004 (-0.009 to 0)	-0.123 (-0.144 to -0.094)	0.029 (-0.003 to 0.07)	0.084
Streptococcus sp. A12	0 (-0.003 to 0.002)	-0.127 (-0.145 to -0.099)	0.001 (-0.019 to 0.024)	0.838
Streptococcus sp. oral taxon 431	0 (-0.003 to 0.004)	-0.126 (-0.147 to -0.097)	0 (-0.03 to 0.027)	0.990
Streptococcus sp. FDAARGOS_192	-0.002 (-0.007 to 0.003)	-0.125 (-0.145 to -0.099)	0.017 (-0.02 to 0.055)	0.316
Streptococcus pneumoniae	-0.003 (-0.008 to 0)	-0.124 (-0.144 to -0.096)	0.024 (-0.003 to 0.06)	0.080
Streptococcus dysgalactiae	0 (-0.002 to 0.003)	-0.126 (-0.146 to -0.098)	-0.001 (-0.025 to 0.018)	0.826
Streptococcus pasteurianus	0 (-0.003 to 0.003)	-0.126 (-0.146 to -0.097)	0.003 (-0.021 to 0.027)	0.704
Streptococcus thermophilus	0 (-0.003 to 0.004)	-0.128 (-0.146 to -0.099)	-0.002 (-0.03 to 0.02)	0.794
Streptococcus mitis	-0.004 (-0.009 to 0.001)	-0.123 (-0.144 to -0.092)	0.028 (-0.006 to 0.072)	0.100
Streptococcus sanguinis	0.002 (-0.001 to 0.006)	-0.128 (-0.148 to -0.099)	-0.012 (-0.049 to 0.011)	0.268
Streptococcus ilei	0.001 (-0.002 to 0.004)	-0.127 (-0.146 to -0.099)	-0.003 (-0.031 to 0.013)	0.652
Streptococcus parasanguinis	-0.005 (-0.011 to 0)	-0.122 (-0.141 to -0.094)	0.038 (0.003 to 0.084)	0.030
Streptococcus oralis	-0.001 (-0.004 to 0.002)	-0.125 (-0.145 to -0.096)	0.007 (-0.015 to 0.036)	0.488
Streptococcus cristatus	-0.004 (-0.009 to 0)	-0.122 (-0.142 to -0.092)	0.031 (0.003 to 0.072)	0.024
Streptococcus equinus	-0.001 (-0.004 to 0.002)	-0.126 (-0.146 to -0.096)	0.008 (-0.013 to 0.034)	0.422
Streptococcus australis	0 (-0.002 to 0.003)	-0.127 (-0.147 to -0.097)	-0.002 (-0.028 to 0.02)	0.780
Streptococcus koreensis	0 (-0.001 to 0.003)	-0.127 (-0.146 to -0.098)	-0.002 (-0.026 to 0.012)	0.674
Streptococcus gordonii	0 (-0.003 to 0.002)	-0.127 (-0.145 to -0.099)	0.003 (-0.017 to 0.025)	0.696
Streptococcus viridans	0.001 (-0.002 to 0.004)	-0.127 (-0.146 to -0.098)	-0.004 (-0.031 to 0.012)	0.574
Streptococcus infantarius	0.001 (-0.001 to 0.004)	-0.128 (-0.147 to -0.102)	-0.006 (-0.03 to 0.006)	0.338
Lactobacillus johnsonii	-0.002 (-0.006 to 0.001)	-0.125 (-0.144 to -0.096)	0.01 (-0.005 to 0.045)	0.216
Carnobacterium maltaromaticum	-0.001 (-0.004 to 0.001)	-0.126 (-0.146 to -0.095)	0.003 (-0.011 to 0.028)	0.554
Bacillus sp. N3536	-0.001 (-0.004 to 0.002)	-0.125 (-0.145 to -0.097)	0.005 (-0.015 to 0.032)	0.518
Psychrobacillus glaciei	0 (-0.004 to 0.003)	-0.126 (-0.144 to -0.095)	0.002 (-0.029 to 0.035)	0.812
Psychrobacillus sp. AK 1817	-0.001 (-0.004 to 0.001)	-0.125 (-0.145 to -0.097)	0.005 (-0.009 to 0.031)	0.396
Sporosarcina psychrophila	-0.001 (-0.005 to 0.002)	-0.124 (-0.145 to -0.095)	0.007 (-0.017 to 0.042)	0.476
Gemella sanguinis	-0.002 (-0.006 to 0.001)	-0.124 (-0.146 to -0.096)	0.017 (-0.006 to 0.052)	0.120
[Clostridium] innocuum	0 (-0.003 to 0.004)	-0.126 (-0.146 to -0.096)	-0.003 (-0.037 to 0.024)	0.778
Erysipelotrichaceae bacterium GAM147	0 (-0.002 to 0.001)	-0.125 (-0.145 to -0.096)	0.002 (-0.008 to 0.021)	0.598
Absiella argi	-0.003 (-0.008 to 0.001)	-0.123 (-0.143 to -0.094)	0.023 (-0.008 to 0.065)	0.150
Amedibacterium intestinale	-0.005 (-0.011 to -0.001)	-0.121 (-0.142 to -0.094)	0.04 (0.01 to 0.087)	0.004
Megamonas funiformis	-0.002 (-0.007 to 0.001)	-0.124 (-0.145 to -0.096)	0.017 (-0.006 to 0.054)	0.156
Megamonas hypermegale	-0.003 (-0.008 to 0)	-0.122 (-0.143 to -0.092)	0.026 (0.001 to 0.066)	0.030
Veillonella atypica	-0.001 (-0.003 to 0.001)	-0.125 (-0.146 to -0.098)	0.004 (-0.01 to 0.029)	0.434
Dialister massiliensis	0 (-0.002 to 0.003)	-0.127 (-0.145 to -0.101)	-0.001 (-0.025 to 0.019)	0.848
Finegoldia magna	-0.001 (-0.005 to 0.001)	-0.124 (-0.142 to -0.093)	0.01 (-0.009 to 0.039)	0.292
Bifidobacterium breve	-0.001 (-0.004 to 0.002)	-0.126 (-0.145 to -0.099)	0.003 (-0.02 to 0.029)	0.732
Bifidobacterium pseudocatenulatum	0 (-0.002 to 0.003)	-0.127 (-0.147 to -0.097)	0 (-0.025 to 0.02)	0.944
Bifidobacterium dentium	0 (-0.002 to 0.001)	-0.126 (-0.146 to -0.099)	0.001 (-0.011 to 0.018)	0.718
Actinomyces sp. oral taxon 414	-0.001 (-0.005 to 0.001)	-0.125 (-0.145 to -0.095)	0.009 (-0.006 to 0.036)	0.256
Schaalia odontolytica	-0.003 (-0.007 to 0)	-0.123 (-0.144 to -0.093)	0.022 (-0.002 to 0.061)	0.078
Rhodococcus erythropolis	-0.001 (-0.004 to 0.001)	-0.125 (-0.145 to -0.096)	0.006 (-0.007 to 0.03)	0.304
Microbacterium oxydans	-0.001 (-0.004 to 0.001)	-0.125 (-0.146 to -0.098)	0.006 (-0.006 to 0.03)	0.330
Arthrobacter sp. YC-RL1	-0.001 (-0.005 to 0.001)	-0.125 (-0.145 to -0.098)	0.008 (-0.005 to 0.038)	0.232
Rothia mucilaginosa	0 (-0.003 to 0.002)	-0.126 (-0.148 to -0.095)	0.002 (-0.016 to 0.022)	0.702

Species	ACME	ADE	Proportion	P value of proportion
Oerskovia sp. KBS0722	-0.001 (-0.005 to 0.001)	-0.125 (-0.146 to -0.098)	0.007 (-0.008 to 0.036)	0.336
Gordonibacter urolithinfaciens	-0.001 (-0.006 to 0.004)	-0.125 (-0.146 to -0.097)	0.008 (-0.03 to 0.049)	0.606
Gordonibacter pamelaeae	-0.002 (-0.005 to 0.002)	-0.126 (-0.146 to -0.096)	0.01 (-0.012 to 0.044)	0.328
Arabia massiliensis	-0.004 (-0.009 to 0.001)	-0.123 (-0.143 to -0.094)	0.027 (-0.007 to 0.075)	0.118
Adlercreutzia sp. 8CFCBH1	-0.002 (-0.005 to 0)	-0.125 (-0.144 to -0.098)	0.013 (-0.004 to 0.044)	0.158
Adlercreutzia equolifaciens	-0.002 (-0.006 to 0.001)	-0.125 (-0.145 to -0.096)	0.01 (-0.004 to 0.043)	0.172
Olsenella sp. GAM18	-0.001 (-0.004 to 0.002)	-0.126 (-0.145 to -0.099)	0.005 (-0.017 to 0.034)	0.546
Akkermansia muciniphila	-0.001 (-0.005 to 0.001)	-0.125 (-0.145 to -0.096)	0.009 (-0.009 to 0.042)	0.316
Fusobacterium varium	-0.004 (-0.01 to 0)	-0.122 (-0.142 to -0.097)	0.031 (-0.001 to 0.073)	0.060
Fusobacterium ulcerans	-0.002 (-0.006 to 0)	-0.124 (-0.145 to -0.095)	0.016 (-0.002 to 0.049)	0.076
Fusobacterium nucleatum	-0.002 (-0.006 to 0)	-0.124 (-0.143 to -0.094)	0.017 (-0.004 to 0.056)	0.110
Fusobacterium mortiferum	-0.004 (-0.009 to 0)	-0.123 (-0.143 to -0.096)	0.027 (-0.001 to 0.072)	0.056

Supplementary Table 13 Differential metabolites between dyslipidemia and control group

Metabolites	FC	log2(FC)	P value	-LOG10(p)
13-L-Hydroperoxylinoleic acid	2.0021	1.0015	0.0100539	1.9976644
7-Sulfocholic acid	0.4181	-1.2581	1.80E-05	4.7445643
Adipic acid	0.3649	-1.4546	0.0069705	2.1567355
Busulfan	2.0391	1.028	0.0202856	1.6928117
dTDP-D-glucose	2.052	1.0371	0.026541	1.576083
Genipin	2.435	1.2839	0.0097914	2.0091569
L-Arogenate	2.0774	1.0547	0.0247679	1.6061111
N4-Acetylcytidine	0.325	-1.6214	0.0374136	1.42697
NCGC00381071-01!1,12-dihydroxy-1,6,12,17,23,28-hexazacyclotritriacontane-	2.0347	1.0248	0.0016698	2.7773418
NP-016455	2.2916	1.1963	0.0375936	1.4248863

Supplementary Table 14 Differential metabolites between decreased HDL and control group

Metabolites	FC	log2(FC)	P value	-LOG10(p)
(2S,3S,4S,8R,9S,13R,14R,15R,16R)-3,4,8,14,15-pentahydroxy-2,13,16-trimethyl-6-methylidene-10-oxatetracyclo[7.6.1.02,7.012,16]hexadecan-11-	0.2398	-2.0603	0.0011998	2.9208804
(3R,4S)-4,6,8-Trihydroxy-7-methoxy-3-methyl-3,4-dihydro-1H-isochromen-1-one	0.399	-1.3255	0.0352213	1.4531945
(S)-[10]-Gingerol	2.6365	1.3986	0.0005386	3.2687528
2,6-Diamino-5-hydroxyhexanoic acid hydrochloride	0.3738	-1.4196	0.0354388	1.4505213
3,3',4,4',5,5'-hexabromo-1H,1'H-2,2'-bipyrrole	0.4105	-1.2847	0.0180792	1.7428216
4-Hydroxybenzoic acid	0.4909	-1.0266	0.0017203	2.7643901
4-Hydroxytamoxifen	0.3227	-1.6319	0.0289038	1.5390454
7-Sulfocholic acid	0.3821	-1.3881	0.0002053	3.6875881
8-Isoprostane	0.3185	-1.6506	0.0092689	2.0329735
bk-EABDI	0.2189	-2.192	0.0021161	2.6744663
Caryophyllene [T(-)]	2.1344	1.0939	0.0053386	2.2725764
Cortexolone	2.2505	1.1703	0.0128599	1.8907624
Corydaline	0.477	-1.0681	0.0211043	1.6756283
Diphenhydramine	0.3896	-1.3599	0.0005236	3.2810415
Dodecanedioic acid	0.3069	-1.7041	0.0407384	1.3899964
Geranic acid	2.1631	1.1131	0.0232895	1.6328398
Gibberellin A8	0.4267	-1.2288	0.0165355	1.7815836
Glycyrrhetinic acid	0.4628	-1.1116	0.0197934	1.7034802
Histamine	2.2293	1.1566	0.0014886	2.8272188
L-Ascorbic acid, 6-octadecanoate	2.1275	1.0892	0.0117094	1.9314647
Levorphanol	0.4507	-1.1499	0.0228714	1.6407072
Lipoate	0.4001	-1.3215	0.0253703	1.5956749
NCGC00180087-02!5-(hydroxymethyl)-3-(1-hydroxy-4-methylhexyl)oxolan-2-one	0.3236	-1.6279	0.0018505	2.7327134
NCGC00380117-01_C27H41NO4_(7E)-3-Isobutyl-4,5,8,12,12-pentamethyl-3,3a,4,6a,9,10,10a,13a,14,15-decahydro-1H-[1,3]dioxolo[7,8]cycloundeca[1,2-d]isoindole-1,16(2H)-dione	2.0304	1.0218	0.0117644	1.9294292
NCGC00381071-01!1,12-dihydroxy-1,6,12,17,23,28-hexazacyclotritriacontane-2,5,13,16,24,27-hexone	3.0595	1.6133	0.0118952	1.9246279
NCGC00381248-01!4-oxododecanedioic acid	0.4679	-1.0957	0.0337766	1.4713837
NP-013808	0.2927	-1.7724	0.0052694	2.2782397
NP-016596	0.4876	-1.0363	0.0448753	1.3479926
Poly THF n5	2.4529	1.2945	0.0013072	2.8836685
Polylimonene	2.4548	1.2956	0.0350042	1.4558795
Prazosin	0.4899	-1.0294	0.0050293	2.2984901
Protocatechuic acid	0.4552	-1.1354	0.0482204	1.3167695
Shikimic acid	0.468	-1.0954	0.0169005	1.7721015
Traumatic Acid	0.4093	-1.2886	0.0490525	1.3093388
Trehalose dihydrate	0.3745	-1.4171	0.008003	2.096748

Supplementary Table 15 Differential metabolites between elevated LDL and control group

Metabolites	FC	log2(FC)	P value	-LOG10(p)
2-Deacetoxy taxinine B	2.039	1.0278	0.0302997	1.518562
21-Deoxycortisol	2.1126	1.079	0.0365431	1.4371948
3-Hydroxybenzoic acid	4.0136	2.0049	3.84E-05	4.4155596
Adipic acid	0.2939	-1.7668	0.0187645	1.7266635
Asperuloside	2.0471	1.0336	0.0023654	2.6261013
Beta-Tyrosine	0.4961	-1.0114	0.014259	1.8459112
Busulfan	2.4252	1.2781	0.0004238	3.3728591
Enterodiol	2.1182	1.0828	0.0303993	1.5171363
Equol	2.3884	1.256	0.0498112	1.3026727
Ethylmorphine	0.181	-2.466	0.0198748	1.7016974
Gallic acid	2.6352	1.3979	0.0001237	3.9075975
Genipin	2.123	1.0861	0.0016923	2.7715258
Glutamine	0.4025	-1.3129	0.0242869	1.6146275
N-(4-isopropylphenyl)-2-{[4-(3-methoxypropyl)-5-(2-methyl-1,3-thiazol-4-yl)-4H-1,2,4-triazol-3-yl]thio}acetamide	0.4971	-1.0083	0.0125695	1.9006823
NCGC00381380-01!(2R)-3-hydroxy-2-[(2-hydroxybenzoyl)amino]propanoic acid	2.582	1.3685	0.0232138	1.6342546
Nevskin	0.2269	-2.1401	0.0370879	1.4307677
NP-012268	0.2835	-1.8184	0.0318945	1.4962846
NP-016455	2.855	1.5135	0.0112267	1.9497474
NP-017667	2.2872	1.1936	0.0023159	2.6352745
Octabenzone	0.4323	-1.2098	0.0128575	1.8908439
Quinate	2.9668	1.5689	0.0475714	1.3226539
Vanillylmandelic acid	5.6916	2.5088	0.0079212	2.1012104

Supplementary Table 16 Differential metabolites between elevated TC and control group

Metabolites	FC	log2(FC)	P value	-LOG10(p)
(2E)-N-(4-acetamidobutyl)-3-(4-hydroxy-3-methoxyphenyl)prop-2-enamide	0.4806	-1.0572	0.0047165	2.3263811
(2S,5S)-trans-Carboxymethylproline	3.8751	1.9542	0.0433898	1.3626121
(R)-Laudanidine	0.3584	-1.4802	0.0357932	1.4461998
2-[6-(diethylamino)purin-9-yl]-5-(hydroxymethyl)oxolane-3,4-diol	0.3781	-1.4032	0.0018822	2.725329
2-Deacetoxy taxinine B	2.1463	1.1019	0.011218	1.9500834
2-Methyl-3-hydroxy-5-formylpyridine-4-carboxylate	2.5095	1.3274	0.0066393	2.1778775
3-Hydroxybenzoic acid	4.7758	2.2558	9.35E-05	4.0290994
3-Hydroxymethylglutaric acid	2.3852	1.2541	0.0130693	1.8837462
3,5,7,15-tetraacetoxy-2-hydroxy-8-isobutyroyloxy-9,14-dioxojatropha-6(17),11E-diene (2)	2.0917	1.0647	0.010475	1.9798443
4,4-Bis(4-hydroxyphenyl)heptane	2.1646	1.1141	0.0357355	1.4468996
6-Hydroxynicotinic acid	2.1805	1.1246	0.0038389	2.4157891
7-Ketocholesterol	0.4791	-1.0617	0.0165275	1.7817933
7-Sulfocholic acid	0.4135	-1.2742	0.031119	1.5069748
Adipic acid	0.2449	-2.0298	0.0217562	1.6624163
Asperuloside	2.9155	1.5438	0.0002394	3.6208502
Beta-Tyrosine	0.3506	-1.5121	0.0018107	2.7421463
Bioresmethrin	0.4076	-1.2948	0.0182812	1.7379956
Busulfan	2.9091	1.5406	0.0001291	3.889001
CAY10498	0.4494	-1.1539	0.0137126	1.8628811
Citalopram	0.3594	-1.4762	0.008588	2.0661087
Citrinin	2.3044	1.2044	0.0036491	2.4378164
Dipropyleneglycol dibenzoate	0.4705	-1.0877	0.0097355	2.0116428
Ecgonine	0.3413	-1.5508	0.0312857	1.5046541
Enterodiol	2.1214	1.085	0.0343043	1.4646509
Galacturonic acid	2.1546	1.1074	0.0025058	2.6010545
Gallic acid	3.4151	1.7719	1.03E-05	4.9891051
Ganoderic acid G	2.2176	1.149	0.0120495	1.9190302
Genipin	2.6879	1.4265	0.0001972	3.7050502
Glutamine	0.3	-1.7371	0.0169775	1.7701258
Indinavir	0.414	-1.2722	0.0015629	2.8060706
L-Arogenate	2.1778	1.1229	0.0049066	2.3092234
Lopinavir	0.2982	-1.7458	0.003964	2.4018635
N-(4-isopropylphenyl)-2-{[4-(3-methoxypropyl)-5-(2-methyl-1,3-thiazol-4-yl)-4H-1,2,4-triazol-3-yl]thio}acetamide	0.4281	-1.2238	0.0089869	2.0463891
N1-Benzyl-2-[(2-oxo-3-piperidyl)carbonyl]hydrazine-1-carbothioamide	2.0975	1.0687	0.0071115	2.1480369
NCGC00380117-01_C27H41NO4_(7E)-3-Isobutyl-4,5,8,12,12-pentamethyl-3,3a,4,6a,9,10,10a,13a,14,15-decahydro-1H-[1,3]dioxolo[7,8]cycloundeca[1,2-d]isoindole-	0.464	-1.1078	0.0164902	1.7827748
NCGC00381061-01_C30H51N5O9_Pyrrolo[1,2-d][1,4,7,10,13,16]oxapentaazacyclononadecine-1,4,7,10,14,17(11H,16H)-hexone, 16-(2,3-dihydroxypropyl)dodecahydro-	0.3083	-1.6975	0.0169398	1.7710915
NCGC00381220-01!16-butan-2-yl-3-(2,3-dihydroxypropyl)-10,11,14-trimethyl-13-propan-2-yl-4-oxa-1,8,11,14,17-pentazabicyclo[17.3.0]docosane-2,5,9,12,15,18-hexone	0.3145	-1.6689	0.0363228	1.4398206
NCGC00381380-01!(2R)-3-hydroxy-2-[(2-hydroxybenzoyl)amino]propanoic acid	3.3411	1.7403	0.0037271	2.4286241
NCGC00384635-01_C27H34O8_Methyl [(1S,3S,7R,8R,9R,12S,13S)-13-(3-furyl)-6,6,8,12-tetramethyl-17-methylene-5,15-dioxo-2,14-dioxatetracyclo[7.7.1.0~1,12~.0~3,8~]heptadec-7-yl](hydroxy)acetate	2.38	1.251	0.0338108	1.4709447
Neamine	0.4025	-1.3128	0.0142049	1.8475612
NP-012268	0.2649	-1.9168	0.0302573	1.5191694
NP-016455	3.5367	1.8224	0.0014743	2.8314096
NP-017667	2.8353	1.5035	0.0010607	2.9744107
Octabenzone	0.2833	-1.8195	0.0032386	2.4896373
Pipecolic acid	0.4955	-1.013	0.0217367	1.6628064
Pleiomutinine	0.3731	-1.4222	0.0249432	1.6030475

Metabolites	FC	log2(FC)	P value	-LOG10(p)
Probucol	2.0217	1.0155	0.0093327	2.029993
Quinate	3.8227	1.9346	0.0042295	2.373713
Strychnopentamine	0.3188	-1.6491	0.0441144	1.3554195
Thalsimine	2.6225	1.3909	0.0064127	2.1929558
Tranexamic Acid	0.4787	-1.0629	0.0488916	1.3107656
Vanillylmandelic acid	6.9305	2.793	0.002593	2.5861973

Supplementary Table 17 Differential metabolites between elevated TG and control group

Metabolites	FC	log2(FC)	P value	-LOG10(p)
(2E)-N-(4-acetamidobutyl)-3-(4-hydroxy-3-methoxyphenyl)prop-2-enamide	0.3747	-1.4163	0.0044349	2.3531146
1-palmitoylglycerophosphocholine	2.0561	1.0399	0.0162339	1.7895778
13-L-Hydroperoxylinoleic acid	3.3601	1.7485	0.001666	2.7783377
2-[6-(diethylamino)purin-9-yl]-5-(hydroxymethyl)oxolane-3,4-diol	0.4284	-1.223	0.0090271	2.0444514
20-HETE	2.4625	1.3001	0.045683	1.3402451
3,4-Dihydroxybenzeneacetic acid	2.071	1.0503	0.0121873	1.9140914
3alpha,12alpha-Dihydroxy-5beta-chol-6-enoate	2.2668	1.1807	0.0015681	2.8046281
7-Sulfocholic acid	0.4536	-1.1404	0.0108691	1.9638049
9(S)-HPODE	2.343	1.2283	0.00926	2.0333868
Beta-Tyrosine	0.4617	-1.1151	0.0135703	1.8674119
Bioresmethrin	0.404	-1.3078	0.0043337	2.3631443
Boc-Phe(NMe)-Pro-Phe(NMe)-Gly-OMe	0.488	-1.0352	0.049504	1.3053597
Busulfan	2.3379	1.2252	0.0470421	1.3275131
CAY10498	0.3327	-1.5878	0.0009774	3.0099282
Citalopram	0.483	-1.05	0.0070063	2.1545108
Cortisone acetate	0.3303	-1.5983	0.0266365	1.5745235
D-Maltose	2.0349	1.0249	0.0006789	3.1682027
DIHYDROCELASTRYL DIACETATE	2.3437	1.2288	8.71E-05	4.0601867
Dipropyleneglycol dibenzoate	0.4496	-1.1531	0.0162416	1.7893723
Dodecanedioate	0.4438	-1.172	0.0471122	1.326867
dTDP-D-glucose	2.172	1.119	0.0465175	1.3323832
Ecgonine	0.2925	-1.7734	0.0446987	1.3497054
Gallic acid	2.1871	1.129	0.0068463	2.1645453
Genipin	4.0586	2.021	0.003944	2.4040651
Glycocholic acid	2.0064	1.0046	0.0075183	2.123883
Histamine	2.0516	1.0367	0.0009889	3.0048331
Indinavir	0.4844	-1.0458	0.0349642	1.4563766
L-Arogenate	3.3136	1.7284	0.0045679	2.340285
L-Carnitine	2.3127	1.2096	0.0275718	1.5595345
Lipoxin A4	2.3509	1.2332	0.0328505	1.483458
N2,N5-Dibenzylpyrrolidine-2,5-dicarboxamide	0.4746	-1.0752	0.004192	2.3775786
N4-Acetylcytidine	0.2676	-1.9017	0.0427844	1.3687145
NCGC00381071-01!1,12-dihydroxy-1,6,12,17,23,28-hexazacyclotritriacontane-2,5,13,16,24,27-hexone	2.2025	1.1391	0.0230497	1.637335
Neamine	0.398	-1.3291	0.0210572	1.6765985
NP-017667	2.2306	1.1575	0.034659	1.4601834
Octabenzone	0.246	-2.0231	0.0040966	2.3875722
PC(18_0e_20-HDoHE)	2.0459	1.0327	0.0305562	1.5149007
PGF2alpha diethyl amide	2.8456	1.5087	0.0391183	1.40762
Porphobilinogen	2.7383	1.4533	0.0001388	3.8575627
Serine-Cholic Acid	2.3485	1.2317	0.0251414	1.5996102
Shikimic acid	0.4847	-1.0449	0.0174405	1.7584417
Strychnopentamine	0.351	-1.5105	0.0458361	1.3387925
Taurohyocholate	2.2207	1.151	0.0084037	2.075532
Ursolic acid	2.4404	1.2871	0.039315	1.405442
vanillylmandelate	0.3748	-1.4157	0.0012672	2.8971536
Vanillylmandelic acid	4.9974	2.3212	0.0457152	1.3399394

Supplementary Table 18 Sensitivity analysis (exclusion of who received anti-hyperlipidemia therapy) Differential gut microbiota between dyslipidemia and control group

Species	coef	stderr	pval	qval
Bacteroides intestinalis	-0.433713397	0.172767803	0.012226263	0.156224466
Aeromonas veronii	-0.345194881	0.096059294	0.000344236	0.039587106
Escherichia coli	0.616986335	0.219067961	0.004987545	0.137009284
Escherichia albertii	0.406594192	0.15429104	0.008553999	0.137673869
Escherichia marmotae	0.424560646	0.151697258	0.005240153	0.137009284
Psychrobacter sp. P11G5	-0.490887857	0.207679492	0.018295473	0.191270857
Haemophilus parainfluenzae	-0.634136603	0.224675071	0.004866284	0.137009284
Brevundimonas naejangsanensis	-0.406788809	0.170002173	0.016938159	0.182614522
Lachnospiraceae bacterium Choco86	0.242874639	0.077728988	0.001834424	0.092251748
Lachnospiraceae bacterium	0.345468232	0.088869018	0.000108371	0.018694046
Lachnospiraceae bacterium KGMB03038	0.188776644	0.071363197	0.008297197	0.137673869
[Ruminococcus] gnavus	0.595225768	0.127489423	3.47E-06	0.001196637
Blautia argi	0.287832906	0.090376745	0.001508326	0.092251748
Anaerostipes rhamnosivorans	0.170344479	0.076146706	0.025621298	0.212752536
[Clostridium] hylemonae	0.207924197	0.073375231	0.004698906	0.137009284
[Clostridium] scindens	0.20634929	0.077150519	0.007610205	0.137673869
Lachnoclostridium phocaeense	0.170509608	0.069549239	0.014400355	0.171314568
Enterocloster bolteae	0.249472176	0.110595068	0.024331096	0.209855707
Lachnospira eligens	-0.347273565	0.123202731	0.004922288	0.137009284
Lacrimispora sphenoides	0.162114995	0.07520659	0.031365828	0.240471349
Anaerotignum propionicum	0.178672001	0.070746723	0.011722048	0.155542563
Ruminococcus bicirculans	-0.465246356	0.178208065	0.009178258	0.137673869
Ruminococcus albus	-0.205630835	0.092621865	0.02664662	0.212752536
Clostridioides difficile	0.142138892	0.063812023	0.026150632	0.212752536
Hungatella hathewayi	0.180572317	0.079867547	0.024084753	0.209855707
Eubacterium limosum	0.23351218	0.094221971	0.013433684	0.165522179
Streptococcus vestibularis	0.397296626	0.162849977	0.014934673	0.171748738
Streptococcus sp. LPB0220	0.43478139	0.167931383	0.009772306	0.139165629
Streptococcus sp. HSISM1	0.422772831	0.163985231	0.010084466	0.139165629
Streptococcus pneumoniae	0.23814392	0.088358302	0.007160728	0.137673869
Streptococcus pasteurianus	0.287202407	0.127105092	0.02407445	0.209855707
Streptococcus mitis	0.378429166	0.119322755	0.00156562	0.092251748
Streptococcus parasanguinis	0.420260089	0.160433128	0.00894616	0.137673869
Ligilactobacillus ruminis	0.50533411	0.165431267	0.002316322	0.099891382
Sporosarcina psychrophila	-0.35648987	0.147985832	0.016188245	0.180159496
Absiella argi	0.223868712	0.098775524	0.023648696	0.209855707
Faecalitalea cylindroides	0.207798893	0.09010775	0.021448136	0.205544638
Amedibacterium intestinale	0.362052588	0.130277374	0.005559797	0.137009284
Megamonas funiformis	0.743061198	0.321792136	0.021153382	0.205544638
Megamonas hypermegale	0.422775783	0.155140493	0.006546036	0.137673869
Finegoldia magna	0.241494996	0.077409862	0.001871775	0.092251748
Bifidobacterium breve	0.294235755	0.132956957	0.027133657	0.212752536
Bifidobacterium catenulatum	0.558159663	0.213626233	0.009139474	0.137673869
Fusobacterium varium	0.421220441	0.158238622	0.007900644	0.137673869
Fusobacterium nucleatum	0.189430457	0.080757162	0.01919966	0.194820077

Supplementary Table 19 Sensitivity analysis (exclusion of who received anti-hyperlipidemia therapy) Differential gut microbiota between decreased HDL and control group

Species	coef	stderr	pval	qval
Bacteroides uniformis	-0.633008467	0.275732777	0.021969667	0.168434111
Bacteroides intestinalis	-0.50823596	0.240634987	0.03501694	0.209497319
Phocaeicola vulgatus	-0.572715587	0.259430732	0.027577601	0.190670654
Prevotella ruminicola	0.568416527	0.266657105	0.033760723	0.209497319
Prevotella melaninogenica	0.537520616	0.254566734	0.0350642	0.209497319
Prevotella fusca	0.562160511	0.257179855	0.029138171	0.197111155
Prevotella scopos	0.491794188	0.241148939	0.041766415	0.240156886
Enterobacter cloacae	-0.482326618	0.211449946	0.022828766	0.171215744
Enterobacter kobei	-0.783542694	0.272599841	0.004323222	0.059660463
Enterobacter roggenkampii	-1.291163577	0.309822478	3.64E-05	0.002094101
Klebsiella pneumoniae	-1.006842147	0.303122688	0.000938939	0.027166651
Klebsiella oxytoca	-0.481069584	0.20341838	0.018307961	0.146889451
Klebsiella quasipneumoniae	-0.802904812	0.2656226	0.00259352	0.047825959
Klebsiella aerogenes	-0.576487808	0.20999588	0.006194115	0.076168468
Klebsiella sp. FDAARGOS_511	-0.645884037	0.236176674	0.006402071	0.076168468
Klebsiella variicola	-0.950391214	0.314228006	0.002577103	0.047825959
Raoultella ornithinolytica	-1.030925926	0.312314824	0.001023671	0.027166651
Raoultella planticola	-0.847296359	0.254327001	0.00094604	0.027166651
Yersinia enterocolitica	-0.331290925	0.155990244	0.034081399	0.209497319
Acinetobacter johnsonii	-0.439946661	0.208536233	0.035219839	0.209497319
Psychrobacter sp. P11G5	-0.596222378	0.276009043	0.031079526	0.206200701
Haemophilus parainfluenzae	-0.889851886	0.305764112	0.003720046	0.05807121
Lachnospiraceae bacterium Choco86	0.316883932	0.109751783	0.004039736	0.05807121
Lachnospiraceae bacterium KM106-2	0.251041936	0.112170622	0.025543898	0.183596768
Lachnospira eligens	-0.50808612	0.167210011	0.002463329	0.047825959
Dysosmobacter welbionis	0.39478002	0.145016648	0.006634541	0.076297226
Oscillibacter sp. PEA192	0.424718212	0.182788459	0.020418561	0.160100083
Oscillibacter valericigenes	0.563260641	0.180552629	0.001880584	0.043253426
Caproiciproducens sp. NJN-50	0.276217953	0.116219789	0.01772123	0.145567249
Ethanoligenens harbinense	0.387134994	0.132174419	0.003505161	0.057584789
Flintibacter sp. KGMB00164	0.334855333	0.137039832	0.014777736	0.127457969
Intestinimonas butyriciproducens	0.480680034	0.179339844	0.007518962	0.083678765
Clostridium sp. SY8519	0.276974862	0.112836787	0.014339726	0.126851421
Clostridiales bacterium CCNA10	0.347455222	0.134973615	0.010238552	0.103891188
Christensenella sp. Marseille-P3954	0.3598736	0.12247347	0.003401471	0.057584789
Enterococcus avium	-0.586925229	0.235685266	0.012980933	0.120035539
Enterococcus casseliflavus	-0.664919046	0.273387126	0.015244268	0.128274936
Streptococcus ilei	-0.616499691	0.21326006	0.004004978	0.05807121
Streptococcus koreensis	-0.680724447	0.215933886	0.001698994	0.041868059
Streptococcus viridans	-0.428639164	0.194157056	0.027633428	0.190670654
Lactococcus lactis	0.404247562	0.162759607	0.013221306	0.120035539
Lactococcus cremoris	0.479953532	0.129551005	0.000227278	0.011201559
Ligilactobacillus ruminis	1.559022332	0.237047123	9.06E-11	3.12E-08
Leuconostoc mesenteroides	0.427600345	0.167763762	0.011049697	0.108918444
Fructilactobacillus sanfranciscensis	0.431889449	0.163888226	0.008582299	0.089724038
Faecalitalea cylindroides	0.444022547	0.12847273	0.000596393	0.025719436
Intestinibaculum porci	0.371740522	0.112056966	0.000952701	0.027166651
Megamonas funiformis	0.90134321	0.437182765	0.039582359	0.231456166

Species	coef	stderr	pval	qval
Megamonas hypermegale	0.475526664	0.209969633	0.023815521	0.174816055
Dialister massiliensis	1.020233032	0.362394675	0.005002747	0.066382603
Dialister hominis	1.116059082	0.369837344	0.002633893	0.047825959
Bifidobacterium longum	0.791674353	0.288468904	0.006402567	0.076168468
Bifidobacterium adolescentis	1.577207711	0.337886638	3.61E-06	0.000311415
Bifidobacterium breve	0.486665593	0.18302224	0.008004595	0.086299541
Bifidobacterium catenulatum	1.692612819	0.292123556	1.24E-08	2.14E-06
Bifidobacterium bifidum	1.298644414	0.272223682	2.21E-06	0.000254372
Bifidobacterium angulatum	1.048927312	0.227039908	4.52E-06	0.000312033
Bifidobacterium pseudocatenulatum	1.346359988	0.396555203	0.000724398	0.027166651
Olsenella sp. GAM18	0.446024163	0.178109335	0.012485266	0.119650469
Cloacibacillus porcorum	0.365328638	0.173162573	0.035214791	0.209497319

Supplementary Table 20 Sensitivity analysis (exclusion of who received anti-hyperlipidemia therapy) Differential gut microbiota between elevated LDL and control group

Species	coef	stderr	pval	qval
[Ruminococcus] gnavus	0.70063971	0.195377815	0.000359214	0.123928697

Supplementary Table 21 Sensitivity analysis (exclusion of who received anti-hyperlipidemia therapy) Differential gut microbiota between elevated TC and control group

Species	coef	stderr	pval	qval
Escherichia coli	1.127435319	0.348043455	0.001255399	0.108278139
Escherichia albertii	0.681035503	0.247824458	0.006151437	0.176853817
Escherichia fergusonii	0.737118898	0.265331448	0.005617538	0.176853817
Escherichia marmotae	0.691503156	0.241259013	0.00428058	0.176853817
Citrobacter tructae	0.835165388	0.289070167	0.003990587	0.176853817
[Ruminococcus] gnavus	0.805351994	0.197254848	4.97E-05	0.017143233
Ruminococcus bicirculans	-0.760333043	0.292374317	0.009505887	0.234252222
Streptococcus pneumoniae	0.465712849	0.137300457	0.000733407	0.108278139
Streptococcus mitis	0.620443034	0.18809296	0.001021613	0.108278139
Streptococcus oralis	0.498306777	0.184034568	0.006942807	0.18425142
Streptococcus cristatus	0.501053441	0.164344074	0.002384947	0.137134466
Megamonas funiformis	1.423186197	0.513804673	0.005757293	0.176853817
Fusobacterium varium	0.688336545	0.246512796	0.005377756	0.176853817
Fusobacterium mortiferum	1.100441838	0.350060449	0.001743041	0.120269847

Supplementary Table 22 Sensitivity analysis (exclusion of who received anti-hyperlipidemia therapy) Differential gut microbiota between elevated TG and control group

Species	coef	stderr	pval	qval
Bacteroides uniformis	-0.459152619	0.257205519	0.074627972	0.170507617
Bacteroides cellulosilyticus	-0.407850557	0.221487319	0.06594361	0.160862687
Bacteroides intestinalis	-0.570620727	0.222351861	0.010468508	0.043513677
Bacteroides zoogleoformans	-0.306126191	0.198455539	0.123566368	0.244739138
Tannerella forsythia	-0.454724136	0.175677167	0.009824008	0.041842996
Tannerella sp. oral taxon HOT-286	-0.38865579	0.189586738	0.04070075	0.117014656
Paraprevotella xylaniphila	-0.467378587	0.22674138	0.039857153	0.11555225
Prevotella intermedia	-0.483250101	0.197430508	0.0145981	0.054154244
Prevotella ruminicola	-0.454852618	0.249156522	0.06880587	0.163943339
Prevotella dentalis	-0.677796987	0.246541608	0.006112812	0.028889317
Prevotella melaninogenica	-0.39826035	0.237643381	0.094166229	0.201784777
Prevotella fusca	-0.406823954	0.242013365	0.093558798	0.201736158
Prevotella denticola	-0.519962834	0.234164142	0.026672916	0.085205148
Prevotella oris	-0.534070349	0.254717814	0.03667827	0.110034811
Prevotella jejuni	-0.356117046	0.214742257	0.097910745	0.205970774
Prevotella sp. oral taxon 299	-0.517316424	0.22236467	0.02025297	0.07057853
Prevotella enoeca	-0.679188248	0.232624113	0.003605488	0.019136823
Pseudoprevotella muciniphila	-0.384925395	0.191038301	0.044258735	0.121184631
Butyricimonas faecalis	-0.680842434	0.213268974	0.001480469	0.011350266
Odoribacter splanchnicus	-0.832677142	0.229980395	0.000314331	0.003873013
Alistipes shahii	-1.002175294	0.282051566	0.000403715	0.00464272
Alistipes megaguti	-0.816672257	0.253051459	0.001385795	0.010865894
Alistipes communis	-0.905997921	0.244592686	0.000227227	0.003266395
Alistipes sp. dk3624	-0.595642835	0.228090302	0.009191697	0.039997316
Alistipes finegoldii	-0.694998519	0.231437282	0.002759906	0.017002908
Alistipes onderdonkii	-0.943684589	0.289458851	0.00116242	0.009781339
Alistipes dispar	-0.596853855	0.233578398	0.010801319	0.043840648
Muribaculum gordoncarteri	-0.630304934	0.181885618	0.000558582	0.00583972
Muribaculum intestinale	-0.560027111	0.175552792	0.001550207	0.011469306
Duncaniella dubosii	-0.474913695	0.170529401	0.005535103	0.027280148
Petrimonas mucosa	-0.415445575	0.18104811	0.022019133	0.074476479
Proteiniphilum saccharofermentans	-0.309605513	0.178137868	0.083071449	0.186101622
Barnesiella viscericola	-0.61148735	0.206216672	0.003120049	0.017886516
Porphyromonas gingivalis	-0.477571859	0.169735059	0.005085204	0.025799933
Porphyromonas asaccharolytica	-0.778393282	0.209255091	0.000221597	0.003266395
Pseudomonas versuta	-0.587903321	0.323886343	0.069887802	0.164022393
Pseudomonas taetrolens	-0.7635395	0.34073244	0.025318866	0.082405742
Pseudomonas stutzeri	-0.263894386	0.167386758	0.115308407	0.235392901
Aeromonas veronii	-0.514411862	0.127837741	6.35E-05	0.001681013
Escherichia coli	1.097540172	0.278484098	9.04E-05	0.001707394
Escherichia albertii	0.685413467	0.196504421	0.00051835	0.005588459
Escherichia fergusonii	0.681254835	0.209776878	0.001239995	0.009948797
Escherichia marmotae	0.758505108	0.192210345	8.75E-05	0.001707394
Salmonella enterica	0.27968769	0.178428404	0.117445658	0.235574141
Lelliottia sp. WB101	-0.34510361	0.189390083	0.068903722	0.163943339
Psychrobacter sp. P11G5	-0.493796038	0.271718665	0.069557609	0.164022393
Haemophilus parainfluenzae	-0.476148772	0.291260908	0.102505499	0.212544208
Alcaligenes faecalis	-0.282412184	0.13459977	0.036231755	0.109648732

Species	coef	stderr	pval	qval
Alcaligenes aquatilis	-0.389357455	0.223783538	0.082299377	0.185577026
Comamonas kerstersii	-0.54642935	0.230891323	0.018287881	0.065044526
Sutterella megalosphaeroides	-0.500139577	0.237774077	0.035750526	0.109149837
Sutterella faecalis	-0.49148589	0.223696436	0.028307814	0.089598127
Brevundimonas diminuta	-0.711412892	0.228605719	0.002004479	0.013830907
Brevundimonas naejangsanensis	-0.903356863	0.221590388	5.32E-05	0.001667838
Desulfovibrio piger	-0.411982017	0.223965829	0.066248064	0.160862687
Campylobacter jejuni	0.333362376	0.134187071	0.013201599	0.050534712
Lachnospiraceae bacterium Choco86	0.341891291	0.10134147	0.00079683	0.007636286
Lachnospiraceae bacterium	0.602313144	0.113934094	1.62E-07	1.87E-05
Lachnospiraceae bacterium KGMB03038	0.281815826	0.09316887	0.002666049	0.017002908
[Ruminococcus] gnavus	1.046117423	0.161268501	1.59E-10	5.49E-08
Blautia sp. SC05B48	0.286518316	0.132589474	0.031054271	0.095658244
Blautia argi	0.612331278	0.115239146	1.41E-07	1.87E-05
Blautia producta	0.34485434	0.105060713	0.001075474	0.009764173
Anaerostipes hadrus	0.362691893	0.155860684	0.02023574	0.07057853
Anaerostipes rhamnosivorans	0.322361473	0.098849218	0.001201313	0.009867925
Anaerobutyricum hallii	0.284968561	0.138164597	0.039501311	0.115491121
[Clostridium] hylemonae	0.315391694	0.095811718	0.001040746	0.009704254
[Clostridium] scindens	0.369396605	0.100025398	0.000237197	0.003273316
Lachnoclostridium sp. YL32	0.330382019	0.112254037	0.00343592	0.018815754
Lachnoclostridium phocaeense	0.262655897	0.089910287	0.003587377	0.019136823
Lachnoclostridium phytofermentans	0.229002937	0.087127836	0.008758137	0.039241003
Enterocloster bolteae	0.434155332	0.144421363	0.002739945	0.017002908
Enterocloster clostridioformis	0.295706205	0.121775421	0.015397156	0.055915987
Lachnospira eligens	-0.408173666	0.158651336	0.010275331	0.043231575
Butyrivibrio hungatei	0.143391263	0.092429167	0.121257042	0.241813176
Lacrimispora sphenoides	0.33209412	0.097199206	0.000667136	0.006769472
Lacrimispora saccharolytica	0.255213962	0.099732991	0.01068772	0.043840648
Anaerocolumna sedimenticola	0.232754869	0.083887245	0.005718026	0.027784775
Lachnoanaerobaculum umeaense	0.216617838	0.085360709	0.01147779	0.046044622
Anaerotignum propionicum	0.272640452	0.090941076	0.002809176	0.017002908
Herbinix luporum	0.149575583	0.095147504	0.116354408	0.235574141
Faecalibacterium prausnitzii	-0.569587704	0.180621773	0.001676815	0.012052111
Ruminococcus sp. JE7A12	-0.400231655	0.19939131	0.045074731	0.122447103
Ruminococcus bicirculans	-0.581537262	0.229929611	0.011629662	0.046117626
Ruminococcus albus	-0.365033047	0.119848548	0.002399412	0.015919175
Ruminococcus champanellensis	-0.445579275	0.158060633	0.00493961	0.025435305
Dysosmobacter welbionis	-0.222673367	0.133419889	0.095528081	0.203439432
Oscillibacter sp. PEA192	-0.535735193	0.168779493	0.001562485	0.011469306
Oscillibacter valericigenes	-0.586958563	0.160081811	0.000262572	0.003484126
Caproiciproducens sp. NJN-50	-0.212718243	0.106772661	0.046694494	0.125856253
Ethanoligenens harbinense	-0.464634462	0.122188063	0.000154496	0.002622839
Clostridioides difficile	0.318104465	0.080972163	9.31E-05	0.001707394
Peptacetobacter hiranonis	0.244846402	0.120486465	0.042503229	0.119860754
Massilistercora timonensis	0.282649454	0.129619339	0.029522649	0.091759586
Flintibacter sp. KGMB00164	-0.247641389	0.127756411	0.052939503	0.13528984
Mogibacterium diversum	0.363873905	0.122879322	0.003162543	0.017886516
[Eubacterium] sulci	0.378457343	0.105676622	0.000363241	0.004321317
Intestinimonas butyriciproducens	-0.517256851	0.165559899	0.001848766	0.01301682

Species	coef	stderr	pval	qval
Clostridium sp. DL-VIII	0.222388511	0.095842221	0.020717526	0.071475466
Clostridium perfringens	0.252771785	0.12788907	0.048485759	0.128673744
Clostridium botulinum	0.160092389	0.096101307	0.096195727	0.203604452
Clostridium butyricum	0.177955132	0.115562443	0.124143041	0.244739138
Clostridium pasteurianum	0.156652764	0.078962245	0.047852493	0.127977599
Clostridium saccharoperbutylacetonicum	0.177948553	0.093031889	0.056204409	0.141536652
Clostridium beijerinckii	0.273808377	0.097559869	0.005163146	0.025815731
Clostridium bornimense	0.190571818	0.103595	0.066380803	0.160862687
Clostridium baratii	0.235970173	0.13200492	0.074465522	0.170507617
Clostridium chauvoei	0.242711744	0.153577289	0.11471897	0.235392901
Clostridium novyi	0.163933949	0.099212324	0.098879545	0.20674814
Hungatella hathewayi	0.308935323	0.104407628	0.003227528	0.017959631
Eubacterium limosum	0.36368045	0.122223791	0.003047643	0.017820964
Eubacterium callanderi	0.385871564	0.125305422	0.002147137	0.01452475
Enterococcus faecium	0.436808382	0.190067919	0.021817549	0.074476479
Streptococcus salivarius	0.776916722	0.221193401	0.000474617	0.005282026
Streptococcus vestibularis	0.843530883	0.205114896	4.60E-05	0.00158678
Streptococcus sp. LPB0220	0.863611389	0.214505626	6.77E-05	0.001681013
Streptococcus sp. HSISM1	0.829455032	0.210250684	9.40E-05	0.001707394
Streptococcus sp. A12	0.315558096	0.171830236	0.066676418	0.160862687
Streptococcus sp. oral taxon 431	0.467614809	0.15678811	0.002965796	0.017641375
Streptococcus sp. FDAARGOS_192	0.739073996	0.225675302	0.0011089	0.009777178
Streptococcus pneumoniae	0.45692106	0.109647039	3.45E-05	0.001321633
Streptococcus dysgalactiae	0.204450569	0.104717545	0.051291491	0.134057307
Streptococcus pasteurianus	0.623023639	0.164203473	0.000159651	0.002622839
Streptococcus thermophilus	0.299764793	0.183569475	0.102883718	0.212544208
Streptococcus mitis	0.737487798	0.148345126	8.24E-07	5.69E-05
Streptococcus sanguinis	0.305473326	0.134643812	0.023557658	0.078586491
Streptococcus ilei	0.361089307	0.188311981	0.055541008	0.14089447
Streptococcus parasanguinis	0.889715975	0.203328974	1.38E-05	0.000594535
Streptococcus oralis	0.391102484	0.144775652	0.007060608	0.032478797
Streptococcus cristatus	0.522704732	0.130537522	6.82E-05	0.001681013
Streptococcus equinus	0.398470981	0.163092132	0.014778781	0.054241272
Streptococcus australis	0.362542073	0.160209379	0.023917628	0.078586491
Streptococcus koreensis	0.341017021	0.190509314	0.073839681	0.170507617
Streptococcus gordonii	0.253452031	0.146492857	0.08400783	0.18698517
Streptococcus viridans	0.34004892	0.173017561	0.049724552	0.130953973
Streptococcus infantarius	0.328556817	0.194293319	0.091306931	0.198118812
Streptococcus gallolyticus	0.362737634	0.214269298	0.090930632	0.198118812
Lactobacillus johnsonii	0.301822423	0.114727807	0.008732734	0.039241003
Limosilactobacillus fermentum	-0.365770264	0.181030105	0.04367565	0.120810289
Limosilactobacillus mucosae	-0.468963602	0.272770618	0.085966902	0.190119111
Bacillus sp. FJAT-22090	-0.22059604	0.144526019	0.127334595	0.249604745
Bacillus sp. N3536	-0.550249756	0.243012203	0.023831903	0.078586491
Psychrobacillus glaciei	-0.427284461	0.156716737	0.006546072	0.030518847
Psychrobacillus sp. AK 1817	-0.588296435	0.263518134	0.025868836	0.083408863
Sporosarcina psychrophila	-0.477611245	0.189691658	0.012008843	0.047080124
Gemella sanguinis	0.544405014	0.149594216	0.000292942	0.003743153
[Clostridium] innocuum	0.407174226	0.140927093	0.004058018	0.021212367
Erysipelotrichaceae bacterium GAM147	0.313268107	0.167213278	0.061509677	0.153774194

Species	coef	stderr	pval	qval
Absiella argi	0.612370112	0.125494445	1.29E-06	7.42E-05
Amedibacterium intestinale	0.853199662	0.166473592	3.80E-07	3.27E-05
Acidaminococcus fermentans	-0.229037217	0.134136587	0.088131058	0.19366379
Megamonas funiformis	1.072354758	0.410291166	0.00913205	0.039997316
Megamonas hypermegale	0.73253533	0.197567709	0.000223996	0.003266395
Veillonella atypica	0.395962196	0.225563395	0.079741354	0.180991889
Dialister massiliensis	-0.659133068	0.323635891	0.042024533	0.119822015
Finegoldia magna	0.29520411	0.097351661	0.002513332	0.016360366
Bifidobacterium breve	0.336962954	0.16684895	0.043771844	0.120810289
Bifidobacterium dentium	-0.378020002	0.205285793	0.065940762	0.160862687
Actinomyces sp. oral taxon 414	0.288834583	0.148222909	0.051699683	0.134108199
Schaalia odontolytica	0.597308044	0.151853791	9.17E-05	0.001707394
Rhodococcus erythropolis	-0.567906253	0.236004273	0.016356217	0.058780154
Microbacterium oxydans	-0.370286147	0.236194457	0.117428387	0.235574141
Arthrobacter sp. YC-RL1	-0.429749946	0.196081564	0.028698783	0.090009818
Rothia mucilaginosa	0.291709239	0.162975927	0.073864738	0.170507617
Oerskovia sp. KBS0722	-0.39367424	0.188471222	0.037079821	0.110280503
Gordonibacter urolithinfaciens	-0.354078907	0.174446272	0.042732964	0.119860754
Gordonibacter pamelaeae	-0.472941401	0.190438185	0.013237092	0.050534712
Arabia massiliensis	-0.558449457	0.170912285	0.001133586	0.009777178
Adlercreutzia sp. 8CFCBH1	-0.765818034	0.293574241	0.00927474	0.039997316
Adlercreutzia equolifaciens	-0.870614367	0.354274053	0.014238108	0.053392904
Olsenella sp. GAM18	-0.318796748	0.164296133	0.052696029	0.13528984
Akkermansia muciniphila	-0.540233506	0.260826698	0.038667325	0.114019035
Fusobacterium varium	0.921232467	0.205469833	8.46E-06	0.000416828
Fusobacterium ulcerans	0.592172845	0.2142223	0.005844052	0.028002751
Fusobacterium nucleatum	0.257131057	0.103657968	0.013329446	0.050534712
Fusobacterium mortiferum	0.953339163	0.281722842	0.000750657	0.007399335

Supplementary Table 23 Sensitivity analysis (exclusion of who received anti-hyperlipidemia therapy)
Mediating effect of gut microbiota between HLBS and dyslipidemia

Species	ACME	ADE	Proportion	P value of proportion
Bacteroides intestinalis	-0.002 (-0.005 to 0)	-0.085 (-0.099 to -0.066)	0.016 (-0.004 to 0.055)	0.15
Aeromonas veronii	-0.005 (-0.01 to -0.001)	-0.082 (-0.098 to -0.061)	0.05 (0.013 to 0.118)	0.002
Escherichia coli	-0.002 (-0.005 to 0)	-0.085 (-0.099 to -0.066)	0.02 (-0.006 to 0.062)	0.12
Escherichia albertii	-0.002 (-0.005 to 0)	-0.085 (-0.099 to -0.063)	0.021 (-0.004 to 0.061)	0.11
Escherichia marmotae	-0.003 (-0.006 to 0)	-0.084 (-0.098 to -0.064)	0.027 (0.003 to 0.071)	0.028
Psychrobacter sp. P11G5	-0.001 (-0.003 to 0.001)	-0.086 (-0.101 to -0.067)	0.006 (-0.017 to 0.038)	0.54
Haemophilus parainfluenzae	-0.002 (-0.005 to 0)	-0.084 (-0.1 to -0.066)	0.023 (-0.004 to 0.062)	0.106
Brevundimonas naejangsanensis	-0.002 (-0.004 to 0)	-0.085 (-0.1 to -0.064)	0.017 (-0.005 to 0.05)	0.108
Lachnospiraceae bacterium Choco86	0 (-0.003 to 0.002)	-0.086 (-0.1 to -0.065)	0.005 (-0.022 to 0.032)	0.688
Lachnospiraceae bacterium	-0.004 (-0.008 to -0.001)	-0.082 (-0.096 to -0.062)	0.045 (0.013 to 0.101)	0.002
Lachnospiraceae bacterium KGMB03038	0 (-0.003 to 0.002)	-0.086 (-0.1 to -0.068)	0.003 (-0.021 to 0.033)	0.74
[Ruminococcus] gnavus	-0.007 (-0.012 to -0.003)	-0.079 (-0.095 to -0.057)	0.08 (0.032 to 0.15)	0.002
Blautia argi	-0.002 (-0.006 to 0)	-0.084 (-0.098 to -0.062)	0.026 (-0.003 to 0.069)	0.09
Anaerostipes rhamnosivorans	0 (-0.002 to 0.002)	-0.087 (-0.1 to -0.065)	0.001 (-0.029 to 0.027)	0.962
[Clostridium] hylemonae	-0.001 (-0.003 to 0.001)	-0.085 (-0.101 to -0.065)	0.009 (-0.013 to 0.039)	0.386
[Clostridium] scindens	-0.001 (-0.004 to 0.002)	-0.086 (-0.1 to -0.068)	0.008 (-0.018 to 0.04)	0.47
Lachnoclostridium phocaeense	0 (-0.002 to 0.002)	-0.087 (-0.1 to -0.066)	0.001 (-0.025 to 0.027)	0.898
Enterocloster bolteae	0.001 (-0.001 to 0.003)	-0.087 (-0.102 to -0.068)	-0.006 (-0.04 to 0.016)	0.532
Lachnospira eligens	-0.002 (-0.005 to 0)	-0.085 (-0.099 to -0.065)	0.019 (-0.002 to 0.059)	0.09
Lacrimispora sphenoides	0 (-0.002 to 0.002)	-0.086 (-0.1 to -0.067)	0.003 (-0.022 to 0.031)	0.782
Anaerotignum propionicum	0 (-0.002 to 0.002)	-0.087 (-0.101 to -0.066)	-0.002 (-0.03 to 0.023)	0.884
Ruminococcus bicirculans	-0.002 (-0.006 to 0)	-0.085 (-0.1 to -0.064)	0.025 (0.001 to 0.065)	0.036
Ruminococcus albus	-0.003 (-0.007 to 0)	-0.084 (-0.098 to -0.065)	0.032 (0.001 to 0.081)	0.04
Clostridioides difficile	0 (-0.003 to 0.002)	-0.086 (-0.101 to -0.067)	0.002 (-0.025 to 0.033)	0.818
Hungatella hathewayi	0.001 (-0.002 to 0.003)	-0.087 (-0.102 to -0.067)	-0.007 (-0.038 to 0.019)	0.544
Eubacterium limosum	0 (-0.003 to 0.002)	-0.086 (-0.1 to -0.064)	0.003 (-0.025 to 0.035)	0.772
Streptococcus vestibularis	-0.002 (-0.005 to 0)	-0.085 (-0.099 to -0.067)	0.018 (-0.005 to 0.055)	0.126
Streptococcus sp. LPB0220	-0.002 (-0.006 to 0)	-0.085 (-0.1 to -0.064)	0.025 (0.003 to 0.067)	0.016
Streptococcus sp. HSISM1	-0.002 (-0.005 to 0)	-0.085 (-0.1 to -0.065)	0.021 (0 to 0.061)	0.05
Streptococcus pneumoniae	-0.002 (-0.006 to 0)	-0.084 (-0.099 to -0.063)	0.027 (0.002 to 0.073)	0.026
Streptococcus pasteurianus	-0.001 (-0.003 to 0.001)	-0.086 (-0.101 to -0.067)	0.007 (-0.008 to 0.036)	0.324
Streptococcus mitis	-0.004 (-0.007 to -0.001)	-0.083 (-0.098 to -0.063)	0.04 (0.01 to 0.089)	0.008
Streptococcus parasanguinis	-0.003 (-0.006 to 0)	-0.084 (-0.098 to -0.062)	0.028 (0.002 to 0.075)	0.034
Ligilactobacillus ruminis	0 (-0.001 to 0.001)	-0.086 (-0.101 to -0.066)	0 (-0.014 to 0.008)	0.832
Sporosarcina psychrophila	-0.001 (-0.003 to 0.002)	-0.086 (-0.101 to -0.065)	0.005 (-0.02 to 0.036)	0.554
Absiella argi	-0.001 (-0.004 to 0)	-0.085 (-0.1 to -0.064)	0.014 (-0.004 to 0.049)	0.134
Faecalitalea cylindroides	0 (-0.001 to 0.002)	-0.086 (-0.1 to -0.067)	-0.002 (-0.023 to 0.012)	0.688
Amedibacterium intestinale	-0.003 (-0.007 to -0.001)	-0.083 (-0.099 to -0.06)	0.036 (0.008 to 0.091)	0.008
Megamonas funiformis	-0.002 (-0.006 to 0)	-0.084 (-0.099 to -0.063)	0.025 (-0.001 to 0.065)	0.062
Megamonas hypermegale	-0.003 (-0.006 to 0)	-0.084 (-0.099 to -0.063)	0.028 (0.004 to 0.072)	0.022
Finegoldia magna	-0.002 (-0.005 to 0)	-0.085 (-0.099 to -0.065)	0.017 (-0.004 to 0.054)	0.128
Bifidobacterium breve	0 (-0.002 to 0.001)	-0.086 (-0.101 to -0.065)	0.001 (-0.014 to 0.021)	0.752
Bifidobacterium catenulatum	0 (-0.001 to 0.002)	-0.087 (-0.102 to -0.067)	0 (-0.019 to 0.014)	0.91
Fusobacterium varium	-0.003 (-0.006 to 0)	-0.084 (-0.099 to -0.063)	0.03 (0.005 to 0.078)	0.016
Fusobacterium nucleatum	-0.002 (-0.005 to 0.001)	-0.085 (-0.099 to -0.065)	0.016 (-0.006 to 0.057)	0.15

Supplementary Table 24 Sensitivity analysis (exclusion of who received anti-hyperlipidemia therapy)
Mediating effect of gut microbiota between HLBS and decreased HDL

Species	ACME	ADE	Proportion	P value of proportion
Bacteroides uniformis	-0.001 (-0.004 to 0.002)	-0.058 (-0.1 to -0.02)	0.012 (-0.031 to 0.086)	0.494
Bacteroides intestinalis	-0.001 (-0.004 to 0.001)	-0.058 (-0.1 to -0.022)	0.008 (-0.025 to 0.074)	0.562
Phocaeicola vulgatus	0 (-0.001 to 0.002)	-0.058 (-0.1 to -0.02)	-0.002 (-0.053 to 0.024)	0.734
Prevotella ruminicola	0 (-0.002 to 0.001)	-0.058 (-0.1 to -0.021)	0 (-0.025 to 0.036)	0.896
Prevotella melaninogenica	0 (-0.002 to 0.001)	-0.058 (-0.1 to -0.02)	0 (-0.025 to 0.032)	0.902
Prevotella fusca	0 (-0.002 to 0.001)	-0.058 (-0.099 to -0.021)	0.001 (-0.024 to 0.038)	0.822
Prevotella scopos	0 (-0.001 to 0.001)	-0.058 (-0.099 to -0.02)	0 (-0.021 to 0.028)	0.972
Enterobacter cloacae	0 (-0.002 to 0.001)	-0.058 (-0.1 to -0.021)	0.002 (-0.021 to 0.047)	0.74
Enterobacter kobei	-0.001 (-0.003 to 0.001)	-0.058 (-0.101 to -0.022)	0.008 (-0.029 to 0.063)	0.558
Enterobacter roggenkampii	-0.002 (-0.005 to 0.001)	-0.055 (-0.098 to -0.018)	0.024 (-0.01 to 0.113)	0.228
Klebsiella pneumoniae	0 (-0.002 to 0.002)	-0.059 (-0.102 to -0.02)	0.001 (-0.03 to 0.036)	0.916
Klebsiella oxytoca	-0.001 (-0.003 to 0.001)	-0.057 (-0.102 to -0.02)	0.006 (-0.024 to 0.065)	0.552
Klebsiella quasipneumoniae	-0.001 (-0.003 to 0.001)	-0.058 (-0.102 to -0.019)	0.007 (-0.02 to 0.062)	0.54
Klebsiella aerogenes	0 (-0.001 to 0.003)	-0.058 (-0.099 to -0.019)	-0.002 (-0.052 to 0.025)	0.748
Klebsiella sp. FDAARGOS_511	-0.001 (-0.005 to 0.001)	-0.055 (-0.099 to -0.019)	0.02 (-0.01 to 0.093)	0.188
Klebsiella variicola	0 (-0.002 to 0.002)	-0.057 (-0.099 to -0.021)	0.001 (-0.044 to 0.045)	0.92
Raoultella ornithinolytica	-0.001 (-0.005 to 0.002)	-0.056 (-0.099 to -0.019)	0.018 (-0.036 to 0.102)	0.428
Raoultella planticola	-0.003 (-0.007 to 0)	-0.053 (-0.096 to -0.016)	0.047 (-0.005 to 0.179)	0.088
Yersinia enterocolitica	0 (-0.001 to 0.002)	-0.058 (-0.102 to -0.021)	-0.001 (-0.039 to 0.022)	0.86
Acinetobacter johnsonii	0.001 (-0.001 to 0.003)	-0.06 (-0.104 to -0.02)	-0.008 (-0.08 to 0.014)	0.396
Psychrobacter sp. P11G5	-0.001 (-0.005 to 0.002)	-0.058 (-0.103 to -0.02)	0.016 (-0.037 to 0.087)	0.414
Haemophilus parainfluenzae	-0.002 (-0.006 to 0)	-0.055 (-0.099 to -0.02)	0.033 (-0.011 to 0.132)	0.128
Lachnospiraceae bacterium Choco86	0 (-0.002 to 0.002)	-0.058 (-0.101 to -0.022)	0 (-0.055 to 0.049)	0.96
Lachnospiraceae bacterium KM106-2	0 (-0.002 to 0.003)	-0.057 (-0.099 to -0.019)	-0.004 (-0.064 to 0.039)	0.718
Lachnospira eligens	-0.002 (-0.006 to 0)	-0.058 (-0.101 to -0.021)	0.029 (-0.006 to 0.108)	0.124
Dysosmobacter welbionis	0.002 (-0.002 to 0.007)	-0.059 (-0.101 to -0.023)	-0.033 (-0.176 to 0.037)	0.29
Oscillibacter sp. PEA192	0.001 (-0.002 to 0.006)	-0.059 (-0.102 to -0.023)	-0.025 (-0.124 to 0.041)	0.406
Oscillibacter valericigenes	0.001 (-0.003 to 0.006)	-0.059 (-0.105 to -0.022)	-0.018 (-0.13 to 0.053)	0.612
Caproiciproducens sp. NJN-50	0.002 (-0.002 to 0.006)	-0.058 (-0.1 to -0.019)	-0.024 (-0.145 to 0.035)	0.35
Ethanoligenens harbinense	0.002 (-0.002 to 0.006)	-0.059 (-0.104 to -0.022)	-0.029 (-0.161 to 0.032)	0.344
Flintibacter sp. KGMB00164	0.002 (-0.002 to 0.007)	-0.058 (-0.103 to -0.021)	-0.03 (-0.162 to 0.042)	0.358
Intestinimonas butyriciproducens	0.001 (-0.003 to 0.005)	-0.058 (-0.099 to -0.021)	-0.011 (-0.099 to 0.069)	0.706
Clostridium sp. SY8519	0.001 (-0.002 to 0.003)	-0.058 (-0.101 to -0.019)	-0.008 (-0.094 to 0.033)	0.552
Clostridiales bacterium CCNA10	0.002 (0 to 0.007)	-0.057 (-0.1 to -0.02)	-0.041 (-0.196 to 0.008)	0.106
Christensenella sp. Marseille-P3954	0.002 (-0.001 to 0.006)	-0.06 (-0.102 to -0.022)	-0.034 (-0.15 to 0.018)	0.202
Enterococcus avium	-0.001 (-0.004 to 0.002)	-0.058 (-0.098 to -0.018)	0.008 (-0.04 to 0.081)	0.584
Enterococcus casseliflavus	0 (-0.003 to 0.002)	-0.058 (-0.099 to -0.02)	0.004 (-0.042 to 0.06)	0.75
Streptococcus ilei	0 (-0.002 to 0.001)	-0.058 (-0.102 to -0.02)	0 (-0.029 to 0.035)	0.946
Streptococcus koreensis	0 (-0.002 to 0.002)	-0.057 (-0.101 to -0.021)	0 (-0.038 to 0.043)	0.904
Streptococcus viridans	0 (-0.002 to 0.001)	-0.058 (-0.101 to -0.021)	0.001 (-0.027 to 0.041)	0.882
Lactococcus lactis	0 (-0.002 to 0.002)	-0.059 (-0.101 to -0.021)	-0.002 (-0.045 to 0.039)	0.796
Lactococcus cremoris	-0.001 (-0.004 to 0.002)	-0.058 (-0.104 to -0.021)	0.009 (-0.034 to 0.073)	0.612
Ligilactobacillus ruminis	0 (-0.002 to 0.001)	-0.058 (-0.1 to -0.021)	0.001 (-0.024 to 0.036)	0.896
Leuconostoc mesenteroides	0.001 (-0.002 to 0.004)	-0.061 (-0.102 to -0.022)	-0.009 (-0.087 to 0.035)	0.59
Fructilactobacillus sanfranciscensis	0 (-0.001 to 0.002)	-0.058 (-0.099 to -0.023)	0 (-0.036 to 0.021)	0.914
Faecalitalea cylindroides	0 (-0.001 to 0.002)	-0.058 (-0.099 to -0.021)	-0.004 (-0.057 to 0.022)	0.646
Intestinibaculum porci	0 (-0.001 to 0.001)	-0.058 (-0.1 to -0.021)	0 (-0.026 to 0.027)	0.932
Megamonas funiformis	-0.004 (-0.01 to 0)	-0.053 (-0.093 to -0.017)	0.063 (0.005 to 0.204)	0.036

Megamonas hypermegale	-0.004 (-0.009 to 0)	-0.053 (-0.096 to -0.018)	0.072 (0.007 to 0.204)	0.018
Dialister massiliensis	-0.001 (-0.004 to 0.001)	-0.058 (-0.101 to -0.02)	0.011 (-0.024 to 0.095)	0.418
Dialister hominis	-0.001 (-0.005 to 0.001)	-0.057 (-0.097 to -0.019)	0.019 (-0.014 to 0.097)	0.258
Bifidobacterium longum	0.001 (-0.001 to 0.004)	-0.059 (-0.101 to -0.02)	-0.015 (-0.101 to 0.022)	0.33
Bifidobacterium adolescentis	0 (-0.001 to 0.002)	-0.06 (-0.105 to -0.022)	-0.002 (-0.047 to 0.024)	0.744
Bifidobacterium breve	0 (-0.002 to 0.001)	-0.058 (-0.101 to -0.02)	0 (-0.031 to 0.033)	0.904
Bifidobacterium catenulatum	0 (-0.002 to 0.003)	-0.058 (-0.1 to -0.021)	-0.002 (-0.062 to 0.05)	0.866
Bifidobacterium bifidum	0 (-0.003 to 0.001)	-0.058 (-0.102 to -0.019)	0.004 (-0.023 to 0.054)	0.616
Bifidobacterium angulatum	0 (-0.001 to 0.002)	-0.059 (-0.104 to -0.019)	-0.003 (-0.06 to 0.025)	0.66
Bifidobacterium pseudocatenulatum	0.001 (-0.002 to 0.004)	-0.059 (-0.103 to -0.019)	-0.01 (-0.097 to 0.047)	0.628
Olsenella sp. GAM18	0 (-0.003 to 0.003)	-0.059 (-0.103 to -0.022)	0 (-0.063 to 0.059)	0.98
Cloacibacillus porcorum	0.003 (-0.001 to 0.008)	-0.061 (-0.101 to -0.019)	-0.056 (-0.272 to 0.014)	0.122

Supplementary Table 25 Sensitivity analysis (exclusion of who received anti-hyperlipidemia therapy)
Mediating effect of gut microbiota between HLBS and elevated LDL

Species	ACME	ADE	Proportion	P value of proportion
[Ruminococcus] gnavus	-0.004 (-0.01 to 0)	-0.063 (-0.115 to -0.017)	0.06 (0 to 0.205)	0.05

Supplementary Table 26 Sensitivity analysis (exclusion of who received anti-hyperlipidemia therapy) Mediating effect of gut microbiota between HLBS and elevated TC

Species	ACME	ADE	Proportion	P value of proportion
Escherichia coli	-0.003 (-0.009 to 0.001)	-0.104 (-0.153 to -0.05)	0.029 (-0.006 to 0.099)	0.102
Escherichia albertii	-0.003 (-0.008 to 0)	-0.107 (-0.156 to -0.055)	0.023 (-0.005 to 0.082)	0.096
Escherichia fergusonii	-0.002 (-0.008 to 0.001)	-0.108 (-0.158 to -0.057)	0.019 (-0.008 to 0.07)	0.206
Escherichia marmotae	-0.003 (-0.008 to 0)	-0.105 (-0.156 to -0.052)	0.028 (-0.001 to 0.092)	0.066
Citrobacter tructae	-0.001 (-0.005 to 0.003)	-0.109 (-0.158 to -0.056)	0.006 (-0.026 to 0.049)	0.654
[Ruminococcus] gnavus	-0.007 (-0.015 to -0.002)	-0.1 (-0.151 to -0.046)	0.064 (0.016 to 0.154)	0.004
Ruminococcus bicirculans	-0.004 (-0.011 to 0)	-0.105 (-0.155 to -0.055)	0.031 (-0.002 to 0.107)	0.066
Streptococcus pneumoniae	-0.005 (-0.011 to 0)	-0.105 (-0.153 to -0.052)	0.041 (0.003 to 0.108)	0.03
Streptococcus mitis	-0.005 (-0.012 to 0)	-0.107 (-0.153 to -0.055)	0.042 (0.004 to 0.108)	0.022
Streptococcus oralis	-0.002 (-0.007 to 0.001)	-0.108 (-0.156 to -0.053)	0.015 (-0.014 to 0.067)	0.262
Streptococcus cristatus	-0.004 (-0.009 to 0)	-0.109 (-0.155 to -0.054)	0.034 (0.003 to 0.092)	0.03
Megamonas funiformis	-0.003 (-0.009 to 0.001)	-0.106 (-0.154 to -0.052)	0.03 (-0.009 to 0.102)	0.14
Fusobacterium varium	-0.003 (-0.008 to 0)	-0.105 (-0.153 to -0.054)	0.03 (-0.002 to 0.087)	0.076
Fusobacterium mortiferum	-0.005 (-0.011 to -0.001)	-0.102 (-0.151 to -0.048)	0.044 (0.005 to 0.126)	0.03

Supplementary Table 27 Sensitivity analysis (exclusion of who received anti-hyperlipidemia therapy) Mediating effect of gut microbiota between HLBS and elevated TG

Species	ACME	ADE	Proportion	P value of proportion
Bacteroides uniformis	-0.001 (-0.005 to 0.001)	-0.127 (-0.147 to -0.099)	0.01 (-0.005 to 0.039)	0.206
Bacteroides cellulosilyticus	-0.002 (-0.005 to 0.001)	-0.126 (-0.146 to -0.097)	0.011 (-0.006 to 0.042)	0.2
Bacteroides intestinalis	-0.002 (-0.007 to 0.001)	-0.126 (-0.146 to -0.096)	0.014 (-0.007 to 0.051)	0.166
Bacteroides zoogleoformans	-0.001 (-0.003 to 0.001)	-0.127 (-0.148 to -0.097)	0.003 (-0.01 to 0.024)	0.572
Tannerella forsythia	0 (-0.004 to 0.003)	-0.128 (-0.147 to -0.1)	0.002 (-0.021 to 0.03)	0.804
Tannerella sp. oral taxon HOT-286	0 (-0.003 to 0.002)	-0.128 (-0.147 to -0.1)	0.002 (-0.013 to 0.022)	0.732
Paraprevotella xylaniphila	0 (-0.002 to 0.003)	-0.128 (-0.148 to -0.097)	-0.002 (-0.025 to 0.018)	0.77
Prevotella intermedia	0 (-0.003 to 0.003)	-0.129 (-0.15 to -0.099)	0.001 (-0.022 to 0.024)	0.92
Prevotella ruminicola	0 (-0.002 to 0.003)	-0.128 (-0.148 to -0.1)	-0.002 (-0.027 to 0.019)	0.808
Prevotella dentalis	0 (-0.004 to 0.004)	-0.128 (-0.147 to -0.098)	0.001 (-0.028 to 0.029)	0.918
Prevotella melaninogenica	0 (-0.002 to 0.003)	-0.129 (-0.148 to -0.099)	-0.001 (-0.026 to 0.016)	0.798
Prevotella fusca	0.001 (-0.002 to 0.004)	-0.129 (-0.148 to -0.1)	-0.004 (-0.033 to 0.013)	0.514
Prevotella denticola	0 (-0.003 to 0.004)	-0.128 (-0.148 to -0.097)	-0.001 (-0.029 to 0.025)	0.89
Prevotella oris	0 (-0.003 to 0.003)	-0.128 (-0.148 to -0.096)	0.001 (-0.024 to 0.025)	0.896
Prevotella jejuni	0 (-0.002 to 0.003)	-0.129 (-0.149 to -0.101)	-0.002 (-0.028 to 0.017)	0.73
Prevotella sp. oral taxon 299	0 (-0.002 to 0.003)	-0.13 (-0.149 to -0.104)	-0.003 (-0.028 to 0.019)	0.732
Prevotella enoeca	0 (-0.004 to 0.004)	-0.129 (-0.148 to -0.102)	-0.001 (-0.03 to 0.028)	0.934
Pseudoprevotella muciniphila	0 (-0.002 to 0.003)	-0.129 (-0.149 to -0.098)	0 (-0.024 to 0.018)	0.918
Butyricimonas faecalis	-0.003 (-0.007 to 0)	-0.126 (-0.146 to -0.098)	0.021 (0.001 to 0.063)	0.044
Odoribacter splanchnicus	-0.003 (-0.007 to 0)	-0.125 (-0.145 to -0.096)	0.021 (-0.004 to 0.058)	0.112
Alistipes shahii	-0.004 (-0.01 to -0.001)	-0.123 (-0.144 to -0.092)	0.032 (0.004 to 0.079)	0.022
Alistipes megaguti	-0.001 (-0.004 to 0.002)	-0.127 (-0.147 to -0.097)	0.004 (-0.017 to 0.032)	0.632
Alistipes communis	-0.005 (-0.01 to -0.001)	-0.123 (-0.143 to -0.095)	0.037 (0.006 to 0.084)	0.012
Alistipes sp. dk3624	-0.003 (-0.007 to 0)	-0.126 (-0.145 to -0.098)	0.019 (-0.002 to 0.056)	0.086
Alistipes finegoldii	-0.003 (-0.008 to 0)	-0.126 (-0.145 to -0.097)	0.023 (0.002 to 0.065)	0.03
Alistipes onderdonkii	-0.003 (-0.009 to 0)	-0.125 (-0.145 to -0.096)	0.024 (0.001 to 0.069)	0.04
Alistipes dispar	-0.002 (-0.006 to 0)	-0.126 (-0.146 to -0.095)	0.015 (-0.002 to 0.047)	0.09
Muribaculum gordoncarteri	-0.003 (-0.007 to 0)	-0.126 (-0.147 to -0.098)	0.019 (-0.004 to 0.055)	0.126
Muribaculum intestinale	-0.001 (-0.005 to 0.002)	-0.128 (-0.147 to -0.1)	0.008 (-0.015 to 0.041)	0.48
Duncaniella dubosii	-0.001 (-0.004 to 0.002)	-0.127 (-0.147 to -0.098)	0.006 (-0.015 to 0.033)	0.58
Petrimonas mucosa	0.001 (-0.002 to 0.004)	-0.129 (-0.149 to -0.101)	-0.003 (-0.03 to 0.016)	0.692
Proteiniphilum saccharofermentans	0 (-0.002 to 0.003)	-0.129 (-0.148 to -0.099)	-0.002 (-0.026 to 0.015)	0.692
Barnesiella viscericola	0 (-0.004 to 0.003)	-0.128 (-0.148 to -0.1)	0.004 (-0.024 to 0.035)	0.752
Porphyromonas gingivalis	0 (-0.003 to 0.004)	-0.13 (-0.149 to -0.099)	-0.002 (-0.032 to 0.025)	0.852
Porphyromonas asaccharolytica	-0.002 (-0.006 to 0.002)	-0.127 (-0.148 to -0.101)	0.012 (-0.017 to 0.05)	0.352
Pseudomonas versuta	0 (-0.003 to 0.002)	-0.127 (-0.146 to -0.098)	0.001 (-0.016 to 0.024)	0.812
Pseudomonas taetrolens	0.001 (-0.002 to 0.006)	-0.128 (-0.148 to -0.099)	-0.007 (-0.047 to 0.015)	0.476
Pseudomonas stutzeri	-0.001 (-0.003 to 0.001)	-0.127 (-0.147 to -0.096)	0.004 (-0.009 to 0.027)	0.508
Aeromonas veronii	-0.008 (-0.017 to -0.002)	-0.119 (-0.139 to -0.087)	0.063 (0.018 to 0.14)	0
Escherichia coli	-0.003 (-0.008 to 0.001)	-0.124 (-0.144 to -0.09)	0.023 (-0.005 to 0.065)	0.12
Escherichia albertii	-0.002 (-0.006 to 0)	-0.125 (-0.146 to -0.096)	0.018 (-0.002 to 0.05)	0.072
Escherichia fergusonii	-0.002 (-0.005 to 0.001)	-0.126 (-0.147 to -0.098)	0.011 (-0.012 to 0.046)	0.296
Escherichia marmotae	-0.004 (-0.009 to 0)	-0.124 (-0.143 to -0.091)	0.027 (0.003 to 0.072)	0.028
Salmonella enterica	0 (-0.002 to 0.002)	-0.128 (-0.149 to -0.098)	-0.001 (-0.018 to 0.013)	0.838
Lelliottia sp. WB101	-0.001 (-0.004 to 0.002)	-0.127 (-0.147 to -0.098)	0.005 (-0.015 to 0.036)	0.554
Psychrobacter sp. P11G5	0 (-0.003 to 0.003)	-0.128 (-0.148 to -0.099)	0.001 (-0.026 to 0.027)	0.86
Haemophilus parainfluenzae	-0.001 (-0.005 to 0.001)	-0.127 (-0.146 to -0.098)	0.01 (-0.01 to 0.042)	0.278
Alcaligenes faecalis	-0.001 (-0.005 to 0.001)	-0.126 (-0.147 to -0.097)	0.009 (-0.005 to 0.038)	0.22
Alcaligenes aquatilis	-0.001 (-0.003 to 0.001)	-0.127 (-0.146 to -0.095)	0.003 (-0.009 to 0.027)	0.596
Comamonas kerstersii	-0.003 (-0.008 to 0.001)	-0.124 (-0.145 to -0.093)	0.022 (-0.004 to 0.065)	0.088
Sutterella megalosphaeroides	-0.001 (-0.005 to 0.001)	-0.126 (-0.146 to -0.099)	0.009 (-0.005 to 0.037)	0.268
Sutterella faecalis	-0.001 (-0.004 to 0.001)	-0.127 (-0.147 to -0.099)	0.007 (-0.006 to 0.036)	0.304
Brevundimonas diminuta	-0.001 (-0.005 to 0.001)	-0.127 (-0.146 to -0.099)	0.009 (-0.01 to 0.039)	0.328
Brevundimonas naejangsanensis	-0.003 (-0.008 to 0)	-0.123 (-0.144 to -0.095)	0.025 (-0.001 to 0.066)	0.056

Species	ACME	ADE	Proportion	P value of proportion
Desulfovibrio piger	-0.001 (-0.004 to 0.001)	-0.127 (-0.146 to -0.097)	0.007 (-0.008 to 0.035)	0.356
Campylobacter jejuni	-0.001 (-0.004 to 0.002)	-0.126 (-0.146 to -0.097)	0.004 (-0.017 to 0.032)	0.578
Lachnospiraceae bacterium Choco86	-0.001 (-0.005 to 0.004)	-0.125 (-0.146 to -0.094)	0.006 (-0.032 to 0.041)	0.684
Lachnospiraceae bacterium	-0.006 (-0.012 to -0.001)	-0.12 (-0.141 to -0.091)	0.045 (0.008 to 0.102)	0.016
Lachnospiraceae bacterium KGMB03038	-0.001 (-0.004 to 0.003)	-0.126 (-0.147 to -0.098)	0.004 (-0.025 to 0.035)	0.736
[Ruminococcus] gnavus	-0.009 (-0.016 to -0.003)	-0.118 (-0.139 to -0.088)	0.068 (0.025 to 0.131)	0.004
Blautia sp. SC05B48	0 (-0.003 to 0.002)	-0.127 (-0.148 to -0.1)	0.001 (-0.018 to 0.023)	0.82
Blautia argi	-0.004 (-0.01 to 0.001)	-0.123 (-0.143 to -0.092)	0.033 (-0.005 to 0.08)	0.092
Blautia producta	0 (-0.004 to 0.003)	-0.127 (-0.147 to -0.095)	0.002 (-0.027 to 0.031)	0.844
Anaerostipes hadrus	0.003 (0 to 0.008)	-0.13 (-0.151 to -0.101)	-0.026 (-0.068 to -0.003)	0.03
Anaerostipes rhamnosivorans	-0.001 (-0.004 to 0.003)	-0.127 (-0.147 to -0.097)	0.004 (-0.022 to 0.033)	0.73
Anaerobutyricum hallii	0 (-0.002 to 0.003)	-0.127 (-0.148 to -0.099)	-0.002 (-0.026 to 0.015)	0.73
[Clostridium] hylemonae	-0.002 (-0.006 to 0.002)	-0.125 (-0.145 to -0.097)	0.01 (-0.014 to 0.047)	0.354
[Clostridium] scindens	-0.002 (-0.006 to 0.002)	-0.125 (-0.146 to -0.093)	0.011 (-0.017 to 0.048)	0.442
Lachnoclostridium sp. YL32	0 (-0.004 to 0.004)	-0.128 (-0.148 to -0.099)	-0.001 (-0.034 to 0.028)	0.898
Lachnoclostridium phocaeense	0 (-0.004 to 0.003)	-0.126 (-0.146 to -0.095)	0.002 (-0.027 to 0.029)	0.842
Lachnoclostridium phytofermentans	0 (-0.003 to 0.003)	-0.127 (-0.147 to -0.097)	0 (-0.022 to 0.023)	0.98
Enterocloster bolteae	0 (-0.003 to 0.004)	-0.127 (-0.147 to -0.097)	-0.001 (-0.031 to 0.026)	0.968
Enterocloster clostridioformis	0.001 (-0.001 to 0.005)	-0.128 (-0.148 to -0.098)	-0.009 (-0.042 to 0.009)	0.298
Lachnospira eligens	-0.004 (-0.008 to 0)	-0.125 (-0.145 to -0.096)	0.025 (0.001 to 0.068)	0.05
Butyrivibrio hungatei	0.001 (-0.001 to 0.004)	-0.128 (-0.147 to -0.097)	-0.004 (-0.031 to 0.01)	0.54
Lacrimispora sphenoides	-0.001 (-0.006 to 0.002)	-0.125 (-0.146 to -0.094)	0.008 (-0.018 to 0.042)	0.464
Lacrimispora saccharolytica	-0.001 (-0.004 to 0.002)	-0.126 (-0.147 to -0.097)	0.003 (-0.018 to 0.03)	0.686
Anaerocolumna sedimenticola	0 (-0.004 to 0.003)	-0.126 (-0.147 to -0.096)	0.002 (-0.026 to 0.029)	0.816
Lachnoanaerobaculum umeaense	0 (-0.003 to 0.003)	-0.127 (-0.147 to -0.096)	-0.001 (-0.029 to 0.023)	0.912
Anaerotignum propionicum	0 (-0.004 to 0.004)	-0.127 (-0.147 to -0.099)	-0.002 (-0.035 to 0.031)	0.904
Herbinix luporum	0.001 (-0.001 to 0.004)	-0.128 (-0.148 to -0.098)	-0.006 (-0.038 to 0.007)	0.342
Faecalibacterium prausnitzii	-0.004 (-0.009 to 0)	-0.125 (-0.144 to -0.097)	0.032 (0.003 to 0.076)	0.032
Ruminococcus sp. JE7A12	-0.001 (-0.004 to 0.001)	-0.126 (-0.146 to -0.095)	0.009 (-0.006 to 0.036)	0.272
Ruminococcus bicirculans	-0.003 (-0.007 to 0)	-0.125 (-0.145 to -0.095)	0.021 (-0.002 to 0.059)	0.078
Ruminococcus albus	-0.004 (-0.009 to 0)	-0.126 (-0.146 to -0.097)	0.027 (0 to 0.071)	0.052
Ruminococcus champanellensis	-0.003 (-0.009 to 0.001)	-0.125 (-0.144 to -0.094)	0.025 (-0.006 to 0.072)	0.118
Dysosmobacter welbionis	-0.001 (-0.005 to 0.003)	-0.126 (-0.146 to -0.098)	0.008 (-0.022 to 0.042)	0.554
Oscillibacter sp. PEA192	-0.004 (-0.009 to 0)	-0.124 (-0.146 to -0.094)	0.03 (0.003 to 0.075)	0.032
Oscillibacter valericigenes	-0.005 (-0.011 to -0.001)	-0.123 (-0.143 to -0.097)	0.036 (0.005 to 0.091)	0.02
Caproiciproducens sp. NJN-50	-0.002 (-0.006 to 0.002)	-0.126 (-0.147 to -0.095)	0.011 (-0.013 to 0.046)	0.324
Ethanoligenens harbinense	-0.005 (-0.011 to -0.001)	-0.123 (-0.143 to -0.094)	0.038 (0.009 to 0.09)	0.016
Clostridioides difficile	-0.001 (-0.005 to 0.003)	-0.126 (-0.146 to -0.097)	0.004 (-0.028 to 0.038)	0.766
Peptacetobacter hiranonis	-0.001 (-0.005 to 0.001)	-0.126 (-0.147 to -0.096)	0.008 (-0.006 to 0.038)	0.258
Massilistercora timonensis	0 (-0.003 to 0.002)	-0.127 (-0.147 to -0.099)	0.002 (-0.019 to 0.024)	0.806
Flintibacter sp. KGMB00164	-0.002 (-0.006 to 0.002)	-0.127 (-0.148 to -0.097)	0.012 (-0.016 to 0.046)	0.352
Mogibacterium diversum	-0.002 (-0.006 to 0)	-0.126 (-0.146 to -0.096)	0.015 (-0.004 to 0.046)	0.132
[Eubacterium] sulci	-0.001 (-0.005 to 0.002)	-0.128 (-0.148 to -0.098)	0.006 (-0.018 to 0.037)	0.588
Intestinimonas butyriciproducens	-0.004 (-0.008 to 0)	-0.125 (-0.145 to -0.095)	0.029 (-0.003 to 0.071)	0.078
Clostridium sp. DL-VIII	-0.002 (-0.006 to 0.001)	-0.127 (-0.147 to -0.096)	0.013 (-0.005 to 0.046)	0.188
Clostridium perfringens	-0.002 (-0.008 to 0.003)	-0.126 (-0.147 to -0.097)	0.015 (-0.023 to 0.065)	0.38
Clostridium botulinum	-0.001 (-0.004 to 0.002)	-0.127 (-0.147 to -0.098)	0.008 (-0.012 to 0.037)	0.364
Clostridium butyricum	-0.001 (-0.004 to 0.001)	-0.127 (-0.148 to -0.098)	0.006 (-0.012 to 0.031)	0.41
Clostridium pasteurianum	-0.001 (-0.005 to 0.001)	-0.127 (-0.147 to -0.098)	0.009 (-0.007 to 0.038)	0.268
Clostridium saccharoperbutylacetonicum	-0.002 (-0.005 to 0.002)	-0.127 (-0.147 to -0.097)	0.011 (-0.012 to 0.044)	0.366
Clostridium beijerinckii	-0.003 (-0.007 to 0)	-0.125 (-0.146 to -0.094)	0.019 (-0.004 to 0.056)	0.116
Clostridium bornimense	-0.001 (-0.006 to 0.002)	-0.127 (-0.147 to -0.097)	0.01 (-0.014 to 0.046)	0.426
Clostridium baratii	-0.001 (-0.005 to 0.002)	-0.126 (-0.147 to -0.098)	0.011 (-0.014 to 0.041)	0.368
Clostridium chauvoei	-0.001 (-0.005 to 0.002)	-0.127 (-0.147 to -0.097)	0.007 (-0.014 to 0.04)	0.46
Clostridium novyi	-0.001 (-0.004 to 0.001)	-0.127 (-0.146 to -0.096)	0.006 (-0.008 to 0.03)	0.414
Hungatella hathewayi	0 (-0.003 to 0.004)	-0.127 (-0.147 to -0.099)	0 (-0.03 to 0.027)	0.966
Eubacterium limosum	0 (-0.004 to 0.003)	-0.127 (-0.147 to -0.099)	0.002 (-0.024 to 0.029)	0.84

Species	ACME	ADE	Proportion	P value of proportion
Eubacterium callanderi	0.001 (-0.002 to 0.005)	-0.128 (-0.149 to -0.097)	-0.007 (-0.042 to 0.019)	0.522
Enterococcus faecium	-0.002 (-0.005 to 0.001)	-0.126 (-0.147 to -0.098)	0.011 (-0.006 to 0.041)	0.214
Streptococcus salivarius	-0.002 (-0.007 to 0.002)	-0.127 (-0.147 to -0.098)	0.018 (-0.012 to 0.057)	0.244
Streptococcus vestibularis	-0.003 (-0.008 to 0.001)	-0.126 (-0.145 to -0.098)	0.023 (-0.01 to 0.068)	0.142
Streptococcus sp. LPB0220	-0.006 (-0.011 to -0.001)	-0.123 (-0.144 to -0.095)	0.043 (0.01 to 0.095)	0.002
Streptococcus sp. HSISM1	-0.005 (-0.01 to 0)	-0.124 (-0.145 to -0.094)	0.033 (0 to 0.079)	0.058
Streptococcus sp. A12	-0.001 (-0.003 to 0.001)	-0.127 (-0.146 to -0.096)	0.003 (-0.011 to 0.027)	0.582
Streptococcus sp. oral taxon 431	-0.001 (-0.004 to 0.003)	-0.127 (-0.147 to -0.099)	0.004 (-0.021 to 0.032)	0.724
Streptococcus sp. FDAARGOS_192	-0.002 (-0.007 to 0.001)	-0.126 (-0.147 to -0.098)	0.017 (-0.01 to 0.056)	0.244
Streptococcus pneumoniae	-0.003 (-0.008 to 0)	-0.125 (-0.146 to -0.096)	0.024 (-0.001 to 0.068)	0.062
Streptococcus dysgalactiae	0 (-0.003 to 0.003)	-0.128 (-0.147 to -0.099)	0.001 (-0.022 to 0.022)	0.898
Streptococcus pasteurianus	-0.001 (-0.004 to 0.002)	-0.128 (-0.147 to -0.099)	0.006 (-0.017 to 0.034)	0.594
Streptococcus thermophilus	0 (-0.003 to 0.002)	-0.128 (-0.148 to -0.1)	0 (-0.019 to 0.024)	0.952
Streptococcus mitis	-0.004 (-0.009 to 0)	-0.124 (-0.146 to -0.096)	0.032 (0.001 to 0.076)	0.042
Streptococcus sanguinis	0.001 (-0.002 to 0.004)	-0.129 (-0.15 to -0.1)	-0.006 (-0.036 to 0.012)	0.468
Streptococcus ilei	0 (-0.002 to 0.002)	-0.128 (-0.149 to -0.101)	-0.001 (-0.019 to 0.015)	0.868
Streptococcus parasanguinis	-0.006 (-0.012 to -0.001)	-0.123 (-0.145 to -0.093)	0.041 (0.009 to 0.092)	0.008
Streptococcus oralis	-0.001 (-0.005 to 0.001)	-0.127 (-0.146 to -0.099)	0.01 (-0.009 to 0.043)	0.316
Streptococcus cristatus	-0.004 (-0.009 to -0.001)	-0.123 (-0.143 to -0.094)	0.031 (0.005 to 0.076)	0.024
Streptococcus equinus	-0.001 (-0.005 to 0.001)	-0.126 (-0.147 to -0.098)	0.01 (-0.009 to 0.043)	0.288
Streptococcus australis	0 (-0.003 to 0.002)	-0.128 (-0.149 to -0.098)	0.001 (-0.02 to 0.024)	0.898
Streptococcus koreensis	0 (-0.001 to 0.002)	-0.128 (-0.147 to -0.1)	0 (-0.018 to 0.011)	0.896
Streptococcus gordonii	-0.001 (-0.004 to 0.001)	-0.127 (-0.147 to -0.096)	0.005 (-0.009 to 0.03)	0.432
Streptococcus viridans	0 (-0.002 to 0.003)	-0.128 (-0.148 to -0.101)	-0.001 (-0.022 to 0.016)	0.808
Streptococcus infantarius	0.001 (-0.001 to 0.004)	-0.129 (-0.149 to -0.1)	-0.006 (-0.029 to 0.008)	0.396
Streptococcus gallolyticus	0 (-0.002 to 0.002)	-0.13 (-0.149 to -0.099)	-0.001 (-0.019 to 0.014)	0.832
Lactobacillus johnsonii	-0.002 (-0.005 to 0.001)	-0.125 (-0.146 to -0.095)	0.011 (-0.006 to 0.044)	0.23
Limosilactobacillus fermentum	0.001 (-0.001 to 0.005)	-0.129 (-0.149 to -0.098)	-0.008 (-0.039 to 0.007)	0.288
Limosilactobacillus mucosae	0 (-0.002 to 0.002)	-0.127 (-0.147 to -0.096)	0 (-0.014 to 0.015)	0.956
Bacillus sp. FJAT-22090	-0.001 (-0.004 to 0.001)	-0.127 (-0.148 to -0.098)	0.003 (-0.011 to 0.029)	0.584
Bacillus sp. N3536	-0.001 (-0.005 to 0.002)	-0.126 (-0.147 to -0.098)	0.008 (-0.016 to 0.043)	0.446
Psychrobacillus glaciei	0 (-0.005 to 0.004)	-0.128 (-0.148 to -0.098)	0.003 (-0.035 to 0.037)	0.854
Psychrobacillus sp. AK 1817	-0.001 (-0.005 to 0.001)	-0.127 (-0.147 to -0.098)	0.008 (-0.009 to 0.037)	0.334
Sporosarcina psychrophila	-0.002 (-0.006 to 0.002)	-0.126 (-0.146 to -0.098)	0.011 (-0.017 to 0.05)	0.388
Gemella sanguinis	-0.003 (-0.007 to 0)	-0.126 (-0.145 to -0.099)	0.022 (0 to 0.061)	0.052
[Clostridium] innocuum	0.001 (-0.003 to 0.004)	-0.128 (-0.147 to -0.099)	-0.003 (-0.035 to 0.025)	0.766
Erysipelotrichaceae bacterium GAM147	-0.001 (-0.003 to 0.001)	-0.126 (-0.146 to -0.098)	0.003 (-0.008 to 0.022)	0.544
Absiella argi	-0.003 (-0.008 to 0.001)	-0.124 (-0.145 to -0.094)	0.023 (-0.009 to 0.065)	0.17
Amedibacterium intestinale	-0.006 (-0.011 to -0.002)	-0.121 (-0.142 to -0.093)	0.043 (0.013 to 0.096)	0.004
Acidaminococcus fermentans	-0.001 (-0.004 to 0.002)	-0.127 (-0.146 to -0.094)	0.005 (-0.015 to 0.035)	0.51
Megamonas funiformis	-0.002 (-0.006 to 0.001)	-0.125 (-0.147 to -0.094)	0.014 (-0.004 to 0.049)	0.148
Megamonas hypermegale	-0.004 (-0.009 to 0)	-0.124 (-0.145 to -0.094)	0.026 (0.004 to 0.068)	0.024
Veillonella atypica	-0.001 (-0.004 to 0.001)	-0.127 (-0.148 to -0.097)	0.005 (-0.01 to 0.034)	0.424
Dialister massiliensis	0 (-0.003 to 0.003)	-0.128 (-0.147 to -0.1)	0 (-0.022 to 0.022)	0.912
Finegoldia magna	-0.002 (-0.005 to 0.001)	-0.125 (-0.145 to -0.095)	0.012 (-0.008 to 0.045)	0.274
Bifidobacterium breve	-0.001 (-0.004 to 0.003)	-0.127 (-0.146 to -0.1)	0.004 (-0.021 to 0.033)	0.594
Bifidobacterium dentium	0 (-0.002 to 0.001)	-0.127 (-0.147 to -0.099)	0.001 (-0.011 to 0.02)	0.708
Actinomyces sp. oral taxon 414	-0.002 (-0.005 to 0.001)	-0.126 (-0.146 to -0.095)	0.01 (-0.005 to 0.038)	0.198
Schaalia odontolytica	-0.003 (-0.008 to 0)	-0.124 (-0.145 to -0.093)	0.024 (0 to 0.069)	0.056
Rhodococcus erythropolis	-0.001 (-0.005 to 0.001)	-0.127 (-0.147 to -0.099)	0.01 (-0.005 to 0.04)	0.22
Microbacterium oxydans	-0.001 (-0.003 to 0.001)	-0.127 (-0.148 to -0.1)	0.004 (-0.006 to 0.027)	0.434
Arthrobacter sp. YC-RL1	-0.001 (-0.004 to 0.001)	-0.126 (-0.146 to -0.098)	0.008 (-0.006 to 0.036)	0.288
Rothia mucilaginosa	-0.001 (-0.003 to 0.002)	-0.127 (-0.148 to -0.095)	0.003 (-0.012 to 0.023)	0.576
Oerskovia sp. KBS0722	-0.001 (-0.004 to 0.001)	-0.126 (-0.146 to -0.097)	0.006 (-0.009 to 0.034)	0.31
Gordonibacter urolithinfaciens	-0.001 (-0.006 to 0.003)	-0.126 (-0.147 to -0.1)	0.008 (-0.028 to 0.049)	0.574
Gordonibacter pamelaeae	-0.002 (-0.006 to 0.001)	-0.126 (-0.147 to -0.099)	0.014 (-0.008 to 0.048)	0.242
Arabia massiliensis	-0.004 (-0.011 to 0.001)	-0.123 (-0.145 to -0.095)	0.032 (-0.005 to 0.085)	0.108

Species	ACME	ADE	Proportion	P value of proportion
Adlercreutzia sp. 8CFCBH1	-0.002 (-0.007 to 0)	-0.125 (-0.145 to -0.096)	0.017 (-0.002 to 0.055)	0.078
Adlercreutzia equolifaciens	-0.002 (-0.006 to 0)	-0.125 (-0.146 to -0.094)	0.013 (-0.003 to 0.045)	0.126
Olsenella sp. GAM18	-0.001 (-0.005 to 0.002)	-0.127 (-0.148 to -0.097)	0.006 (-0.013 to 0.038)	0.396
Akkermansia muciniphila	-0.002 (-0.007 to 0)	-0.125 (-0.145 to -0.095)	0.016 (-0.003 to 0.057)	0.112
Fusobacterium varium	-0.004 (-0.009 to 0)	-0.124 (-0.143 to -0.097)	0.029 (0 to 0.071)	0.05
Fusobacterium ulcerans	-0.002 (-0.006 to 0)	-0.125 (-0.146 to -0.096)	0.015 (-0.001 to 0.048)	0.08
Fusobacterium nucleatum	-0.002 (-0.006 to 0.001)	-0.126 (-0.147 to -0.097)	0.015 (-0.006 to 0.05)	0.166
Fusobacterium mortiferum	-0.004 (-0.009 to 0)	-0.123 (-0.143 to -0.092)	0.03 (0 to 0.075)	0.046

Supplementary Table 28 Sensitivity analysis (exclusion of who received anti-hyperlipidemia therapy) Differential metabolites between dyslipidemia and control group

Metabolites	FC	log2(FC)	P value	-LOG10(p)
13-L-Hydroperoxylinoleic acid	2.1564	1.1086	0.003463605	2.460471605
21-Deoxycortisol	2.6123	1.3853	0.024054007	1.618812568
3-Hydroxybenzoic acid	2.2809	1.1896	0.037423921	1.426850716
7-Sulfocholic acid	0.41944	-1.2535	1.64E-05	4.786017226
Adipic acid	0.36919	-1.4376	0.012387538	1.907014996
Busulfan	2.1826	1.1261	0.007119502	2.147550381
Genipin	2.5417	1.3458	0.00735382	2.133487007
L-Arogenate	2.1488	1.1035	0.017381681	1.759908215
N4-Acetylcytidine	0.32228	-1.6336	0.031708721	1.498821276
Vanillylmandelic acid	3.0713	1.6188	0.045419636	1.342756349

Supplementary Table 29 Sensitivity analysis (exclusion of who received anti-hyperlipidemia therapy)
Differential metabolites between decreased HDL and control group

Metabolites	FC	log2(FC)	P value	-LOG10(p)
(2S,3S,4S,8R,9S,13R,14R,15R,16R)-3,4,8,14,15-pentahydroxy-2,13,16-trimethyl-6-methylidene-10-oxatetracyclo[7.6.1.02,7.012,16]hexadecan-11-one	0.25235	-1.9865	0.004001634	2.39776267
(plusmn)5-HETrE	2.2495	1.1696	0.036185545	1.441464877
(S)-[10]-Gingerol	2.4214	1.2759	0.006299078	2.200723027
2,6-Diamino-5-hydroxyhexanoic acid hydrochloride	0.38868	-1.3633	0.045498212	1.342005666
21-Deoxycortisol	3.944	1.9796	0.016150112	1.791824467
3,3',4,4',5,5'-hexabromo-1H,1'H-2,2'-bipyrrole	0.34535	-1.5339	0.013518985	1.869055903
4-Hydroxytamoxifen	0.3156	-1.6638	0.013623628	1.865707226
7-Sulfocholic acid	0.43159	-1.2123	0.000676555	3.169697009
8-Isoprostane	0.29718	-1.7506	0.005676479	2.245920989
bk-EABDI	0.21633	-2.2087	0.006001967	2.221706364
Caryophyllene [T(-)]	2.1273	1.089	0.010046593	1.997981175
cis-9,10-Epoxystearic acid	2.3509	1.2332	0.025643099	1.591029494
Cortexolone	2.4953	1.3192	0.004812115	2.317664004
Corticosterone	2.5006	1.3222	0.038320823	1.416565171
Corydaline	0.49432	-1.0165	0.028804423	1.540540819
Dihydroroseoside	0.41787	-1.2589	0.037588098	1.424949647
Diphenhydramine	0.40364	-1.3089	0.001872017	2.727690276
Geranic acid	2.2171	1.1487	0.035642551	1.44803122
Gibberellin A8	0.43772	-1.1919	0.012872159	1.8903486
Histamine	2.0969	1.0683	0.006177744	2.209170112
Levorphanol	0.45945	-1.122	0.044617708	1.35049274
Lipoate	0.41077	-1.2836	0.035158363	1.453971356
Methyl 4-(2,3-dihydro-1-benzofuran-5-yl)-7-(2-furyl)-2-methyl-5-oxo-1,4,5,6,7,8-hexahydro-3-quinolinecarboxylate	0.46537	-1.1035	0.040025454	1.397663735
NCGC00180087-02!5-(hydroxymethyl)-3-(1-hydroxy-4-methylhexyl)oxolan-2-one	0.27275	-1.8744	0.001020995	2.990976353
NCGC00381071-01!1,12-dihydroxy-1,6,12,17,23,28-hexazacyclotritriacontane-2,5,13,16,24,27-hexone	3.1033	1.6338	0.014571948	1.836482398
NP-013808	0.28136	-1.8295	0.006100905	2.214605717
Poly THF n5	2.6469	1.4043	0.001359644	2.866574658
Prazosin	0.49445	-1.0161	0.005971016	2.223951735
Protocatechuic acid	0.46973	-1.0901	0.047567578	1.322688964
Salsolinol	0.38893	-1.3624	0.016506588	1.782342679
Trehalose dihydrate	0.39588	-1.3369	0.012027471	1.919825698

Supplementary Table 30 Sensitivity analysis (exclusion of who received anti-hyperlipidemia therapy)
Differential metabolites between elevated LDL and control group

Metabolites	FC	log2(FC)	P value	-LOG10(p)
(R)-Laudanidine	0.45609	-1.1326	0.036328646	1.439750794
2-Deacetoxy taxinine B	2.0851	1.0601	0.028270761	1.548662496
21-Deoxycortisol	2.2362	1.161	0.031380539	1.503339606
3-Hydroxybenzoic acid	4.1702	2.0601	1.72E-05	4.764387501
3,5,7,15-tetraacetoxy-2-hydroxy-8-isobutyroyloxy-9,14-dioxojatropha-6(17),11E-diene (2)	2.0539	1.0383	0.04031777	1.3945035
Adipic acid	0.29474	-1.7625	0.025145572	1.599538479
Albendazole	2.9945	1.5823	0.03324044	1.478333241
Asperuloside	2.065	1.0462	0.002519782	2.598637077
Beta-Tyrosine	0.48031	-1.058	0.016306338	1.787643556
Busulfan	2.5501	1.3506	0.000133781	3.87360443
Enterodiol	2.1482	1.1031	0.034686342	1.459841502
Ethylmorphine	0.17313	-2.5301	0.026745348	1.57275175
Gallic acid	2.644	1.4027	0.000161202	3.792629289
Genipin	2.1741	1.1204	0.001923121	2.715993438
Glutamine	0.40779	-1.2941	0.03964854	1.401772801
Itaconic acid	0.49451	-1.0159	0.017672854	1.752693316
Lopinavir	0.45246	-1.1441	0.011488944	1.939719874
NCGC00381061-01_C30H51N5O9_Pyrrolo[1,2-d][1,4,7,10,13,16]oxapentaazacyclononadecine-1,4,7,10,14,17(11H,16H)-hexone, 16-(2,3-dihydroxypropyl)dodecahydro-	0.40846	-1.2917	0.044373056	1.352880664
NCGC00381380-01!(2R)-3-hydroxy-2-[(2-hydroxybenzoyl)amino]propanoic acid	2.6321	1.3962	0.025516959	1.593171085
NP-016455	2.9278	1.5498	0.012782455	1.893385729
Octabenzone	0.46074	-1.118	0.012801963	1.892723444
Vanillylmandelic acid	5.6308	2.4933	0.012163349	1.914946841

Supplementary Table 31 Sensitivity analysis (exclusion of who received anti-hyperlipidemia therapy)
Differential metabolites between elevated TC and control group

Metabolites	FC	log2(FC)	P value	-LOG10(p)
(2S,5S)-trans-Carboxymethylproline	3.928	1.9738	0.03883695	1.410754888
(R)-Laudanidine	0.37051	-1.4324	0.029266818	1.5336245
2-[6-(diethylamino)purin-9-yl]-5-(hydroxymethyl)oxolane-3,4-diol	0.39573	-1.3374	0.004980268	2.302747276
2-Deacetoxy taxinine B	2.1788	1.1236	0.008359765	2.07780591
2-Methyl-3-hydroxy-5-formylpyridine-4-carboxylate	2.5526	1.352	0.006625159	2.178803677
3-Hydroxybenzoic acid	4.8876	2.2891	8.52E-05	4.06945676
3-Hydroxymethylglutaric acid	2.2891	1.1948	0.029321116	1.532819511
3,5,7,15-tetraacetoxy-2-hydroxy-8-isobutyroyloxy-9,14-dioxojatropha-6(17),11E-diene (2)	2.1279	1.0894	0.009712767	2.012657031
4,4-Bis(4-hydroxyphenyl)heptane	2.2781	1.1878	0.042584311	1.370750377
6-Hydroxynicotinic acid	2.1946	1.1339	0.005807765	2.235990956
Adipic acid	0.24276	-2.0424	0.024201619	1.616155575
Asperuloside	2.9027	1.5374	0.00047411	3.324121068
Beta-Tyrosine	0.36965	-1.4358	0.004213611	2.375345549
Bioresmethrin	0.41996	-1.2517	0.017560622	1.755460096
Busulfan	3.0114	1.5904	6.88E-05	4.162494595
CAY10498	0.46688	-1.0989	0.028169113	1.550226823
Citalopram	0.37751	-1.4054	0.013166716	1.880522536
Citrinin	2.27	1.1827	0.008164853	2.088051648
Deoxyadenosine	2.023	1.0165	0.006829363	2.165619786
Dipropyleneglycol dibenzoate	0.48952	-1.0306	0.012369418	1.907650737
Ecgonine	0.35962	-1.4755	0.049201205	1.30802426
Enterodiol	2.1192	1.0835	0.041877224	1.378022119
Galacturonic acid	2.1504	1.1046	0.004218071	2.374886073
Gallic acid	3.3721	1.7537	2.63E-05	4.579818668
Ganoderic acid G	2.23	1.157	0.014695691	1.83281
Genipin	2.7308	1.4493	0.000166581	3.778373243
Glutamine	0.299	-1.7418	0.022366736	1.650397385
Homovanillic acid	2.009	1.0065	0.016421403	1.784589733
Indinavir	0.43384	-1.2048	0.004223847	2.374291859
Itaconic acid	0.42674	-1.2286	0.015465838	1.81062653
L-Arogenate	2.1982	1.1363	0.003626608	2.440499374
Lopinavir	0.30665	-1.7053	0.003676434	2.43457325
N-(4-isopropylphenyl)-2-{[4-(3-methoxypropyl)-5-(2-methyl-1,3-thiazol-4-yl)-4H-1,2,4-triazol-3-yl]thio}acetamide	0.42771	-1.2253	0.007560732	2.121436168
N-acetyl-2-carboxy Benzenesulfonamide	0.26689	-1.9057	0.025031282	1.601516909
N1-Benzyl-2-[(2-oxo-3-piperidyl)carbonyl]hydrazine-1-carbothioamide	2.1904	1.1312	0.003874873	2.411742488
NCGC00381061-01_C30H51N5O9_Pyrrolo[1,2-d][1,4,7,10,13,16]oxapentaazacyclononadecine-1,4,7,10,14,17(11H,16H)-hexone, 16-(2,3-dihydroxypropyl)dodecahydro-	0.32426	-1.6248	0.01906657	1.719727423
NCGC00381220-01!16-butan-2-yl-3-(2,3-dihydroxypropyl)-10,11,14-trimethyl-13-propan-2-yl-4-oxa-1,8,11,14,17-pentazabicyclo[17.3.0]docosane-2,5,9,12,15,18-hexone	0.33588	-1.574	0.03513425	1.454269313
NCGC00381380-01!(2R)-3-hydroxy-2-[(2-hydroxybenzoyl)amino]propanoic acid	3.3539	1.7458	0.005372226	2.269845734
NCGC00384635-01_C27H34O8_Methyl [(1S,3S,7R,8R,9R,12S,13S)-13-(3-furyl)-6,6,8,12-tetramethyl-17-methylene-5,15-dioxo-2,14-dioxatetracyclo[7.7.1.0~1,12~.0~3,8~]heptadec-7-yl](hydroxy)acetate	2.3788	1.2502	0.042553888	1.371060756
Neamine	0.4202	-1.2509	0.017173815	1.765133224
NP-016455	3.5672	1.8348	0.002453999	2.610125575
NP-017667	2.3466	1.2306	0.002378785	2.6236449
Octabenzone	0.3043	-1.7164	0.004433318	2.353271132
Pleiomutinine	0.39194	-1.3513	0.026634132	1.574561454
Probucol	2.0141	1.0101	0.014034228	1.852811464
Quinate	3.4658	1.7932	0.014990259	1.824190864
Ribose 1-phosphate	2.0012	1.0009	0.004052145	2.392314976
Thalsimine	2.6489	1.4054	0.005783087	2.237840294
Tranexamic Acid	0.47416	-1.0766	0.04194885	1.377279936
Vanillylmandelic acid	6.7246	2.7494	0.00659304	2.180914302

Supplementary Table 32 Sensitivity analysis (exclusion of who received anti-hyperlipidemia therapy)
Differential metabolites between elevated TG and control group

Metabolites	FC	log2(FC)	P value	-LOG10(p)
(2E)-N-(4-acetamidobutyl)-3-(4-hydroxy-3-methoxyphenyl)prop-2-enamide	0.37336	-1.4214	0.003033761	2.518018587
13-L-Hydroperoxylinoleic acid	3.6203	1.8561	0.000478194	3.320395473
2-[6-(diethylamino)purin-9-yl]-5-(hydroxymethyl)oxolane-3,4-diol	0.42849	-1.2226	0.006600046	2.180453064
2,6-Diamino-5-hydroxyhexanoic acid hydrochloride	0.49784	-1.0063	0.049029595	1.309541698
20-HETE	2.539	1.3443	0.042060128	1.376129406
3,4-Dihydroxybenzeneacetic acid	2.1807	1.1248	0.007832652	2.106091167
3alpha,12alpha-Dihydroxy-5beta-chol-6-enoate	2.3815	1.2519	0.000652941	3.185125842
7-Sulfocholic acid	0.4017	-1.3158	0.00305734	2.514656225
9(S)-HPODE	2.4699	1.3045	0.002910983	2.535960364
Beta-Tyrosine	0.47023	-1.0886	0.008985144	2.046474964
Bioresmethrin	0.40169	-1.3159	0.002649517	2.576833282
Busulfan	2.4489	1.2921	0.027490988	1.560809658
CAY10498	0.32164	-1.6365	0.000512192	3.29056705
Cinolazepam	2.1041	1.0732	0.000728111	3.137802601
Citalopram	0.48569	-1.0419	0.004350049	2.361505894
Cortisone acetate	0.31656	-1.6594	0.024121103	1.617602843
Cycloposine	2.0107	1.0077	0.000119892	3.921208964
D-Maltose	2.0957	1.0675	0.000667063	3.175833063
Daidzein	2.225	1.1538	0.017717219	1.751604442
DIHYDROCELASTRYL DIACETATE	2.4243	1.2776	3.81E-05	4.418593099
Dipropyleneglycol dibenzoate	0.45842	-1.1253	0.014336974	1.843542516
dTDP-D-glucose	2.0749	1.053	0.044327326	1.353328464
Ecgonine	0.29203	-1.7758	0.039031139	1.408588777
Gallic acid	2.2284	1.156	0.003264505	2.486182708
Genipin	4.1884	2.0664	0.001746349	2.757869074
Glycocholic acid	2.0497	1.0354	0.003860782	2.413324708
Indinavir	0.48955	-1.0305	0.028785651	1.540823937
Kenpaullone	0.49978	-1.0006	0.03663397	1.436116015
L-Arogenate	3.3815	1.7577	0.003911242	2.407685261
L-Carnitine	2.2903	1.1955	0.046984814	1.328042489
Lipoxin A4	2.4435	1.289	0.032249777	1.491473283
MLS001049060-01!N-[2-(1H-indol-3-yl)ethyl]hexadecanamide	2.0958	1.0675	0.000303411	3.517968424
N2,N5-Dibenzylpyrrolidine-2,5-dicarboxamide	0.45621	-1.1322	0.0039451	2.403941957
NCGC00381071-01!1,12-dihydroxy-1,6,12,17,23,28-hexazacyclotritriacontane-2,5,13,16,24,27-hexone	2.1278	1.0893	0.020701876	1.683990303
Neamine	0.40354	-1.3092	0.015445899	1.811186819
NP-017667	2.4075	1.2675	0.028687173	1.542312243
Octabenzone	0.25566	-1.9677	0.002620681	2.581585891
PC(18_0e_20-HDoHE)	2.1946	1.1339	0.019294839	1.71455884
PGF2alpha diethyl amide	2.963	1.567	0.022161614	1.65439861
Porphobilinogen	3.0275	1.5981	0.000108307	3.965343986
Strychnopentamine	0.38981	-1.3592	0.047215971	1.325911073
Taurohyocholate	2.263	1.1782	0.003523365	2.45304234
Ursolic acid	2.4919	1.3172	0.021393327	1.669721663
vanillylmandelate	0.37564	-1.4126	0.001979133	2.703525032
Vanillylmandelic acid	5.1278	2.3583	0.031622892	1.499998419

Supplementary Table 33 Sensitivity analysis (exclusion of other ethnicity)
Differential gut microbiota between dyslipidemia and control group

Species	coef	stderr	pval	qval
Bacteroides intestinalis	-0.4247294	0.1775654	0.0169756	0.131855
Butyricimonas faecalis	-0.3580013	0.1767977	0.043223	0.2100273
Odoribacter splanchnicus	-0.4528574	0.1895533	0.0171142	0.131855
Alistipes shahii	-0.5602791	0.2320855	0.0159733	0.131855
Alistipes communis	-0.5657719	0.2028563	0.0053987	0.0875766
Alistipes sp. dk3624	-0.4488814	0.1870937	0.0166335	0.131855
Alistipes finegoldii	-0.4119317	0.1911924	0.0314641	0.1723033
Alistipes onderdonkii	-0.5843719	0.2376145	0.0141094	0.131855
Muribaculum gordoncarteri	-0.304273	0.1481958	0.0403442	0.2017208
Muribaculum intestinale	-0.2782414	0.1444644	0.0544172	0.2346742
Barnesiella viscericola	-0.3407711	0.1716172	0.04741	0.2156034
Aeromonas veronii	-0.3926901	0.1011759	0.0001149	0.009908
Escherichia coli	0.62988588	0.2290902	0.0060923	0.0875766
Escherichia albertii	0.42646791	0.1641242	0.0095214	0.1173176
Escherichia fergusonii	0.400364	0.174004	0.0216359	0.1492876
Escherichia marmotae	0.52147973	0.1594685	0.0011169	0.0350306
Psychrobacter sp. P11G5	-0.5060291	0.2211224	0.0223436	0.1511482
Haemophilus parainfluenzae	-0.5449555	0.2415955	0.0243686	0.155688
Brevundimonas diminuta	-0.3646471	0.1847535	0.048966	0.2165802
Brevundimonas naejangsanensis	-0.5516926	0.1792978	0.002182	0.0500878
Lachnospiraceae bacterium Choco86	0.20484995	0.0825711	0.0133232	0.131855
Lachnospiraceae bacterium	0.38588077	0.0939115	4.34E-05	0.0074845
Lachnospiraceae bacterium KGMB03038	0.17775709	0.0759447	0.0194716	0.139952
[Ruminococcus] gnavus	0.70476855	0.1323317	1.27E-07	4.39E-05
Blautia argi	0.32886595	0.095528	0.0006098	0.0262983
Blautia producta	0.17063326	0.0851878	0.0454776	0.2149283
Anaerostipes rhamnosivorans	0.19627998	0.0814256	0.0161993	0.131855
[Clostridium] hylemonae	0.1960193	0.0776826	0.0117976	0.1271928
[Clostridium] scindens	0.21699721	0.0819449	0.008238	0.1052638
Lachnoclostridium phocaeense	0.16028562	0.0739853	0.0305405	0.1699431
Lachnospira eligens	-0.3517831	0.1323565	0.0080048	0.1052638
Lacrimispora sphenoides	0.17194089	0.0803235	0.0325746	0.1732002
Anaerotignum propionicum	0.16702789	0.0752225	0.0266607	0.1642491
Ruminococcus sp. JE7A12	-0.3473579	0.1639211	0.0343974	0.1771208
Ruminococcus bicirculans	-0.5479279	0.1885135	0.003744	0.0728955
Ruminococcus albus	-0.2815528	0.09761	0.0040145	0.0728955
Ruminococcus champanellensis	-0.2743284	0.128197	0.0326319	0.1732002
Oscillibacter valericigenes	-0.2743795	0.1253289	0.0288336	0.1666278
Ethanoligenens harbinense	-0.2309046	0.0984115	0.0191769	0.139952
Clostridioides difficile	0.16115431	0.0664097	0.0154356	0.131855
Mogibacterium diversum	0.22212507	0.1013414	0.0286709	0.1666278
[Eubacterium] sulci	0.17419441	0.0883504	0.0489628	0.2165802
Clostridium sp. DL-VIII	0.17355854	0.0813539	0.0332959	0.1740465
Clostridium perfringens	0.25498796	0.1068733	0.017269	0.131855
Clostridium pasteurianum	0.13420817	0.0651137	0.0396874	0.201355
Clostridium beijerinckii	0.17847459	0.081832	0.0294617	0.1666278
Clostridium bornimense	0.17278183	0.0857511	0.0443599	0.2125578
Clostridium chauvoei	0.25541415	0.1279431	0.0463306	0.2156034

Species	coef	stderr	pval	qval
Hungatella hathewayi	0.17420704	0.0852756	0.0413567	0.2038296
Eubacterium limosum	0.22993612	0.0989599	0.0203732	0.1434441
Eubacterium callanderi	0.23593502	0.1042442	0.0238557	0.155688
Enterococcus sp. FDAARGOS_375	-0.2635503	0.1352899	0.0517722	0.2260936
Streptococcus salivarius	0.44586557	0.1790598	0.0129551	0.131855
Streptococcus vestibularis	0.51015354	0.1670314	0.0023229	0.0500878
Streptococcus sp. LPB0220	0.60870495	0.1745457	0.0005127	0.0252667
Streptococcus sp. HSISM1	0.57746142	0.17135	0.0007851	0.0300941
Streptococcus sp. oral taxon 431	0.28580616	0.1303889	0.0287742	0.1666278
Streptococcus sp. FDAARGOS_192	0.45290192	0.1832668	0.013649	0.131855
Streptococcus pneumoniae	0.3025525	0.0932889	0.0012294	0.0353466
Streptococcus pasteurianus	0.34284552	0.1355528	0.0116836	0.1271928
Streptococcus mitis	0.49096052	0.1248557	9.08E-05	0.009908
Streptococcus parasanguinis	0.58248499	0.1643928	0.0004156	0.0252667
Streptococcus oralis	0.31244897	0.121322	0.0101819	0.1211301
Streptococcus cristatus	0.34363705	0.1094338	0.0017442	0.0429827
Sporosarcina psychrophila	-0.3583862	0.1599302	0.0252773	0.1585576
Gemella sanguinis	0.27085465	0.123866	0.0290328	0.1666278
[Clostridium] innocuum	0.27742654	0.1156303	0.0167932	0.131855
Absiella argi	0.28455711	0.1030419	0.0058699	0.0875766
Amedibacterium intestinale	0.45680422	0.1374758	0.0009274	0.031996
Megamonas funiformis	0.98619123	0.3413615	0.0039577	0.0728955
Megamonas hypermegale	0.52597911	0.1661144	0.0015956	0.0423458
Finegoldia magna	0.2228447	0.0809822	0.0060588	0.0875766
Schaalia odontolytica	0.24590928	0.1282251	0.0554541	0.2361935
Arthrobacter sp. YC-RL1	-0.4255868	0.1594251	0.0077339	0.1052638
Oerskovia sp. KBS0722	-0.3395152	0.1502156	0.0240663	0.155688
Gordonibacter urolithinfaciens	-0.2795865	0.1408788	0.0474952	0.2156034
Arabia massiliensis	-0.3328574	0.1399304	0.0175807	0.131855
Fusobacterium varium	0.59054589	0.1690527	0.0005005	0.0252667
Fusobacterium ulcerans	0.50766441	0.1800622	0.004919	0.0848525
Fusobacterium nucleatum	0.20436753	0.0849794	0.0163816	0.131855
Fusobacterium mortiferum	0.59816089	0.233811	0.0106831	0.1228551

Supplementary Table 34 Sensitivity analysis (exclusion of other ethnicity)
Differential gut microbiota between decreased HDL and control group

Species	coef	stderr	pval	qval
Megamonas funiformis	1.646985293	0.514693472	0.001437922	0.248041547
Megamonas hypermegale	0.888089074	0.251177809	0.000493858	0.170381171

Supplementary Table 35 Sensitivity analysis (exclusion of other ethnicity)
Differential gut microbiota between elevated TC and control group

Species	coef	stderr	pval	qval
Escherichia coli	1.052351677	0.353212593	0.002992909	0.118670395
Escherichia fergusonii	0.699153597	0.268471907	0.009411299	0.249761406
Escherichia marmotae	0.705344971	0.247739603	0.004545755	0.142571392
Citrobacter tructae	0.938605401	0.292690876	0.001409757	0.081061028
[Ruminococcus] gnavus	0.747140663	0.200172721	0.000205606	0.061606617
Streptococcus pneumoniae	0.489414424	0.14112666	0.000557943	0.062341721
Streptococcus mitis	0.687355149	0.191564555	0.00035714	0.061606617
Streptococcus parasanguinis	0.678112347	0.258542425	0.008916858	0.249761406
Streptococcus oralis	0.624085801	0.187435186	0.000916948	0.06326944
Streptococcus cristatus	0.568512028	0.16738685	0.000722803	0.062341721
Megamonas funiformis	1.569004652	0.528503499	0.003095749	0.118670395
Fusobacterium varium	0.724769292	0.252841578	0.004279836	0.142571392
Fusobacterium mortiferum	1.132901027	0.361427353	0.001797494	0.088590754

Supplementary Table 36 Sensitivity analysis (exclusion of other ethnicity)
Differential gut microbiota between elevated TG and control group

Species	coef	stderr	pval	qval
Bacteroides uniformis	-0.5361208	0.2552257	0.0360085	0.1120008
Bacteroides cellulosilyticus	-0.4699099	0.220594	0.0334776	0.1089601
Bacteroides xylanisolvens	-0.391707	0.2204849	0.0761543	0.189016
Bacteroides intestinalis	-0.6389441	0.2181937	0.0035169	0.0241887
Bacteroides helcogenes	-0.3200448	0.2042071	0.1174695	0.2485427
Bacteroides heparinolyticus	-0.3338258	0.2064731	0.1065483	0.2341347
Bacteroides zoogleoformans	-0.3276475	0.1991844	0.1003929	0.2263761
Tannerella forsythia	-0.4037219	0.1772963	0.0230576	0.0846265
Tannerella sp. oral taxon HOT-286	-0.3604899	0.1908713	0.0593225	0.1583497
Paraprevotella xylaniphila	-0.4945059	0.2281083	0.0304781	0.1041084
Prevotella intermedia	-0.4708357	0.1980423	0.0176793	0.0676044
Prevotella ruminicola	-0.4244137	0.2472303	0.0864447	0.2028803
Prevotella dentalis	-0.6283656	0.2448577	0.0104703	0.0501702
Prevotella fusca	-0.3758409	0.2391871	0.1165239	0.2481527
Prevotella denticola	-0.4894965	0.2335609	0.036429	0.1122144
Prevotella oris	-0.4803272	0.2528842	0.0578933	0.1572691
Prevotella jejuni	-0.3323954	0.2130857	0.1192055	0.2492479
Prevotella sp. oral taxon 299	-0.4780449	0.2211862	0.0309855	0.1048039
Prevotella enoeca	-0.6210487	0.2321554	0.0076298	0.0417823
Pseudoprevotella muciniphila	-0.3774637	0.1906774	0.0481083	0.1371682
Butyricimonas faecalis	-0.6707305	0.214912	0.0018884	0.0178361
Odoribacter splanchnicus	-0.8205169	0.2302025	0.0003896	0.0079073
Alistipes shahii	-0.9197264	0.2805729	0.0010928	0.0136178
Alistipes megaguti	-0.7384687	0.2536054	0.0037336	0.0247712
Alistipes communis	-0.9031664	0.244063	0.0002306	0.0049731
Alistipes sp. dk3624	-0.6637917	0.2271301	0.0035757	0.0241887
Alistipes finegoldii	-0.6802542	0.2314284	0.0033886	0.0241887
Alistipes onderdonkii	-0.900934	0.289605	0.0019352	0.0178361
Alistipes dispar	-0.5934169	0.2371656	0.0125549	0.0548283
Muribaculum gordoncarteri	-0.5753166	0.1822005	0.0016533	0.0172849
Muribaculum intestinale	-0.5385982	0.1756176	0.0022394	0.0193152
Duncaniella dubosii	-0.46749	0.1706277	0.0063528	0.0359295
Petrimonas mucosa	-0.4334563	0.181281	0.0170412	0.0675774
Proteiniphilum saccharofermentans	-0.3165871	0.1796199	0.0783795	0.1931496
Barnesiella viscericola	-0.6265504	0.2072299	0.0025879	0.0202913
Porphyromonas gingivalis	-0.4871018	0.1710772	0.0045733	0.0282015
Porphyromonas asaccharolytica	-0.6708943	0.2043514	0.0010851	0.0136178
Pseudomonas versuta	-0.573676	0.326225	0.0790606	0.1934462
Pseudomonas taetrolens	-0.820709	0.3427594	0.0168909	0.0675774
Pseudomonas stutzeri	-0.2742031	0.1680957	0.1032565	0.2313213
Aeromonas veronii	-0.5112147	0.1275213	6.96E-05	0.0020005
Escherichia coli	0.82699759	0.2794588	0.0031817	0.0238625
Escherichia albertii	0.51563806	0.1996637	0.0099981	0.0492762
Escherichia fergusonii	0.51259077	0.2112054	0.0154631	0.0635091
Escherichia marmotae	0.64217325	0.1944035	0.0010008	0.0136178
Lelliottia sp. WB101	-0.3395759	0.190752	0.0754966	0.1887415
Psychrobacter sp. P11G5	-0.4708206	0.2734298	0.0854936	0.2020226
Haemophilus parainfluenzae	-0.4721799	0.2977695	0.1132886	0.2430468

Species	coef	stderr	pval	qval
Alcaligenes faecalis	-0.2935186	0.1351809	0.0302717	0.1041084
Comamonas kerstersii	-0.5444238	0.231129	0.018916	0.0709351
Oxalobacter formigenes	-0.3790929	0.2180289	0.082509	0.198568
Sutterella megalosphaeroides	-0.4977921	0.2404625	0.0387747	0.1183829
Sutterella faecalis	-0.4502792	0.2227592	0.04359	0.1263744
Brevundimonas diminuta	-0.6411552	0.2288952	0.0053328	0.0317208
Brevundimonas naejangsanensis	-0.8516193	0.2209562	0.000135	0.0033264
Desulfovibrio piger	-0.3980501	0.2217294	0.0730567	0.183975
Campylobacter jejuni	0.33031948	0.1344732	0.0142711	0.0600429
Lachnospiraceae bacterium Choco86	0.32159794	0.1015589	0.0016148	0.0172849
Lachnospiraceae bacterium	0.55548269	0.1143332	1.44E-06	0.0002477
Lachnospiraceae bacterium KGMB03038	0.26537284	0.0933879	0.0046089	0.0282015
[Ruminococcus] gnavus	0.9444904	0.1605942	6.12E-09	2.11E-06
Blautia sp. SC05B48	0.24974213	0.132099	0.0591189	0.1583497
Blautia argi	0.53653406	0.1168739	5.45E-06	0.0003758
Blautia producta	0.28935885	0.1050185	0.0060047	0.0351123
Anaerostipes hadrus	0.24851229	0.1544831	0.1081698	0.2361935
Anaerostipes rhamnosivorans	0.30183817	0.0992718	0.0024425	0.0199443
Anaerobutyricum hallii	0.27363026	0.1386291	0.0487757	0.1379313
[Clostridium] hylemonae	0.29177799	0.0961313	0.0024858	0.0199443
[Clostridium] scindens	0.33588397	0.1010249	0.0009274	0.0134613
Lachnoclostridium sp. YL32	0.29326629	0.1127495	0.0094745	0.0478519
Lachnoclostridium phocaeense	0.23988814	0.0904876	0.0081914	0.0428564
Lachnoclostridium phytofermentans	0.17594916	0.0874053	0.0444859	0.127897
Enterocloster bolteae	0.36444262	0.1463701	0.0130986	0.0564877
Enterocloster clostridioformis	0.22952011	0.1238462	0.064232	0.1666168
Lachnospira eligens	-0.5084236	0.1600804	0.0015535	0.0172849
Lacrimispora sphenoides	0.2882123	0.0983385	0.0034821	0.0241887
Lacrimispora saccharolytica	0.24047338	0.1007927	0.0172852	0.0676044
Anaerocolumna sedimenticola	0.18023631	0.0835951	0.031392	0.1051479
Lachnoanaerobaculum umeaense	0.1573824	0.0857484	0.0668372	0.1720807
Anaerotignum propionicum	0.2248033	0.091324	0.0140743	0.0599461
Faecalibacterium prausnitzii	-0.5783858	0.1815742	0.001505	0.0172849
Ruminococcus sp. JE7A12	-0.4657371	0.1960104	0.017752	0.0676044
Ruminococcus bicirculans	-0.6286472	0.2289592	0.0061808	0.0355398
Ruminococcus albus	-0.3668478	0.1195267	0.0022226	0.0193152
Ruminococcus champanellensis	-0.4099648	0.156497	0.0089773	0.0462265
Oscillibacter sp. PEA192	-0.4299002	0.1684295	0.0108937	0.0514841
Oscillibacter valericigenes	-0.4756709	0.1531306	0.0019646	0.0178361
Caproiciproducens sp. NJN-50	-0.1897362	0.1054934	0.0724848	0.1838768
Ethanoligenens harbinense	-0.4000041	0.1204104	0.0009364	0.0134613
Clostridioides difficile	0.28263859	0.0809014	0.0005042	0.0090312
Peptacetobacter hiranonis	0.24829312	0.1212066	0.0408907	0.120575
Massilistercora timonensis	0.22314572	0.1284755	0.0828806	0.198568
Flintibacter sp. KGMB00164	-0.2010837	0.126881	0.1134219	0.2430468
Mogibacterium diversum	0.38789812	0.123504	0.0017551	0.0178092
[Eubacterium] sulci	0.34432187	0.1068741	0.0013285	0.0158044
Intestinimonas butyriciproducens	-0.3921493	0.1613159	0.0152891	0.0635091
Clostridium sp. DL-VIII	0.25089474	0.0965691	0.0095704	0.0478519
Clostridium perfringens	0.33715859	0.1270629	0.0081986	0.0428564

Species	coef	stderr	pval	qval
Clostridium botulinum	0.19412799	0.0956252	0.0427943	0.1251189
Clostridium butyricum	0.20733095	0.1145795	0.0708117	0.1809632
Clostridium pasteurianum	0.16152097	0.0787446	0.0405883	0.120575
Clostridium saccharoperbutylacetonicum	0.20867489	0.092434	0.0243375	0.0874627
Clostridium beijerinckii	0.29326116	0.0979796	0.0028856	0.0221228
Clostridium bornimense	0.24376858	0.1008414	0.0159026	0.064546
Clostridium baratii	0.28415617	0.1307854	0.0301272	0.1041084
Clostridium isatidis	0.2350055	0.1371897	0.0872122	0.2032987
Clostridium saccharobutylicum	0.20670641	0.1078361	0.05581	0.1528132
Clostridium chauvoei	0.31420816	0.1521215	0.039237	0.1187436
Clostridium septicum	0.28350014	0.152197	0.0630988	0.1649174
Clostridium taeniosporum	0.2007393	0.1265501	0.1130983	0.2430468
Hungatella hathewayi	0.2663856	0.1048127	0.0112333	0.0516733
Eubacterium limosum	0.32932395	0.1217044	0.0069641	0.0387516
Eubacterium callanderi	0.36872812	0.1273653	0.0038993	0.0253822
Enterococcus faecium	0.39929079	0.1880871	0.0340833	0.1098947
Streptococcus salivarius	0.76223974	0.2177008	0.0004906	0.0090312
Streptococcus vestibularis	0.83416062	0.2025391	4.23E-05	0.0013266
Streptococcus sp. LPB0220	0.91281009	0.2132098	2.11E-05	0.0008101
Streptococcus sp. HSISM1	0.8895648	0.2091748	2.39E-05	0.0008257
Streptococcus sp. A12	0.29327236	0.169585	0.0841525	0.2002249
Streptococcus sp. oral taxon 431	0.47175852	0.1542207	0.0023425	0.0197109
Streptococcus sp. FDAARGOS_192	0.74347073	0.2225985	0.0008792	0.0134613
Streptococcus pneumoniae	0.42975911	0.1096788	9.77E-05	0.0025922
Streptococcus pasteurianus	0.52010314	0.1667807	0.0019013	0.0178361
Streptococcus thermophilus	0.30850807	0.1856118	0.0969006	0.2199389
Streptococcus mitis	0.70254675	0.1475223	2.30E-06	0.0002647
Streptococcus sanguinis	0.28810077	0.1348133	0.0329125	0.1089601
Streptococcus ilei	0.29590916	0.1828755	0.1060564	0.2341347
Streptococcus parasanguinis	0.8839585	0.1999389	1.13E-05	0.0006474
Streptococcus oralis	0.40875501	0.1439905	0.0046594	0.0282015
Streptococcus cristatus	0.48704883	0.1298047	0.0001886	0.0043387
Streptococcus equinus	0.36490027	0.1652522	0.0275311	0.0969208
Streptococcus australis	0.35263143	0.1593302	0.0271782	0.0966647
Streptococcus gordonii	0.30652744	0.1450739	0.0349356	0.1105761
Streptococcus viridans	0.32900547	0.1705545	0.0540971	0.1504231
Lactobacillus johnsonii	0.29284063	0.1148952	0.0110479	0.051507
Carnobacterium maltaromaticum	0.21020692	0.1242999	0.0912408	0.2098538
Aerococcus urinaeequi	0.22619193	0.1445093	0.1181478	0.2485427
Bacillus sp. N3536	-0.5232117	0.2472638	0.0346676	0.1105761
Psychrobacillus glaciei	-0.376935	0.1587607	0.0178319	0.0676044
Psychrobacillus sp. AK 1817	-0.504744	0.2683348	0.0603493	0.1589351
Sporosarcina psychrophila	-0.4379522	0.1928597	0.0234357	0.0851087
Gemella sanguinis	0.48781907	0.1489338	0.0011052	0.0136178
[Clostridium] innocuum	0.40416936	0.1404313	0.0041135	0.0262804
Absiella argi	0.54614659	0.125484	1.53E-05	0.0007268
Amedibacterium intestinale	0.78077053	0.1674538	3.69E-06	0.0003184
Megamonas funiformis	1.04489262	0.4142967	0.0118685	0.0532071
Megamonas hypermegale	0.67782283	0.2018643	0.0008246	0.0134613
Dialister massiliensis	-0.6197585	0.3207075	0.053671	0.1504231

Species	coef	stderr	pval	qval
Finegoldia magna	0.24469203	0.0970164	0.0118752	0.0532071
Bifidobacterium breve	0.38572745	0.1651021	0.0197338	0.0732061
Bifidobacterium pseudocatenulatum	0.65085413	0.3814861	0.0884126	0.2047137
Actinomyces sp. oral taxon 414	0.28980864	0.1504863	0.0545011	0.1504231
Schaalia odontolytica	0.53539794	0.1536962	0.0005236	0.0090312
Rhodococcus erythropolis	-0.3965372	0.2380017	0.096112	0.2195937
Arthrobacter sp. YC-RL1	-0.418989	0.1965781	0.0333741	0.1089601
Rothia mucilaginosa	0.27082574	0.1669548	0.1051855	0.2341226
Oerskovia sp. KBS0722	-0.3546081	0.1879989	0.059668	0.1583497
Gordonibacter pamelaeae	-0.3349589	0.1910801	0.0800316	0.194443
Arabia massiliensis	-0.4328327	0.1720047	0.0120619	0.0533508
Adlercreutzia sp. 8CFCBH1	-0.6239444	0.2970544	0.0360351	0.1120008
Adlercreutzia equolifaciens	-0.7361162	0.358476	0.0404067	0.120575
Fusobacterium varium	0.89491455	0.206643	1.69E-05	0.0007268
Fusobacterium ulcerans	0.56875206	0.21427	0.0081135	0.0428564
Fusobacterium nucleatum	0.26717415	0.1037724	0.0102241	0.0496803
Fusobacterium mortiferum	0.83734438	0.2836941	0.0032592	0.0239241

Supplementary Table 37 Sensitivity analysis (exclusion of other ethnicity)
Mediating effect of gut microbiota between HLBS and dyslipidemia

Species	ACME	ADE	Proportion	P value of proportion
Bacteroides intestinalis	-0.002 (-0.005 to 0)	-0.088 (-0.103 to -0.066)	0.018 (-0.003 to 0.059)	0.082
Butyricimonas faecalis	-0.001 (-0.004 to 0.001)	-0.089 (-0.104 to -0.069)	0.015 (-0.007 to 0.048)	0.19
Odoribacter splanchnicus	-0.002 (-0.005 to 0)	-0.089 (-0.103 to -0.068)	0.017 (-0.003 to 0.057)	0.102
Alistipes shahii	-0.003 (-0.007 to 0.001)	-0.088 (-0.103 to -0.066)	0.027 (-0.005 to 0.077)	0.118
Alistipes communis	-0.003 (-0.007 to 0)	-0.087 (-0.103 to -0.064)	0.034 (0 to 0.083)	0.052
Alistipes sp. dk3624	-0.002 (-0.005 to 0)	-0.088 (-0.103 to -0.068)	0.022 (-0.003 to 0.058)	0.076
Alistipes finegoldii	-0.002 (-0.005 to 0.001)	-0.089 (-0.104 to -0.067)	0.021 (-0.008 to 0.06)	0.134
Alistipes onderdonkii	-0.002 (-0.006 to 0)	-0.088 (-0.104 to -0.067)	0.024 (-0.002 to 0.066)	0.076
Muribaculum gordoncarteri	-0.001 (-0.004 to 0)	-0.089 (-0.104 to -0.068)	0.011 (-0.005 to 0.042)	0.18
Muribaculum intestinale	-0.001 (-0.003 to 0.001)	-0.09 (-0.104 to -0.07)	0.005 (-0.007 to 0.032)	0.408
Barnesiella viscericola	-0.001 (-0.003 to 0.001)	-0.089 (-0.105 to -0.068)	0.006 (-0.009 to 0.032)	0.4
Aeromonas veronii	-0.005 (-0.011 to -0.001)	-0.085 (-0.1 to -0.063)	0.055 (0.013 to 0.128)	0.004
Escherichia coli	-0.002 (-0.005 to 0)	-0.088 (-0.103 to -0.067)	0.015 (-0.005 to 0.053)	0.156
Escherichia albertii	-0.001 (-0.004 to 0)	-0.089 (-0.104 to -0.068)	0.014 (-0.002 to 0.049)	0.1
Escherichia fergusonii	-0.001 (-0.003 to 0.001)	-0.09 (-0.104 to -0.067)	0.005 (-0.013 to 0.033)	0.514
Escherichia marmotae	-0.002 (-0.006 to 0)	-0.088 (-0.103 to -0.066)	0.022 (-0.002 to 0.066)	0.072
Psychrobacter sp. P11G5	0 (-0.003 to 0.002)	-0.09 (-0.105 to -0.069)	0.003 (-0.023 to 0.033)	0.732
Haemophilus parainfluenzae	-0.002 (-0.005 to 0)	-0.089 (-0.103 to -0.067)	0.019 (-0.003 to 0.057)	0.104
Brevundimonas diminuta	0 (-0.002 to 0.001)	-0.09 (-0.105 to -0.067)	0.002 (-0.015 to 0.024)	0.714
Brevundimonas naejangsanensis	-0.001 (-0.005 to 0.001)	-0.088 (-0.103 to -0.068)	0.014 (-0.009 to 0.051)	0.184
Lachnospiraceae bacterium Choco86	0 (-0.002 to 0.003)	-0.091 (-0.105 to -0.07)	-0.003 (-0.037 to 0.022)	0.726
Lachnospiraceae bacterium	-0.003 (-0.007 to 0)	-0.086 (-0.101 to -0.064)	0.036 (0.005 to 0.085)	0.02
Lachnospiraceae bacterium KGMB0303[illegible]	0 (-0.002 to 0.002)	-0.09 (-0.105 to -0.07)	-0.002 (-0.029 to 0.026)	0.842
[Ruminococcus] gnavus	-0.006 (-0.011 to -0.002)	-0.084 (-0.099 to -0.062)	0.066 (0.021 to 0.131)	0.002
Blautia argi	-0.002 (-0.005 to 0.001)	-0.088 (-0.103 to -0.067)	0.017 (-0.008 to 0.057)	0.158
Blautia producta	0 (-0.001 to 0.002)	-0.091 (-0.106 to -0.067)	-0.004 (-0.029 to 0.016)	0.636
Anaerostipes rhamnosivorans	0 (-0.002 to 0.003)	-0.09 (-0.105 to -0.07)	-0.003 (-0.031 to 0.025)	0.782
[Clostridium] hylemonae	0 (-0.003 to 0.002)	-0.09 (-0.105 to -0.068)	0.002 (-0.021 to 0.032)	0.828
[Clostridium] scindens	0 (-0.003 to 0.002)	-0.089 (-0.104 to -0.067)	0.003 (-0.021 to 0.035)	0.746
Lachnoclostridium phocaeense	0.001 (-0.002 to 0.003)	-0.091 (-0.106 to -0.069)	-0.005 (-0.036 to 0.017)	0.642
Lachnospira eligens	-0.003 (-0.006 to 0)	-0.088 (-0.102 to -0.067)	0.026 (0 to 0.074)	0.048
Lacrimispora sphenoides	0 (-0.002 to 0.002)	-0.09 (-0.105 to -0.069)	-0.001 (-0.024 to 0.023)	0.91
Anaerotignum propionicum	0.001 (-0.002 to 0.003)	-0.09 (-0.106 to -0.071)	-0.004 (-0.038 to 0.019)	0.652
Ruminococcus sp. JE7A12	-0.001 (-0.004 to 0)	-0.089 (-0.105 to -0.068)	0.013 (-0.004 to 0.049)	0.164
Ruminococcus bicirculans	-0.003 (-0.006 to 0)	-0.088 (-0.102 to -0.065)	0.029 (0.002 to 0.075)	0.034
Ruminococcus albus	-0.003 (-0.007 to 0)	-0.087 (-0.103 to -0.065)	0.033 (0.002 to 0.081)	0.042
Ruminococcus champanellensis	-0.002 (-0.006 to 0.001)	-0.088 (-0.103 to -0.067)	0.022 (-0.006 to 0.067)	0.146
Oscillibacter valericigenes	-0.002 (-0.006 to 0.001)	-0.088 (-0.103 to -0.066)	0.023 (-0.009 to 0.071)	0.158
Ethanoligenens harbinense	-0.002 (-0.006 to 0.001)	-0.088 (-0.103 to -0.068)	0.024 (-0.006 to 0.068)	0.106
Clostridioides difficile	0 (-0.002 to 0.003)	-0.09 (-0.106 to -0.069)	0 (-0.029 to 0.022)	0.948
Mogibacterium diversum	-0.001 (-0.003 to 0)	-0.09 (-0.104 to -0.069)	0.009 (-0.006 to 0.037)	0.268
[Eubacterium] sulci	0 (-0.002 to 0.001)	-0.09 (-0.105 to -0.068)	0.001 (-0.016 to 0.022)	0.784
Clostridium sp. DL-VIII	-0.002 (-0.005 to 0.001)	-0.089 (-0.104 to -0.068)	0.016 (-0.007 to 0.055)	0.188
Clostridium perfringens	-0.002 (-0.007 to 0.002)	-0.088 (-0.103 to -0.066)	0.026 (-0.023 to 0.08)	0.316
Clostridium pasteurianum	-0.001 (-0.004 to 0.001)	-0.089 (-0.104 to -0.068)	0.013 (-0.007 to 0.048)	0.214
Clostridium beijerinckii	-0.002 (-0.005 to 0.001)	-0.088 (-0.103 to -0.068)	0.02 (-0.012 to 0.062)	0.222
Clostridium bornimense	-0.001 (-0.005 to 0.001)	-0.089 (-0.103 to -0.067)	0.014 (-0.014 to 0.052)	0.274
Clostridium chauvoei	-0.001 (-0.005 to 0.001)	-0.089 (-0.104 to -0.067)	0.014 (-0.014 to 0.057)	0.324
Hungatella hathewayi	0.001 (-0.001 to 0.004)	-0.091 (-0.105 to -0.072)	-0.006 (-0.042 to 0.013)	0.47
Eubacterium limosum	0 (-0.002 to 0.003)	-0.091 (-0.105 to -0.071)	-0.003 (-0.036 to 0.025)	0.774
Eubacterium callanderi	0.001 (-0.001 to 0.003)	-0.091 (-0.105 to -0.069)	-0.006 (-0.038 to 0.015)	0.526
Enterococcus sp. FDAARGOS_375	0 (-0.003 to 0.001)	-0.09 (-0.105 to -0.069)	0.003 (-0.014 to 0.03)	0.556
Streptococcus salivarius	-0.001 (-0.004 to 0.001)	-0.09 (-0.104 to -0.069)	0.011 (-0.008 to 0.042)	0.286
Streptococcus vestibularis	-0.002 (-0.005 to 0.001)	-0.088 (-0.104 to -0.067)	0.017 (-0.009 to 0.056)	0.174
Streptococcus sp. LPB0220	-0.003 (-0.007 to 0)	-0.088 (-0.104 to -0.068)	0.034 (0.005 to 0.077)	0.018
Streptococcus sp. HSISM1	-0.003 (-0.006 to 0)	-0.088 (-0.103 to -0.065)	0.026 (0.001 to 0.071)	0.042

Species	ACME	ADE	Proportion	P value of proportion
Streptococcus sp. oral taxon 431	0 (-0.002 to 0.002)	-0.09 (-0.105 to -0.071)	0.002 (-0.018 to 0.028)	0.724
Streptococcus sp. FDAARGOS_192	-0.001 (-0.004 to 0.001)	-0.09 (-0.105 to -0.068)	0.01 (-0.012 to 0.042)	0.328
Streptococcus pneumoniae	-0.002 (-0.006 to 0)	-0.089 (-0.103 to -0.067)	0.022 (-0.002 to 0.067)	0.072
Streptococcus pasteurianus	0 (-0.002 to 0.001)	-0.09 (-0.106 to -0.069)	0.003 (-0.015 to 0.027)	0.686
Streptococcus mitis	-0.004 (-0.008 to -0.001)	-0.086 (-0.102 to -0.065)	0.037 (0.008 to 0.091)	0.01
Streptococcus parasanguinis	-0.003 (-0.007 to -0.001)	-0.088 (-0.103 to -0.067)	0.034 (0.007 to 0.084)	0.01
Streptococcus oralis	-0.001 (-0.004 to 0.001)	-0.089 (-0.104 to -0.07)	0.01 (-0.01 to 0.042)	0.278
Streptococcus cristatus	-0.003 (-0.007 to 0)	-0.088 (-0.103 to -0.068)	0.03 (0.003 to 0.075)	0.02
Sporosarcina psychrophila	-0.001 (-0.003 to 0.002)	-0.09 (-0.105 to -0.068)	0.005 (-0.018 to 0.035)	0.596
Gemella sanguinis	-0.001 (-0.004 to 0)	-0.089 (-0.104 to -0.068)	0.014 (-0.003 to 0.051)	0.118
[Clostridium] innocuum	0.001 (-0.002 to 0.004)	-0.091 (-0.105 to -0.07)	-0.007 (-0.042 to 0.021)	0.534
Absiella argi	-0.001 (-0.003 to 0.001)	-0.09 (-0.105 to -0.068)	0.008 (-0.013 to 0.038)	0.412
Amedibacterium intestinale	-0.003 (-0.007 to 0)	-0.087 (-0.101 to -0.065)	0.032 (0.005 to 0.079)	0.022
Megamonas funiformis	-0.003 (-0.007 to 0)	-0.087 (-0.104 to -0.065)	0.033 (0.002 to 0.077)	0.034
Megamonas hypermegale	-0.003 (-0.006 to 0)	-0.087 (-0.102 to -0.065)	0.029 (0.003 to 0.075)	0.018
Finegoldia magna	-0.001 (-0.004 to 0.001)	-0.089 (-0.105 to -0.068)	0.008 (-0.016 to 0.041)	0.436
Schaalia odontolytica	-0.001 (-0.004 to 0)	-0.089 (-0.103 to -0.067)	0.011 (-0.003 to 0.043)	0.178
Arthrobacter sp. YC-RL1	-0.001 (-0.004 to 0.001)	-0.089 (-0.103 to -0.07)	0.012 (-0.009 to 0.047)	0.236
Oerskovia sp. KBS0722	-0.001 (-0.004 to 0)	-0.089 (-0.104 to -0.069)	0.013 (-0.004 to 0.044)	0.152
Gordonibacter urolithinfaciens	-0.002 (-0.005 to 0.001)	-0.089 (-0.105 to -0.066)	0.018 (-0.013 to 0.062)	0.264
Arabia massiliensis	-0.002 (-0.007 to 0.001)	-0.088 (-0.104 to -0.064)	0.026 (-0.008 to 0.077)	0.15
Fusobacterium varium	-0.003 (-0.007 to 0)	-0.087 (-0.103 to -0.064)	0.034 (0.004 to 0.082)	0.018
Fusobacterium ulcerans	-0.002 (-0.006 to 0)	-0.088 (-0.104 to -0.066)	0.024 (0 to 0.069)	0.048
Fusobacterium nucleatum	-0.002 (-0.005 to 0)	-0.088 (-0.103 to -0.067)	0.018 (-0.005 to 0.054)	0.13
Fusobacterium mortiferum	-0.002 (-0.006 to 0.001)	-0.088 (-0.103 to -0.066)	0.026 (-0.007 to 0.073)	0.112

Supplementary Table 38 Sensitivity analysis (exclusion of other ethnicity)
Mediating effect of gut microbiota between HLBS and decreased HDL

Species	ACME	ADE	Proportion	P value of proportion
Megamonas funiformis	-0.005 (-0.011 to -0.001)	-0.044 (-0.091 to -0.008)	0.098 (0.015 to 0.363)	0.012
Megamonas hypermegale	-0.005 (-0.011 to -0.001)	-0.044 (-0.091 to -0.006)	0.093 (0.016 to 0.371)	0.018

Supplementary Table 39 Sensitivity analysis (exclusion of other ethnicity)
Mediating effect of gut microbiota between HLBS and elevated TC

Species	ACME	ADE	Proportion	P value of proportion
Escherichia coli	-0.003 (-0.009 to 0)	-0.107 (-0.155 to -0.056)	0.026 (-0.002 to 0.087)	0.074
Escherichia fergusonii	-0.002 (-0.007 to 0.001)	-0.109 (-0.156 to -0.054)	0.016 (-0.01 to 0.064)	0.204
Escherichia marmotae	-0.003 (-0.008 to 0)	-0.109 (-0.158 to -0.053)	0.026 (-0.002 to 0.082)	0.07
Citrobacter tructae	-0.001 (-0.005 to 0.003)	-0.111 (-0.159 to -0.058)	0.008 (-0.031 to 0.051)	0.62
[Ruminococcus] gnavus	-0.006 (-0.012 to -0.001)	-0.103 (-0.154 to -0.046)	0.05 (0.01 to 0.141)	0.01
Streptococcus pneumoniae	-0.004 (-0.01 to 0)	-0.108 (-0.155 to -0.054)	0.034 (0 to 0.099)	0.054
Streptococcus mitis	-0.005 (-0.012 to 0)	-0.107 (-0.154 to -0.052)	0.044 (0.002 to 0.109)	0.032
Streptococcus parasanguinis	-0.004 (-0.01 to 0)	-0.11 (-0.16 to -0.056)	0.034 (0.003 to 0.101)	0.026
Streptococcus oralis	-0.002 (-0.007 to 0.002)	-0.109 (-0.159 to -0.053)	0.016 (-0.024 to 0.078)	0.352
Streptococcus cristatus	-0.004 (-0.01 to 0)	-0.108 (-0.156 to -0.052)	0.034 (0.003 to 0.095)	0.026
Megamonas funiformis	-0.005 (-0.011 to 0)	-0.106 (-0.154 to -0.053)	0.039 (0.002 to 0.113)	0.04
Fusobacterium varium	-0.004 (-0.01 to 0)	-0.107 (-0.159 to -0.055)	0.03 (-0.001 to 0.095)	0.058
Fusobacterium mortiferum	-0.005 (-0.011 to 0)	-0.105 (-0.157 to -0.056)	0.045 (0.003 to 0.119)	0.032

Supplementary Table 40 Sensitivity analysis (exclusion of other ethnicity)
Mediating effect of gut microbiota between HLBS and elevated TG

Species	ACME	ADE	Proportion	P value of proportion
Bacteroides uniformis	-0.002 (-0.005 to 0)	-0.124 (-0.143 to -0.094)	0.011 (-0.004 to 0.043)	0.18
Bacteroides cellulosilyticus	-0.002 (-0.006 to 0)	-0.124 (-0.143 to -0.096)	0.014 (-0.004 to 0.046)	0.128
Bacteroides xylanisolvens	-0.001 (-0.003 to 0.002)	-0.126 (-0.145 to -0.097)	0.003 (-0.013 to 0.025)	0.574
Bacteroides intestinalis	-0.002 (-0.006 to 0.001)	-0.124 (-0.143 to -0.095)	0.016 (-0.004 to 0.052)	0.132
Bacteroides helcogenes	-0.001 (-0.003 to 0.001)	-0.126 (-0.145 to -0.098)	0.003 (-0.008 to 0.026)	0.524
Bacteroides heparinolyticus	0 (-0.003 to 0.001)	-0.126 (-0.145 to -0.096)	0.002 (-0.011 to 0.023)	0.654
Bacteroides zoogleoformans	-0.001 (-0.003 to 0.001)	-0.125 (-0.144 to -0.1)	0.002 (-0.01 to 0.026)	0.558
Tannerella forsythia	0 (-0.003 to 0.003)	-0.126 (-0.145 to -0.099)	0.001 (-0.022 to 0.027)	0.924
Tannerella sp. oral taxon HOT-286	0 (-0.003 to 0.002)	-0.126 (-0.145 to -0.097)	0.002 (-0.018 to 0.024)	0.766
Paraprevotella xylaniphila	0.001 (-0.002 to 0.004)	-0.127 (-0.146 to -0.099)	-0.004 (-0.03 to 0.019)	0.62
Prevotella intermedia	0 (-0.003 to 0.004)	-0.127 (-0.146 to -0.098)	-0.003 (-0.038 to 0.022)	0.782
Prevotella ruminicola	0.001 (-0.002 to 0.004)	-0.127 (-0.147 to -0.1)	-0.005 (-0.039 to 0.013)	0.538
Prevotella dentalis	0.001 (-0.003 to 0.005)	-0.127 (-0.146 to -0.099)	-0.004 (-0.04 to 0.025)	0.734
Prevotella fusca	0.001 (-0.002 to 0.004)	-0.127 (-0.146 to -0.099)	-0.008 (-0.038 to 0.014)	0.424
Prevotella denticola	0.001 (-0.002 to 0.005)	-0.127 (-0.146 to -0.1)	-0.005 (-0.038 to 0.019)	0.592
Prevotella oris	0 (-0.003 to 0.003)	-0.126 (-0.146 to -0.095)	-0.001 (-0.027 to 0.026)	0.846
Prevotella jejuni	0.001 (-0.001 to 0.004)	-0.127 (-0.147 to -0.099)	-0.006 (-0.033 to 0.012)	0.468
Prevotella sp. oral taxon 299	0.001 (-0.002 to 0.005)	-0.128 (-0.146 to -0.099)	-0.007 (-0.039 to 0.016)	0.484
Prevotella enoeca	0.001 (-0.003 to 0.005)	-0.128 (-0.145 to -0.101)	-0.005 (-0.038 to 0.019)	0.69
Pseudoprevotella muciniphila	0.001 (-0.002 to 0.004)	-0.127 (-0.145 to -0.1)	-0.003 (-0.031 to 0.017)	0.716
Butyricimonas faecalis	-0.003 (-0.008 to 0)	-0.123 (-0.142 to -0.095)	0.023 (0.002 to 0.065)	0.028
Odoribacter splanchnicus	-0.003 (-0.007 to 0)	-0.123 (-0.143 to -0.091)	0.021 (-0.003 to 0.058)	0.1
Alistipes shahii	-0.005 (-0.01 to -0.001)	-0.122 (-0.141 to -0.095)	0.036 (0.007 to 0.08)	0.02
Alistipes megaguti	-0.001 (-0.004 to 0.002)	-0.126 (-0.144 to -0.099)	0.004 (-0.022 to 0.034)	0.66
Alistipes communis	-0.006 (-0.012 to -0.002)	-0.121 (-0.14 to -0.094)	0.042 (0.013 to 0.094)	0.002
Alistipes sp. dk3624	-0.003 (-0.008 to 0)	-0.123 (-0.141 to -0.094)	0.023 (0.002 to 0.065)	0.03
Alistipes finegoldii	-0.004 (-0.008 to -0.001)	-0.122 (-0.142 to -0.093)	0.028 (0.004 to 0.068)	0.006
Alistipes onderdonkii	-0.004 (-0.009 to 0)	-0.122 (-0.141 to -0.094)	0.028 (0.004 to 0.073)	0.026
Alistipes dispar	-0.002 (-0.006 to 0)	-0.124 (-0.143 to -0.095)	0.017 (0 to 0.048)	0.05
Muribaculum gordoncarteri	-0.002 (-0.007 to 0.001)	-0.124 (-0.143 to -0.097)	0.016 (-0.007 to 0.053)	0.154
Muribaculum intestinale	-0.001 (-0.005 to 0.003)	-0.125 (-0.144 to -0.097)	0.006 (-0.021 to 0.039)	0.608
Duncaniella dubosii	-0.001 (-0.004 to 0.002)	-0.126 (-0.145 to -0.097)	0.005 (-0.019 to 0.033)	0.604
Petrimonas mucosa	0.001 (-0.002 to 0.004)	-0.127 (-0.146 to -0.101)	-0.005 (-0.035 to 0.019)	0.596
Proteiniphilum saccharofermentans	0 (-0.002 to 0.003)	-0.127 (-0.146 to -0.099)	-0.002 (-0.029 to 0.017)	0.718
Barnesiella viscericola	0 (-0.004 to 0.004)	-0.125 (-0.145 to -0.097)	0.004 (-0.028 to 0.034)	0.816
Porphyromonas gingivalis	0 (-0.003 to 0.004)	-0.128 (-0.146 to -0.101)	-0.002 (-0.035 to 0.025)	0.864
Porphyromonas asaccharolytica	-0.002 (-0.007 to 0.002)	-0.124 (-0.145 to -0.094)	0.014 (-0.013 to 0.053)	0.274
Pseudomonas versuta	0 (-0.003 to 0.002)	-0.125 (-0.144 to -0.093)	0.001 (-0.014 to 0.024)	0.73
Pseudomonas taetrolens	0.002 (-0.002 to 0.006)	-0.126 (-0.145 to -0.098)	-0.009 (-0.053 to 0.017)	0.398
Pseudomonas stutzeri	-0.001 (-0.004 to 0.001)	-0.125 (-0.145 to -0.096)	0.004 (-0.009 to 0.033)	0.502
Aeromonas veronii	-0.008 (-0.016 to -0.002)	-0.116 (-0.138 to -0.086)	0.064 (0.019 to 0.14)	0
Escherichia coli	-0.002 (-0.007 to 0.001)	-0.123 (-0.142 to -0.093)	0.017 (-0.005 to 0.058)	0.116
Escherichia albertii	-0.002 (-0.006 to 0)	-0.123 (-0.143 to -0.095)	0.015 (-0.002 to 0.048)	0.11
Escherichia fergusonii	-0.001 (-0.005 to 0.001)	-0.125 (-0.144 to -0.095)	0.007 (-0.01 to 0.036)	0.416
Escherichia marmotae	-0.003 (-0.008 to 0)	-0.123 (-0.142 to -0.094)	0.023 (0.001 to 0.063)	0.038
Lelliottia sp. WB101	-0.001 (-0.005 to 0.001)	-0.125 (-0.144 to -0.099)	0.006 (-0.011 to 0.036)	0.392
Psychrobacter sp. P11G5	0 (-0.003 to 0.003)	-0.126 (-0.145 to -0.099)	-0.001 (-0.027 to 0.021)	0.894
Haemophilus parainfluenzae	-0.001 (-0.005 to 0.001)	-0.124 (-0.142 to -0.098)	0.008 (-0.011 to 0.036)	0.412
Alcaligenes faecalis	-0.001 (-0.004 to 0.001)	-0.124 (-0.145 to -0.095)	0.007 (-0.008 to 0.038)	0.308
Comamonas kerstersii	-0.003 (-0.008 to 0.001)	-0.124 (-0.142 to -0.098)	0.02 (-0.005 to 0.063)	0.134
Oxalobacter formigenes	-0.001 (-0.005 to 0.001)	-0.124 (-0.143 to -0.096)	0.01 (-0.004 to 0.038)	0.2
Sutterella megalosphaeroides	-0.001 (-0.004 to 0.001)	-0.125 (-0.143 to -0.097)	0.008 (-0.006 to 0.035)	0.328
Sutterella faecalis	-0.001 (-0.005 to 0.001)	-0.125 (-0.142 to -0.096)	0.008 (-0.006 to 0.037)	0.27
Brevundimonas diminuta	-0.001 (-0.005 to 0.002)	-0.124 (-0.143 to -0.094)	0.008 (-0.015 to 0.04)	0.412

Species	ACME	ADE	Proportion	P value of proportion
Brevundimonas naejangsanensis	-0.003 (-0.008 to 0)	-0.122 (-0.142 to -0.091)	0.022 (-0.004 to 0.064)	0.096
Desulfovibrio piger	-0.001 (-0.004 to 0.001)	-0.124 (-0.144 to -0.099)	0.008 (-0.008 to 0.035)	0.338
Campylobacter jejuni	-0.001 (-0.005 to 0.002)	-0.124 (-0.144 to -0.095)	0.007 (-0.014 to 0.038)	0.432
Lachnospiraceae bacterium Choco86	0 (-0.004 to 0.004)	-0.123 (-0.143 to -0.093)	0.001 (-0.033 to 0.034)	0.922
Lachnospiraceae bacterium	-0.006 (-0.011 to -0.001)	-0.118 (-0.138 to -0.088)	0.043 (0.008 to 0.097)	0.012
Lachnospiraceae bacterium KGMB03038	0 (-0.004 to 0.003)	-0.124 (-0.144 to -0.095)	0.002 (-0.029 to 0.032)	0.834
[Ruminococcus] gnavus	-0.008 (-0.016 to -0.003)	-0.117 (-0.138 to -0.087)	0.065 (0.02 to 0.131)	0
Blautia sp. SC05B48	0 (-0.002 to 0.003)	-0.125 (-0.145 to -0.098)	-0.001 (-0.028 to 0.018)	0.838
Blautia argi	-0.004 (-0.009 to 0)	-0.12 (-0.141 to -0.09)	0.029 (-0.003 to 0.078)	0.082
Blautia producta	0 (-0.003 to 0.003)	-0.125 (-0.145 to -0.096)	-0.001 (-0.028 to 0.027)	0.926
Anaerostipes hadrus	0.003 (0 to 0.009)	-0.128 (-0.147 to -0.099)	-0.024 (-0.073 to -0.001)	0.04
Anaerostipes rhamnosivorans	-0.001 (-0.004 to 0.003)	-0.125 (-0.144 to -0.097)	0.004 (-0.025 to 0.034)	0.75
Anaerobutyricum hallii	0.001 (-0.002 to 0.004)	-0.125 (-0.146 to -0.096)	-0.003 (-0.031 to 0.015)	0.616
[Clostridium] hylemonae	-0.001 (-0.005 to 0.002)	-0.124 (-0.144 to -0.095)	0.009 (-0.015 to 0.044)	0.388
[Clostridium] scindens	-0.001 (-0.006 to 0.002)	-0.124 (-0.144 to -0.095)	0.01 (-0.02 to 0.045)	0.424
Lachnoclostridium sp. YL32	0 (-0.003 to 0.003)	-0.125 (-0.144 to -0.094)	-0.001 (-0.027 to 0.024)	0.9
Lachnoclostridium phocaeense	0 (-0.004 to 0.004)	-0.124 (-0.144 to -0.095)	0 (-0.03 to 0.029)	0.948
Lachnoclostridium phytofermentans	0 (-0.003 to 0.003)	-0.125 (-0.143 to -0.096)	0 (-0.021 to 0.022)	0.982
Enterocloster bolteae	0 (-0.003 to 0.003)	-0.125 (-0.144 to -0.1)	-0.001 (-0.029 to 0.025)	0.914
Enterocloster clostridioformis	0.001 (-0.001 to 0.005)	-0.126 (-0.145 to -0.097)	-0.009 (-0.039 to 0.01)	0.338
Lachnospira eligens	-0.004 (-0.009 to 0)	-0.122 (-0.142 to -0.094)	0.031 (0.004 to 0.074)	0.008
Lacrimispora sphenoides	-0.001 (-0.005 to 0.002)	-0.124 (-0.143 to -0.095)	0.008 (-0.016 to 0.041)	0.47
Lacrimispora saccharolytica	0 (-0.003 to 0.002)	-0.124 (-0.145 to -0.095)	0.003 (-0.018 to 0.029)	0.716
Anaerocolumna sedimenticola	0 (-0.003 to 0.002)	-0.124 (-0.144 to -0.096)	0.001 (-0.022 to 0.027)	0.87
Lachnoanaerobaculum umeaense	0 (-0.002 to 0.003)	-0.126 (-0.145 to -0.097)	-0.001 (-0.026 to 0.019)	0.82
Anaerotignum propionicum	0 (-0.003 to 0.004)	-0.125 (-0.144 to -0.097)	-0.001 (-0.034 to 0.026)	0.942
Faecalibacterium prausnitzii	-0.005 (-0.01 to -0.001)	-0.121 (-0.14 to -0.093)	0.036 (0.006 to 0.081)	0.018
Ruminococcus sp. JE7A12	-0.002 (-0.006 to 0)	-0.124 (-0.143 to -0.096)	0.015 (-0.003 to 0.05)	0.13
Ruminococcus bicirculans	-0.004 (-0.008 to 0)	-0.122 (-0.143 to -0.092)	0.026 (0.002 to 0.069)	0.034
Ruminococcus albus	-0.004 (-0.009 to 0)	-0.123 (-0.142 to -0.093)	0.031 (0.004 to 0.078)	0.016
Ruminococcus champanellensis	-0.004 (-0.009 to 0)	-0.123 (-0.142 to -0.095)	0.029 (-0.002 to 0.077)	0.06
Oscillibacter sp. PEA192	-0.003 (-0.008 to 0)	-0.123 (-0.142 to -0.093)	0.026 (0.001 to 0.065)	0.04
Oscillibacter valericigenes	-0.005 (-0.011 to -0.001)	-0.121 (-0.141 to -0.093)	0.04 (0.006 to 0.092)	0.01
Caproiciproducens sp. NJN-50	-0.002 (-0.006 to 0.001)	-0.125 (-0.143 to -0.096)	0.013 (-0.01 to 0.047)	0.232
Ethanoligenens harbinense	-0.005 (-0.011 to -0.001)	-0.121 (-0.141 to -0.089)	0.037 (0.006 to 0.09)	0.022
Clostridioides difficile	0 (-0.005 to 0.004)	-0.125 (-0.144 to -0.094)	0.002 (-0.03 to 0.037)	0.894
Peptacetobacter hiranonis	-0.002 (-0.005 to 0.001)	-0.124 (-0.144 to -0.096)	0.01 (-0.005 to 0.041)	0.206
Massilistercora timonensis	0 (-0.003 to 0.002)	-0.125 (-0.144 to -0.097)	0 (-0.021 to 0.023)	0.91
Flintibacter sp. KGMB00164	-0.002 (-0.006 to 0.002)	-0.125 (-0.144 to -0.096)	0.012 (-0.015 to 0.044)	0.374
Mogibacterium diversum	-0.002 (-0.006 to 0.001)	-0.125 (-0.144 to -0.096)	0.014 (-0.008 to 0.052)	0.17
[Eubacterium] sulci	-0.001 (-0.004 to 0.003)	-0.125 (-0.145 to -0.092)	0.004 (-0.022 to 0.035)	0.744
Intestinimonas butyriciproducens	-0.003 (-0.009 to 0.001)	-0.122 (-0.142 to -0.094)	0.025 (-0.004 to 0.071)	0.094
Clostridium sp. DL-VIII	-0.003 (-0.007 to 0)	-0.123 (-0.143 to -0.096)	0.018 (-0.002 to 0.058)	0.092
Clostridium perfringens	-0.004 (-0.01 to 0.001)	-0.122 (-0.143 to -0.091)	0.028 (-0.01 to 0.082)	0.144
Clostridium botulinum	-0.002 (-0.006 to 0.001)	-0.124 (-0.144 to -0.091)	0.012 (-0.008 to 0.048)	0.232
Clostridium butyricum	-0.001 (-0.005 to 0.001)	-0.124 (-0.144 to -0.094)	0.008 (-0.009 to 0.036)	0.322
Clostridium pasteurianum	-0.001 (-0.005 to 0.001)	-0.125 (-0.144 to -0.094)	0.009 (-0.006 to 0.043)	0.264
Clostridium saccharoperbutylacetonicum	-0.002 (-0.007 to 0.001)	-0.124 (-0.143 to -0.094)	0.015 (-0.01 to 0.054)	0.254
Clostridium beijerinckii	-0.003 (-0.008 to 0)	-0.122 (-0.142 to -0.092)	0.024 (-0.001 to 0.07)	0.082
Clostridium bornimense	-0.002 (-0.006 to 0.001)	-0.123 (-0.143 to -0.095)	0.016 (-0.01 to 0.058)	0.236
Clostridium baratii	-0.002 (-0.006 to 0.001)	-0.124 (-0.144 to -0.097)	0.015 (-0.008 to 0.054)	0.234
Clostridium isatidis	-0.001 (-0.005 to 0.002)	-0.125 (-0.144 to -0.096)	0.009 (-0.015 to 0.042)	0.436
Clostridium saccharobutylicum	-0.001 (-0.005 to 0.001)	-0.124 (-0.144 to -0.095)	0.009 (-0.009 to 0.04)	0.308
Clostridium chauvoei	-0.002 (-0.006 to 0.001)	-0.124 (-0.145 to -0.094)	0.013 (-0.011 to 0.052)	0.266
Clostridium septicum	-0.002 (-0.007 to 0.002)	-0.124 (-0.144 to -0.095)	0.011 (-0.016 to 0.058)	0.356
Clostridium taeniosporum	-0.001 (-0.004 to 0.001)	-0.125 (-0.144 to -0.098)	0.007 (-0.011 to 0.035)	0.408
Hungatella hathewayi	0 (-0.003 to 0.003)	-0.125 (-0.144 to -0.097)	0.001 (-0.027 to 0.029)	0.936

Species	ACME	ADE	Proportion	P value of proportion
Eubacterium limosum	0 (-0.004 to 0.004)	-0.125 (-0.144 to -0.095)	-0.001 (-0.033 to 0.031)	0.892
Eubacterium callanderi	0.001 (-0.002 to 0.005)	-0.126 (-0.145 to -0.098)	-0.01 (-0.044 to 0.016)	0.422
Enterococcus faecium	-0.002 (-0.005 to 0.001)	-0.124 (-0.143 to -0.097)	0.01 (-0.005 to 0.044)	0.18
Streptococcus salivarius	-0.003 (-0.008 to 0.002)	-0.124 (-0.143 to -0.097)	0.019 (-0.013 to 0.061)	0.252
Streptococcus vestibularis	-0.003 (-0.008 to 0.002)	-0.124 (-0.143 to -0.095)	0.019 (-0.017 to 0.065)	0.254
Streptococcus sp. LPB0220	-0.006 (-0.012 to -0.001)	-0.121 (-0.141 to -0.093)	0.045 (0.009 to 0.1)	0.01
Streptococcus sp. HSISM1	-0.005 (-0.01 to 0)	-0.122 (-0.142 to -0.095)	0.035 (0.001 to 0.084)	0.04
Streptococcus sp. A12	0 (-0.003 to 0.002)	-0.125 (-0.145 to -0.094)	0.001 (-0.017 to 0.024)	0.804
Streptococcus sp. oral taxon 431	0 (-0.004 to 0.003)	-0.125 (-0.145 to -0.097)	0.002 (-0.025 to 0.033)	0.836
Streptococcus sp. FDAARGOS_192	-0.002 (-0.007 to 0.002)	-0.125 (-0.144 to -0.097)	0.016 (-0.015 to 0.06)	0.274
Streptococcus pneumoniae	-0.003 (-0.007 to 0.001)	-0.123 (-0.143 to -0.095)	0.02 (-0.01 to 0.055)	0.154
Streptococcus pasteurianus	0 (-0.003 to 0.003)	-0.125 (-0.144 to -0.096)	0.001 (-0.025 to 0.027)	0.93
Streptococcus thermophilus	0 (-0.003 to 0.004)	-0.126 (-0.145 to -0.097)	-0.001 (-0.031 to 0.024)	0.864
Streptococcus mitis	-0.004 (-0.01 to 0.001)	-0.122 (-0.142 to -0.095)	0.03 (-0.007 to 0.078)	0.122
Streptococcus sanguinis	0.001 (-0.001 to 0.005)	-0.129 (-0.147 to -0.101)	-0.01 (-0.044 to 0.011)	0.322
Streptococcus ilei	0 (-0.002 to 0.003)	-0.126 (-0.145 to -0.101)	-0.001 (-0.026 to 0.015)	0.78
Streptococcus parasanguinis	-0.006 (-0.012 to -0.001)	-0.121 (-0.14 to -0.09)	0.046 (0.007 to 0.096)	0.014
Streptococcus oralis	-0.001 (-0.005 to 0.002)	-0.124 (-0.145 to -0.096)	0.007 (-0.017 to 0.041)	0.508
Streptococcus cristatus	-0.004 (-0.009 to 0)	-0.122 (-0.14 to -0.096)	0.03 (0.003 to 0.073)	0.026
Streptococcus equinus	-0.001 (-0.005 to 0.001)	-0.125 (-0.145 to -0.097)	0.008 (-0.011 to 0.038)	0.404
Streptococcus australis	0 (-0.003 to 0.003)	-0.126 (-0.145 to -0.101)	0 (-0.028 to 0.024)	0.964
Streptococcus gordonii	-0.001 (-0.004 to 0.002)	-0.125 (-0.144 to -0.095)	0.005 (-0.014 to 0.033)	0.526
Streptococcus viridans	0 (-0.002 to 0.003)	-0.127 (-0.145 to -0.1)	-0.002 (-0.025 to 0.018)	0.766
Lactobacillus johnsonii	-0.002 (-0.006 to 0.001)	-0.124 (-0.144 to -0.097)	0.011 (-0.006 to 0.045)	0.23
Carnobacterium maltaromaticum	0 (-0.003 to 0.002)	-0.125 (-0.144 to -0.095)	0.002 (-0.015 to 0.026)	0.664
Aerococcus urinaeequi	-0.001 (-0.005 to 0.002)	-0.125 (-0.144 to -0.095)	0.007 (-0.018 to 0.041)	0.56
Bacillus sp. N3536	-0.001 (-0.004 to 0.002)	-0.125 (-0.144 to -0.097)	0.006 (-0.015 to 0.038)	0.504
Psychrobacillus glaciei	0 (-0.004 to 0.004)	-0.125 (-0.144 to -0.099)	0.001 (-0.032 to 0.035)	0.884
Psychrobacillus sp. AK 1817	-0.001 (-0.004 to 0.001)	-0.124 (-0.144 to -0.095)	0.006 (-0.009 to 0.035)	0.408
Sporosarcina psychrophila	-0.001 (-0.005 to 0.002)	-0.125 (-0.144 to -0.096)	0.008 (-0.017 to 0.043)	0.546
Gemella sanguinis	-0.002 (-0.006 to 0.001)	-0.124 (-0.143 to -0.097)	0.016 (-0.008 to 0.051)	0.184
[Clostridium] innocuum	0.001 (-0.003 to 0.005)	-0.127 (-0.145 to -0.098)	-0.006 (-0.04 to 0.023)	0.68
Absiella argi	-0.003 (-0.008 to 0.002)	-0.122 (-0.142 to -0.094)	0.021 (-0.018 to 0.064)	0.236
Amedibacterium intestinale	-0.005 (-0.011 to -0.001)	-0.119 (-0.139 to -0.093)	0.042 (0.011 to 0.091)	0.008
Megamonas funiformis	-0.003 (-0.007 to 0.001)	-0.123 (-0.143 to -0.093)	0.02 (-0.005 to 0.056)	0.124
Megamonas hypermegale	-0.004 (-0.009 to 0)	-0.121 (-0.141 to -0.094)	0.029 (0.002 to 0.074)	0.024
Dialister massiliensis	0 (-0.002 to 0.003)	-0.126 (-0.146 to -0.096)	0 (-0.028 to 0.019)	0.904
Finegoldia magna	-0.001 (-0.004 to 0.002)	-0.124 (-0.143 to -0.095)	0.005 (-0.013 to 0.031)	0.464
Bifidobacterium breve	0 (-0.003 to 0.003)	-0.126 (-0.144 to -0.097)	0 (-0.029 to 0.023)	0.954
Bifidobacterium pseudocatenulatum	0 (-0.002 to 0.003)	-0.127 (-0.147 to -0.1)	-0.002 (-0.028 to 0.018)	0.736
Actinomyces sp. oral taxon 414	-0.001 (-0.004 to 0.001)	-0.124 (-0.144 to -0.093)	0.006 (-0.009 to 0.033)	0.37
Schaalia odontolytica	-0.003 (-0.008 to 0.001)	-0.123 (-0.143 to -0.095)	0.02 (-0.006 to 0.06)	0.122
Rhodococcus erythropolis	-0.001 (-0.004 to 0.001)	-0.125 (-0.144 to -0.1)	0.005 (-0.008 to 0.029)	0.428
Arthrobacter sp. YC-RL1	-0.001 (-0.005 to 0.001)	-0.125 (-0.143 to -0.097)	0.009 (-0.008 to 0.043)	0.294
Rothia mucilaginosa	0 (-0.003 to 0.002)	-0.126 (-0.145 to -0.097)	0.002 (-0.014 to 0.023)	0.66
Oerskovia sp. KBS0722	-0.001 (-0.004 to 0.001)	-0.124 (-0.144 to -0.095)	0.007 (-0.007 to 0.038)	0.306
Gordonibacter pamelaeae	-0.001 (-0.005 to 0.002)	-0.125 (-0.145 to -0.097)	0.011 (-0.012 to 0.044)	0.386
Arabia massiliensis	-0.003 (-0.01 to 0.002)	-0.123 (-0.142 to -0.094)	0.026 (-0.013 to 0.08)	0.2
Adlercreutzia sp. 8CFCBH1	-0.002 (-0.006 to 0.001)	-0.124 (-0.145 to -0.093)	0.014 (-0.005 to 0.049)	0.166
Adlercreutzia equolifaciens	-0.002 (-0.006 to 0.001)	-0.124 (-0.143 to -0.095)	0.012 (-0.005 to 0.045)	0.184
Fusobacterium varium	-0.004 (-0.009 to 0)	-0.122 (-0.142 to -0.093)	0.031 (0.001 to 0.077)	0.046
Fusobacterium ulcerans	-0.002 (-0.006 to 0)	-0.123 (-0.143 to -0.092)	0.016 (-0.001 to 0.051)	0.066
Fusobacterium nucleatum	-0.002 (-0.007 to 0)	-0.124 (-0.144 to -0.096)	0.017 (-0.003 to 0.056)	0.104
Fusobacterium mortiferum	-0.004 (-0.009 to 0)	-0.122 (-0.142 to -0.091)	0.027 (-0.003 to 0.079)	0.074

Supplementary Table 41 Sensitivity analysis (exclusion of other ethnicity)
Differential metabolites between dyslipidemia and control group

Metabolites	FC	log2(FC)	P value	-LOG10(p)
13-L-Hydroperoxylinoleic acid	2.0895	1.0631	0.002868907	2.542283517
20-HETE	2.0251	1.018	0.042782005	1.368738863
3-Hydroxybenzoic acid	2.2689	1.182	0.012415455	1.906037368
Adipic acid	0.38815	-1.3653	0.037281101	1.428511266
Busulfan	2.116	1.0814	0.012775607	1.89361846
Genipin	2.5962	1.3764	0.005758925	2.239658579
L-Arogenate	2.1978	1.136	0.007926751	2.100904792
Mibefradil	2.4468	1.2909	0.044558238	1.351071995
N4-Acetylcytidine	0.37977	-1.3968	0.031448789	1.502396075
NP-016455	2.3442	1.2291	0.033357671	1.476804279

Supplementary Table 42 Sensitivity analysis (exclusion of other ethnicity)
Differential metabolites between decreased HDL and control group

Metabolites	FC	log2(FC)	P value	-LOG10(p)
(2S,3S,4S,8R,9S,13R,14R,15R,16R)-3,4,8,14,15-pentahydroxy-2,13,16-trimethyl-6-methylidene-10-oxatetracyclo[7.6.1.02,7.012,16]hexadecan-	0.22942	-2.1239	0.006249394	2.204162092
(plusmn)5-HETrE	2.5926	1.3744	0.003141393	2.502877763
20-HETE	3.8167	1.9323	0.002198325	2.657908133
21-Deoxycortisol	4.3982	2.1369	0.01575698	1.802527016
3,3',4,4',5,5'-hexabromo-1H,1'H-2,2'-bipyrrole	0.47174	-1.0839	0.047281673	1.325307162
4-Hydroxytamoxifen	0.27516	-1.8617	0.041742306	1.379423559
5-[(1S,2R,4aR)-5-(Hydroxymethyl)-1,2,4a-trimethyl-1,2,3,4,4a,7,8,8a-octahydro-1-naphthalenyl]-3-methylpentanoic acid	2.3648	1.2417	0.046942927	1.328429838
8-Isoprostane	0.30278	-1.7237	0.01068623	1.971175496
8,9-DiHETrE	2.4873	1.3146	0.041116069	1.385988415
Avocadene 1-acetate	2.2689	1.182	0.027631771	1.558591286
beta-Alanyl-L-arginine	2.2453	1.1669	0.028835654	1.540070201
bk-EABDI	0.22899	-2.1266	0.02272507	1.643494776
Capsaicin	2.462	1.2998	0.045399687	1.342947142
Cortexolone	2.4818	1.3114	0.016380257	1.785679279
Corydaline	0.33498	-1.5778	0.00855839	2.067607941
Cytochalasin B	2.6176	1.3882	0.024497427	1.610879522
Diphenhydramine	0.47157	-1.0844	0.034805241	1.45835535
Geranic acid	2.2504	1.1702	0.049743036	1.303267709
Homogentisic acid	0.4277	-1.2253	0.008023444	2.095639168
L-Ascorbic acid, 6-octadecanoate	2.1951	1.1343	0.018225228	1.739327036
Lipoxin A4	2.9346	1.5532	0.032316093	1.490581151
Magnesium protoporphyrin	2.088	1.0621	0.033175661	1.479180413
NCGC00180087-02!5-(hydroxymethyl)-3-(1-hydroxy-4-methylhexyl)oxolan-2-one	0.34958	-1.5163	0.04332701	1.363241283
NCGC00380117-01_C27H41NO4_(7E)-3-Isobutyl-4,5,8,12,12-pentamethyl-3,3a,4,6a,9,10,10a,13a,14,15-decahydro-1H-[1,3]dioxolo[7,8]cycloundeca[1,2-d]isoindole-1,16(2H)-dione	2.1691	1.1171	0.014143143	1.849454064
NCGC00381071-01!1,12-dihydroxy-1,6,12,17,23,28-hexazacyclotritriacontane-2,5,13,16,24,27-hexone	2.8084	1.4898	0.025978202	1.585390917
NP-003964	2.1528	1.1062	0.002917281	2.535021766
NP-013808	0.25427	-1.9756	0.003245535	2.488713768
PGF2alpha diethyl amide	3.5912	1.8445	0.041377478	1.383235987
Poly THF n5	3.0542	1.6108	0.004922643	2.307801699
Polylimonene	2.8072	1.4892	0.045641698	1.340638211
Prostaglandin B1	2.3908	1.2575	0.017499821	1.756966386
Salsolinol	0.2445	-2.0321	0.00551678	2.258314347
Trehalose dihydrate	0.44377	-1.1721	0.041893604	1.377852275

Supplementary Table 43 Sensitivity analysis (exclusion of other ethnicity)
Differential metabolites between elevated LDL and control group

Metabolites	FC	log2(FC)	P value	-LOG10(p)
3-Hydroxybenzoic acid	3.8551	1.9468	0.000122595	3.911526123
Adipic acid	0.29602	-1.7562	0.026789345	1.572037906
Busulfan	2.313	1.2098	0.001082595	2.965533978
Citrinin	2.016	1.0115	0.028162996	1.550321152
Enterodiol	2.0475	1.0339	0.03733474	1.427886873
Ethylmorphine	0.17468	-2.5172	0.017548977	1.755748191
Gallic acid	2.5434	1.3467	0.000132657	3.877268886
Genipin	2.0953	1.0672	0.002255566	2.646744371
Glutamine	0.43253	-1.2091	0.02798633	1.553054047
N-(4-isopropylphenyl)-2-{[4-(3-methoxypropyl)-5-(2-methyl-1,3-thiazol-4-yl)-4H-1,2,4-triazol-3-yl]thio}acetamide	0.48807	-1.0348	0.018275814	1.738123275
NCGC00381380-01!(2R)-3-hydroxy-2-[(2-hydroxybenzoyl)amino]propanoic acid	2.4659	1.3021	0.044640322	1.350272677
Nevskin	0.23101	-2.114	0.026105221	1.583272624
NP-016455	2.7326	1.4503	0.019403156	1.71212763
NP-017667	2.1967	1.1353	0.005201427	2.283877491
Vanillylmandelic acid	5.4357	2.4425	0.015405944	1.812311684

Supplementary Table 44 Sensitivity analysis (exclusion of other ethnicity)
Differential metabolites between elevated TC and control group

Metabolites	FC	log2(FC)	P value	-LOG10(p)
(2S,5S)-trans-Carboxymethylproline	3.9432	1.9794	0.043342578	1.363085256
2-[6-(diethylamino)purin-9-yl]-5-(hydroxymethyl)oxolane-3,4-diol	0.41802	-1.2584	0.004206179	2.37611223
2-Deacetoxy taxinine B	2.066	1.0468	0.021445979	1.668654128
2-Methyl-3-hydroxy-5-formylpyridine-4-carboxylate	2.5224	1.3348	0.006258266	2.203545971
3-Hydroxybenzoic acid	4.5872	2.1976	0.000245848	3.609333153
3-Hydroxymethylglutaric acid	2.2881	1.1941	0.027007559	1.568514662
3,5,7,15-tetraacetoxy-2-hydroxy-8-isobutyroyloxy-9,14-dioxojatropha-6(17),11E-diene (2)	2.0088	1.0063	0.019636236	1.706941761
6-Hydroxynicotinic acid	2.0998	1.0703	0.007220699	2.14142076
7-Sulfocholic acid	0.4659	-1.1019	0.029709138	1.527109943
Adipic acid	0.24669	-2.0192	0.029390441	1.531793896
Asperuloside	2.833	1.5023	0.000410035	3.387178588
Beta-Tyrosine	0.38945	-1.3605	0.003746689	2.426352309
Bioresmethrin	0.43925	-1.1869	0.033023088	1.481182312
Busulfan	2.7745	1.4722	0.000321762	3.492464684
CAY10498	0.47774	-1.0657	0.024356449	1.613386022
Citalopram	0.3961	-1.3361	0.015747603	1.802785547
Citrinin	2.4533	1.2947	0.002087738	2.680323995
Dipropyleneglycol dibenzoate	0.49852	-1.0043	0.017624159	1.753891604
Enterodiol	2.0506	1.0361	0.041003543	1.38717862
Galacturonic acid	2.0685	1.0486	0.005597614	2.251997091
Gallic acid	3.2961	1.7208	1.18E-05	4.92936783
Ganoderic acid G	2.205	1.1408	0.013278426	1.876853404
Genipin	2.653	1.4076	0.000270054	3.568550086
Glutamine	0.32235	-1.6333	0.018560144	1.731418668
Indinavir	0.45193	-1.1458	0.002905932	2.536714571
L-Arogenate	2.157	1.109	0.005089223	2.293348559
Lopinavir	0.32287	-1.631	0.008142492	2.089242677
N-(4-isopropylphenyl)-2-{[4-(3-methoxypropyl)-5-(2-methyl-1,3-thiazol-4-yl)-4H-1,2,4-triazol-3-yl]thio}acetamide	0.42035	-1.2503	0.012859332	1.890781601
N1-Benzyl-2-[(2-oxo-3-piperidyl)carbonyl]hydrazine-1-carbothioamide	2.033	1.0236	0.011284082	1.947533749
NCGC00381061-01_C30H51N5O9_Pyrrolo[1,2-d][1,4,7,10,13,16]oxapentaazacyclononadecine-1,4,7,10,14,17(11H,16H)-hexone, 16-(2,3-dihydroxypropyl)dodecahydro-	0.35466	-1.4955	0.033549398	1.474315274
NCGC00381380-01!(2R)-3-hydroxy-2-[(2-hydroxybenzoyl)amino]propanoic acid	3.1909	1.674	0.007503294	2.124748018
NCGC00384635-01_C27H34O8_Methyl [(1S,3S,7R,8R,9R,12S,13S)-13-(3-furyl)-6,6,8,12-tetramethyl-17-methylene-5,15-dioxo-2,14-dioxatetracyclo[7.7.1.0~1,12~.0~3,8~]heptadec-7-yl](hydroxy)acetate	2.524	1.3357	0.040900202	1.388274543
Neamine	0.436	-1.1976	0.025327884	1.596401086
NP-012268	0.28827	-1.7945	0.04665657	1.33108719
NP-016455	3.3851	1.7592	0.002754292	2.559990002
NP-017667	2.7231	1.4452	0.002158816	2.665784393
Octabenzone	0.32854	-1.6058	0.006612151	2.17965721
Pleiomutinine	0.42067	-1.2492	0.041121176	1.385934472
Probucol	2.1928	1.1328	0.01127998	1.947691679
Quinate	3.6908	1.8839	0.005752235	2.240163404
Thalsimine	2.5103	1.3279	0.012936582	1.888180446
Vanillylmandelic acid	6.6189	2.7266	0.005139612	2.289069672

Supplementary Table 45 Sensitivity analysis (exclusion of other ethnicity)
Differential metabolites between elevated TG and control group

Metabolites	FC	log2(FC)	P value	-LOG10(p)
(2E)-N-(4-acetamidobutyl)-3-(4-hydroxy-3-methoxyphenyl)prop-2-enamide	0.40516	-1.3034	0.008330759	2.079315405
13-L-Hydroperoxylinoleic acid	3.2388	1.6955	0.003791785	2.421156302
2-[6-(diethylamino)purin-9-yl]-5-(hydroxymethyl)oxolane-3,4-diol	0.47362	-1.0782	0.020495342	1.68834484
3,4-Dihydroxybenzeneacetic acid	2.0095	1.0068	0.01735818	1.760495821
3alpha,12alpha-Dihydroxy-5beta-chol-6-enoate	2.1992	1.137	0.002947089	2.530606796
9(S)-HPODE	2.2686	1.1818	0.018501556	1.732791738
Bioresmethrin	0.43533	-1.1998	0.009682548	2.014010321
CAY10498	0.35364	-1.4996	0.002311342	2.636135833
DIHYDROCELASTRYL DIACETATE	2.2425	1.1651	0.000188516	3.724652716
Dipropyleneglycol dibenzoate	0.4764	-1.0698	0.031184674	1.506058795
dTDP-D-glucose	2.1232	1.0863	0.049029921	1.30953881
Gallic acid	2.1109	1.0778	0.006841478	2.164850079
Genipin	4.0058	2.0021	0.005165796	2.286862709
L-Arogenate	3.2819	1.7146	0.004750973	2.323217398
L-Carnitine	2.2681	1.1815	0.03436922	1.463830324
N2,N5-Dibenzylpyrrolidine-2,5-dicarboxamide	0.4899	-1.0294	0.008037055	2.094903074
NCGC00381071-01!1,12-dihydroxy-1,6,12,17,23,28-hexazacyclotritriacontane-2,5,13,16,24,27-hexone	2.1389	1.0968	0.025730318	1.589554851
Neamine	0.43111	-1.2139	0.040020536	1.397717098
Octabenzone	0.2853	-1.8095	0.009633105	2.01623372
PGF2alpha diethyl amide	2.8118	1.4915	0.04440756	1.352543086
Porphobilinogen	2.7586	1.4639	0.000222853	3.651980547
Serine-Cholic Acid	2.2619	1.1775	0.033482501	1.475182112
Taurohyocholate	2.1182	1.0828	0.013726001	1.862455987
vanillylmandelate	0.38883	-1.3628	0.001974044	2.704643232

Supplementary Table 46 Differential gut microbiota between dyslipidemia and control group in validation cohort

Species	LDA	P.adj
Eubacterium callanderi	3.228130966	0.007831667
Lactiplantibacillus pentosus	3.549002952	0.003369332
Porphyromonas gingivalis	3.260277832	0.005588374

Supplementary Table 47 Differential gut microbiota between decreased HDL and control group in validation cohort

Species	LDA	P.adj
[Ruminococcus] gnavus	3.014453918	0.008894475
Eubacterium callanderi	2.771588897	0.00294183

Supplementary Table 48 Differential gut microbiota between elevated LDL and control group in validation cohort

Species	LDA	P.adj
Acidipropionibacterium acidipropionici	3.232036508	0.003495858
Actinomyces sp. Chiba101	3.457623841	0.002943617
Clostridium isatidis	3.576364711	0.008888021
Lactiplantibacillus pentosus	4.025343621	0.001821119
Lentilactobacillus buchneri	2.949472253	0.002386769
Serratia marcescens	3.216682737	0.006428155

Supplementary Table 49 Differential gut microbiota between elevated TC and control group in validation cohort

Species	LDA	P.adj
Acidipropionibacterium acidipropionici	3.084108103	0.009174363
Clostridiales bacterium CCNA10	3.317069959	0.004007037
Enterococcus saigonensis	2.972079927	0.008279889
Lachnospira eligens	3.02144535	0.006048944
Lachnospiraceae bacterium GAM79	3.161978474	0.006676271
Lactiplantibacillus pentosus	3.732647767	0.007688549
Raoultella planticola	2.764073622	0.005959906
Staphylococcus kloosii	2.798265093	0.006361843

Supplementary Table 50 Differential gut microbiota between elevated TG and control group in validation cohort

Species	LDA	P.adj
[Clostridium] scindens	3.334367634	0.001141792
[Ruminococcus] gnavus	2.950010476	0.002304406
Alistipes shahii	2.969015059	0.007043373
Blautia argi	3.19539092	0.009949933
Dysosmobacter welbionis	2.910939927	0.006703482
Enterocloster clostridioformis	3.217512864	0.007566975
Fusobacterium ulcerans	3.183870523	0.008523693
Haemophilus influenzae	3.12622987	0.008838327
Lachnospira eligens	3.186263493	0.008146418
Lactiplantibacillus pentosus	3.306956031	0.000602109

Supplementary Table 51 Differential metabolites between dyslipidemia and control group in validation cohort

Metabolites	FC	log2(FC)	P value	-LOG10(p)
16(R)-HETE	3.8443	1.9427	0.011348291	1.945069539
5,6-DHET	2.9499	1.5606	0.033154589	1.479456348
8,9-DiHETrE	2.945	1.5583	0.019729663	1.704880327
Arachidonic acid	2.4326	1.2825	0.024652585	1.608137538
Celastrol	0.43781	-1.1916	0.00243927	2.612740162
Chenodeoxycholic acid glycine conjugate	0.44776	-1.1592	0.047358027	1.324606402
Dihomo-gamma-linolenate	2.1422	1.0991	0.001522586	2.817418249
Dinoseb	2.0502	1.0357	0.027324454	1.563448508

Supplementary Table 52 Differential metabolites between decreased HDL and control group in validation cohort

Metabolites	FC	log2(FC)	P value	-LOG10(p)
16-Hydroxy hexadecanoic acid	2.3472	1.231	0.039220755	1.406484045
3-Oxo-5beta-cholanate	2.0889	1.0627	0.032668847	1.485866191
Alpha-dimorphecolic acid	2.0213	1.0153	0.020601022	1.686111233
Cyclopentolate	2.2458	1.1672	0.030048679	1.522174619
Cycloposine	2.3424	1.228	0.040573015	1.391762724
D-Glucurono-6,3-lactone	3.1014	1.6329	0.015665783	1.805047886
Dinoseb	3.4603	1.7909	0.007441191	2.128357575
Hesperetin	0.11221	-3.1558	0.038210744	1.417814511
JWH 122 N-(5-hydroxypentyl) metabolite	3.2588	1.7044	0.030711152	1.512703893
N-(alpha-Linolenoyl) Tyrosine	0.46443	-1.1065	0.01775607	1.750653161
N-acetyl-2-carboxy Benzenesulfonamide	2.6106	1.3844	0.010902313	1.962481367
Naringenin	0.2777	-1.8484	0.03771807	1.42345054

Supplementary Table 53 Differential metabolites between elevated LDL and control group in validation cohort

Metabolites	FC	log2(FC)	P value	-LOG10(p)
(2S,5S)-trans-Carboxymethylproline	0.45594	-1.1331	0.00312273	2.505465578
(S)-2-Amino-3-(1H-indol-3-yl)propanamide hydrochloride	2.5179	1.3322	0.029974143	1.523253221
2-Hydroxy-6-pentadecylbenzoic acid	0.28475	-1.8122	0.003666206	2.435783175
5,6-DHET	5.1864	2.3747	0.011113129	1.954163641
8,9-DiHETrE	5.1262	2.3579	0.009172586	2.037508191
Arachidonic acid	3.5368	1.8225	0.01545083	1.811048198
Celastrol	0.34116	-1.5515	0.025770551	1.588876291
Chenodeoxycholic acid glycine conjugate	0.20874	-2.2603	0.026706174	1.573388318
Cholesterol sulfate	0.28104	-1.8311	0.02911738	1.535847708
Dihomo-gamma-linolenate	2.9974	1.5837	0.004555734	2.341441639
Glycocholic acid	0.28193	-1.8266	0.049656691	1.304022228
Indole-3-carboxylic acid	2.689	1.4271	0.02333394	1.632011916
Indolelactic acid	2.3462	1.2303	0.012699793	1.896203351
N-(2-fluro-ethyl) arachidonoyl amine	0.31016	-1.6889	0.001120314	2.95066034
Phloretin	0.42299	-1.2413	0.017339528	1.760962735
S-Allylcysteine	3.2742	1.7111	0.014154371	1.849109436
S-Methyl-L-methionine	0.42165	-1.2459	0.048968889	1.310079747
Taurochenodesoxycholic acid	0.13584	-2.88	0.008430981	2.074121866
Tonantzitlolone A	2.1536	1.1067	0.003380592	2.471007261

Supplementary Table 54 Differential metabolites between elevated TC and control group in validation cohort

Metabolites	FC	log2(FC)	P value	-LOG10(p)
16(R)-HETE	4.7991	2.2628	0.016037436	1.794865068
5,6-DHET	4.018	2.0065	0.00065128	3.186232019
8,9-DiHETrE	4.0216	2.0078	0.002937188	2.532068263
Arachidonic acid	3.0757	1.6209	0.040196945	1.395806955
Celastrol	0.4131	-1.2754	0.022512805	1.647570388
Citramalic acid	2.1256	1.0879	0.028914047	1.538891116
Dihomo-gamma-linolenate	2.3443	1.2291	0.001451121	2.838296356
Marindinin	0.27529	-1.861	0.012106721	1.916973481
Prostaglandin E1	2.9764	1.5736	0.028355836	1.547357542
S-Allylcysteine	2.1712	1.1185	0.030133102	1.520956161

Supplementary Table 55 Differential metabolites between elevated TG and control group in validation cohort

Metabolites	FC	log2(FC)	P value	-LOG10(p)
12,13-DHOME	2.3455	1.2299	0.014000726	1.853849446
16(R)-HETE	3.0754	1.6208	0.016807835	1.774488234
4-{[5-(7-hydroxy-5,5,8a-trimethyl-2-methylidene-decahydronaphthalen-1-yl)-3-methylpentyl]oxy}-4-	2.132	1.0922	0.006042828	2.218759779
4-Hydroxytamoxifen	0.41442	-1.2708	0.038120367	1.418842922
5-[(E)-2-(4-hydroxy-3-methoxyphenyl)ethenyl]benzene-1,3-diol	0.31588	-1.6625	0.022690044	1.644164655
7-{2,6-Dimethyl-8-[(2-methylbutanoyl)oxy]-1,2,6,7,8,8a-hexahydro-1-naphthalenyl}-3,5-dihydroxyheptanoic acid	2.0901	1.0635	0.017193587	1.764633507
8,9-DiHETrE	2.215	1.1473	0.043128936	1.365231254
9,10-DHOME	2.1211	1.0848	0.040539125	1.392125626
Alpha-dimorphecolic acid	4.8323	2.2727	0.034949192	1.45656286
Celastrol	0.40048	-1.3202	0.007415472	2.129861204
Dihomo-gamma-linolenate	2.0453	1.0323	0.039189866	1.406826225
Indole-3-carboxylic acid	2.1242	1.0869	0.019408349	1.712011403
L-Histidine	2.1926	1.1326	0.02961045	1.528554987
Maslinic acid	2.3808	1.2514	0.013715712	1.862781639
NCGC00380891-01_C24H30O5_2,4-Octadienoic acid, 6-methyl-, (1R,2S,7R,8aR)-7-(1-formylethenyl)-1,2,6,7,8,8a-hexahydro-7-hydroxy-1,8a-dimethyl-6-oxo-2-naphthalenyl ester, (2E,4E,6R)-	2.0128	1.0092	0.019458979	1.710879942
Prostaglandin E1	2.1956	1.1346	0.046344424	1.334002507
Sparfloxacin	2.2391	1.1629	0.000187127	3.727863734
Thalsimine	2.4462	1.2905	0.041538096	1.381553416

STROBE Statement—checklist of items that should be included in reports of observational studies

	Item No.	Recommendation	Page No.
**Title and abstract**	1	(*a*) Indicate the study's design with a commonly used term in the title or the abstract	2
		(*b*) Provide in the abstract an informative and balanced summary of what was done and what was found	2
**Introduction**			
Background/rationale	2	Explain the scientific background and rationale for the investigation being reported	3
Objectives	3	State specific objectives, including any prespecified hypotheses	4
**Methods**			
Study design	4	Present key elements of study design early in the paper	11
Setting	5	Describe the setting, locations, and relevant dates, including periods of recruitment, exposure, follow-up, and data collection	11-12
Participants	6	(*a*) *Cohort study*—Give the eligibility criteria, and the sources and methods of selection of participants. Describe methods of follow-up<br>*Case-control study*—Give the eligibility criteria, and the sources and methods of case ascertainment and control selection. Give the rationale for the choice of cases and controls<br>*Cross-sectional study*—Give the eligibility criteria, and the sources and methods of selection of participants	11-12
		(*b*) *Cohort study*—For matched studies, give matching criteria and number of exposed and unexposed<br>*Case-control study*—For matched studies, give matching criteria and the number of controls per case	NA
Variables	7	Clearly define all outcomes, exposures, predictors, potential confounders, and effect modifiers. Give diagnostic criteria, if applicable	12-14
Data sources/ measurement	8*	For each variable of interest, give sources of data and details of methods of assessment (measurement). Describe comparability of assessment methods if there is more than one group	11-14
Bias	9	Describe any efforts to address potential sources of bias	16
Study size	10	Explain how the study size was arrived at	11-12

Continued on next page

Quantitative variables	11	Explain how quantitative variables were handled in the analyses. If applicable, describe which groupings were chosen and why	15
Statistical methods	12	(*a*) Describe all statistical methods, including those used to control for confounding	15-16
		(*b*) Describe any methods used to examine subgroups and interactions	15-16
		(*c*) Explain how missing data were addressed	11
		(*d*) *Cohort study*—If applicable, explain how loss to follow-up was addressed<br>*Case-control study*—If applicable, explain how matching of cases and controls was addressed<br>*Cross-sectional study*—If applicable, describe analytical methods taking account of sampling strategy	11-12
		(*e*) Describe any sensitivity analyses	16
**Results**			
Participants	13*	(a) Report numbers of individuals at each stage of study—eg numbers potentially eligible, examined for eligibility, confirmed eligible, included in the study, completing follow-up, and analysed	4
		(b) Give reasons for non-participation at each stage	NA
		(c) Consider use of a flow diagram	24
Descriptive data	14*	(a) Give characteristics of study participants (eg demographic, clinical, social) and information on exposures and potential confounders	29
		(b) Indicate number of participants with missing data for each variable of interest	11
		(c) *Cohort study*—Summarise follow-up time (eg, average and total amount)	NA
Outcome data	15*	*Cohort study*—Report numbers of outcome events or summary measures over time	NA
		Case-control study—Report numbers in each exposure category, or summary measures of exposure	NA
		Cross-sectional study—Report numbers of outcome events or summary measures	4
Main results	16	(*a*) Give unadjusted estimates and, if applicable, confounder-adjusted estimates and their precision (eg, 95% confidence interval). Make clear which confounders were adjusted for and why they were included	25
		(*b*) Report category boundaries when continuous variables were categorized	NA
		(*c*) If relevant, consider translating estimates of relative risk into absolute risk for a meaningful time period	NA

Continued on next page

Other analyses	17	Report other analyses done—eg analyses of subgroups and interactions, and sensitivity analyses	5-7
**Discussion**			
Key results	18	Summarise key results with reference to study objectives	9
Limitations	19	Discuss limitations of the study, taking into account sources of potential bias or imprecision. Discuss both direction and magnitude of any potential bias	11
Interpretation	20	Give a cautious overall interpretation of results considering objectives, limitations, multiplicity of analyses, results from similar studies, and other relevant evidence	9-10
Generalisability	21	Discuss the generalisability (external validity) of the study results	10
**Other information**			
Funding	22	Give the source of funding and the role of the funders for the present study and, if applicable, for the original study on which the present article is based	17

*Give information separately for cases and controls in case-control studies and, if applicable, for exposed and unexposed groups in cohort and cross-sectional studies.
